# *Bacillus subtilis* RNase Y Activity In Vivo Analysed by Tiling Microarrays

**DOI:** 10.1371/journal.pone.0054062

**Published:** 2013-01-10

**Authors:** Soumaya Laalami, Philippe Bessières, Anna Rocca, Léna Zig, Pierre Nicolas, Harald Putzer

**Affiliations:** 1 CNRS UPR 9073 (affiliated with University Paris Diderot, Sorbonne Paris Cité), Institut de Biologie Physico-Chimique, Paris, France; 2 INRA UR1077, Mathématique Informatique et Génome, Jouy-en-Josas, France; University of Groningen, Groningen Institute for Biomolecular Sciences and Biotechnology, The Netherlands

## Abstract

RNase Y is a key endoribonuclease affecting global mRNA stability in *Bacillus subtilis*. Its characterization provided the first evidence that endonucleolytic cleavage plays a major role in the mRNA metabolism of this organism. RNase Y shares important functional features with the RNA decay initiating RNase E from *Escherichia coli*, notably a similar cleavage specificity and a preference for 5′ monophosphorylated substrates. We used high-resolution tiling arrays to analyze the effect of RNase Y depletion on RNA abundance covering the entire genome. The data confirm that this endoribonuclease plays a key role in initiating the decay of a large number of mRNAs as well as non coding RNAs. The downstream cleavage products are likely to be degraded by the 5′ exonucleolytic activity of RNases J1/J2 as we show for a specific case. Comparison of the data with that of two other recent studies revealed very significant differences. About two thirds of the mRNAs upregulated following RNase Y depletion were different when compared to either one of these studies and only about 10% were in common in all three studies. This highlights that experimental conditions and data analysis play an important role in identifying RNase Y substrates by global transcriptional profiling. Our data confirmed already known RNase Y substrates and due to the precision and reproducibility of the profiles allow an exceptionally detailed view of the turnover of hundreds of new RNA substrates.

## Introduction

In prokaryotic cells, mRNA half-lives range from seconds to over an hour. Controlling the life span of a messenger can thus be an efficient means to control gene expression. Recent progress has provided new insight into the enzymes involved in the initiating phase of RNA degradation.

In *E. coli,* mRNA decay is primarily initiated through endonucleolytic cleavage by the single-strand specific RNase E at one or more internal sites, followed by rapid 3′ exonucleolytic degradation of the decay intermediates [1]. Internal cleavage of primary transcripts by RNase E can occur directly [2],[3] or be stimulated by prior conversion of the 5′ triphosphate moiety to a 5′ monophosphate by the pyrophosphohydrolase RppH [4].

However, despite its central role in mRNA decay in *E. coli*, RNase E is absent from many eubacteria including the Gram positive model bacterium *B. subtilis.* This organism instead possesses a pair of paralogous RNases, J1 and J2, which combine an RNase E-like endonucleolytic [5] and a 5′ exonucleolytic activity that requires a 5′ monophosphate group [6],[7]. The latter can be provided by *B. subtilis* RppH (previously named YtkD), a homolog of the *E. coli* pyrophosphohydrolase RppH [8] or by endonucleolytic cleavage. The 5′ exonucleolytic activity of the essential RNase J1 which forms a complex with the non-essential RNase J2 [9] can thus play an important role in initiating the degradation of an original transcript whose 5′ end has been converted to a 5′ P. Transcriptome and proteome studies have shown that RNases J1/J2 can influence the expression of hundreds of genes [10],[11]. However, in a double mutant (expressing no RNase J2 and strongly reduced levels of RNase J1) global mRNA stability is only slightly increased [10], a finding not compatible with a key role for RNase J in initiating mRNA decay on a global scale that would be comparable with the role of RNase E in *E. coli*. In extrapolation, this also suggests that the endonucleolytic activity of RNases J1/J2 as well as the 5′ P conversion of native transcripts by *B. subtilis* RppH occurs on a more limited scale.

Evidence that endonucleolytic cleavage plays an important role in initiating mRNA decay in *B. subtilis* came through the characterisation of the novel endoribonuclease Y (previously named YmdA) [12]. Indeed, RNase Y shares surprising functional homology with RNase E despite a complete lack of sequence similarity. Both nucleases have a similar cleavage specificity and their activity can be stimulated by a 5′ monophosphorylated substrate. In addition, depletion of RNase Y increased global mRNA stability more than two-fold [12]. Other similarities with RNase E include a localization at the membrane [13] and the potential to form a complex – the «degradosome» - including PNPase and glycolytic enzymes [14],[15]. However, the *B. subtilis* degradosome complex cannot be isolated in the absence of cross-linking reagents and therefore its existence *in vivo* remains uncertain [9],[16].

Initiation of mRNA decay might thus be more similar between *E. coli* and *B. subtilis* than previously assumed, with an endonucleolytic cleavage being the crucial step (for a recent review, see [17]). In this model, the 5′ exonucleolytic activity of RNase J would be mainly responsible for degrading cleavage intermediates initially generated by RNase Y and, to a lesser extent, by RNase J or another endoribonuclease. Exonucleolytic degradation from the 5′ end is especially useful in an organism like *B. subtilis* that, in contrast to *E. coli*, does not have a very efficient polyadenylation pathway to help degrade structured RNAs (e.g. transcription terminators) exonucleolytically from the 3′ end. In addition, 2–3% of genes in *Bacillus* are subject to various transcriptional attenuation mechanisms [18] that produce large amounts of prematurely terminated leader transcripts. In agreement, the concerted action of RNase Y and RNase J in exonucleolytic mode has been implicated in the turnover of S-adenosyl methionine (SAM)-dependent riboswitches [12]. Other examples of RNase Y action include a regulatory processing of the *gapA* operon transcript [14], initiation of the *rpsO* mRNA decay [19] and the uncoupling of the expression of translation factor IF3 from that of the ribosomal proteins in the *infC-rpmI-rplT* operon [20]. In *S. aureus*, RNase Y is important for the processing of virulence related mRNAs [21].

Here we analysed the effect of RNase Y depletion on the relative abundance of all RNA transcripts in the cell, using tiling arrays with a resolution of 22 nucleotides [22]. We compared our results to two other RNase Y depletion studies, a recent classical transcriptome analysis [23] and a study carried out in parrallel to this work [11]. Surprisingly, we found very significant differences between our results and those obtained in those studies.

## Results

### Experimental Strategy

In order to analyse the effect of RNase Y on the global transcription pattern we decided to partially deplete the nuclease to a point where the enzyme becomes limiting for growth. In this way, we minimized growth rate effects and the measurements are made under near steady state conditions. Partial depletion of the enzyme has the potential drawback that the RNase Y substrates with the highest affinity might not be detected. However, by carefully choosing the time point for taking the samples, based on the effect of RNase Y on known RNA substrates, we obtained a highly reproducible data set which allows to analyse the effect of RNase Y on global transcription with unprecedented precision.

For the study described here we used strain SSB447 where the *rny* gene can be conditionally expressed from the IPTG inducible P*spac* promoter [12]. We had previously noticed a significant polar effect on the transcription of the adjacent *ymdB* gene, notably in the absence of IPTG. A *ymdB* null mutation has recently been shown to strongly induce the σ^D^ regulon and drastically reduce biofilm related gene expression [24]. However, under our conditions of RNase Y depletion the levels of the *sigD* (σ^D^) and *hag* (flagellin) mRNAs were slightly reduced or unchanged (Table S1), respectively, compared to the corresponding levels in the *ymdB* mutant which were induced 14- and more than 30-fold [24]. This suggested that the YmdB protein was not strongly reduced under our conditions of RNase Y depletion and we decided not to compensate the reduced transcription of *ymdB*.

**Figure 1:**
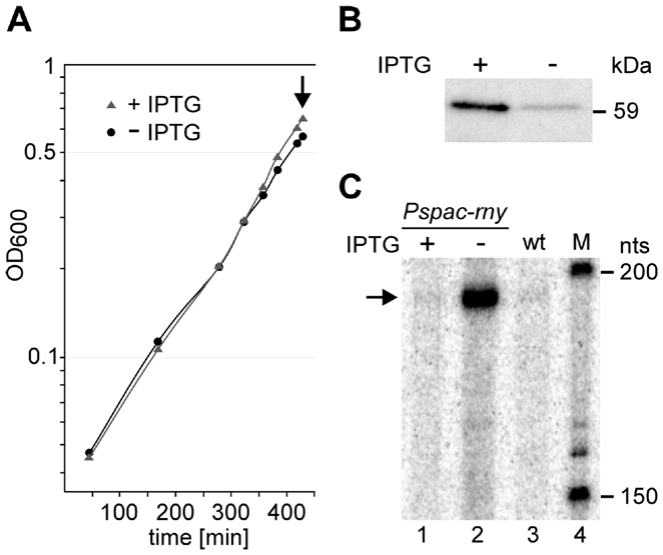
Culture conditions and control of RNase Y depletion. A) Strain SSB447 containing the *rny* gene under the control of the inducible *Pspac* promoter was grown in the presence and absence of IPTG. When growth began to slow down due to the absence of inducer, cells were harvested (arrow). B) Depletion of RNase Y was quantified by Western blotting using antibodies against *B. subtilis* RNase Y. C) The efficiency of RNase Y depletion was checked by quantifying the stabilization of the *yitJ* SAM riboswitch RNA by Northern blot in the cells grown without IPTG (lane 2) as described previously [12] and compared to the same strain grown in the presence of IPTG (lane 1) and to the wild type strain (lane 3). M (lane 4) is a size marker.

The depletion strain SSB447 also contained plasmid pMAP65 [25], which provided additional copies of the LacI repressor for a tight regulation of the P*spac* promoter. Under our experimental conditions, i.e. cell harvest following the first detectable slow-down in growth (Fig. 1A), the *rny* mRNA level in the cell was reduced 100-fold in the depleted strain (grown in the absence of IPTG) compared to the same strain grown in the presence of IPTG (Table S2). This translated into a six-fold reduction of the RNase Y level as judged by Western blot (Fig. 1B). We further measured the abundance of the SAM riboswitch RNA of the *yitJ* gene which is sensitive to the level of RNase Y in the cell [12]. Wild type cells or depletion strain SSB447 grown in the presence of IPTG showed a barely detectable level of riboswitch RNA (Fig. 1C, lanes 1 and 3) which was strongly increased when RNase Y was depleted (Fig. 1C, lane 2).

### Rnase Y Affects Gene Expression On A Genome-Wide Scale

We compared the transcript profile of total RNA preparations from duplicate cultures of the depletion strain SSB447 grown in the absence and presence of IPTG by using tiling array technology (BaSysBio T2 design from Roche-Nimblegen) as described previously [22]. The four whole-genome transcriptional landscapes reconstructed from the raw data are shown in Figure S1.

The high reproducibility between biological replicates and the large number of genes impacted by the RNase depletion made it possible, despite the availability of only two biological replicates, to apply a stringent control of the False Discovery Rate (FDR) when calling differentially expressed genes. Namely, we selected a cut-off of 0.1 on the local FDR such that the gene with the less significant p-value that we called differentially expressed has still a probability of only 0.1 to be a false positive. This analysis led to an initial set of 2884 differentially expressed transcripts (genes and un-annotated segments) accounting for ∼51% of the annotated *B. subtilis* protein coding genes in which the estimated average FDR is 0.047.

**Figure 2:**
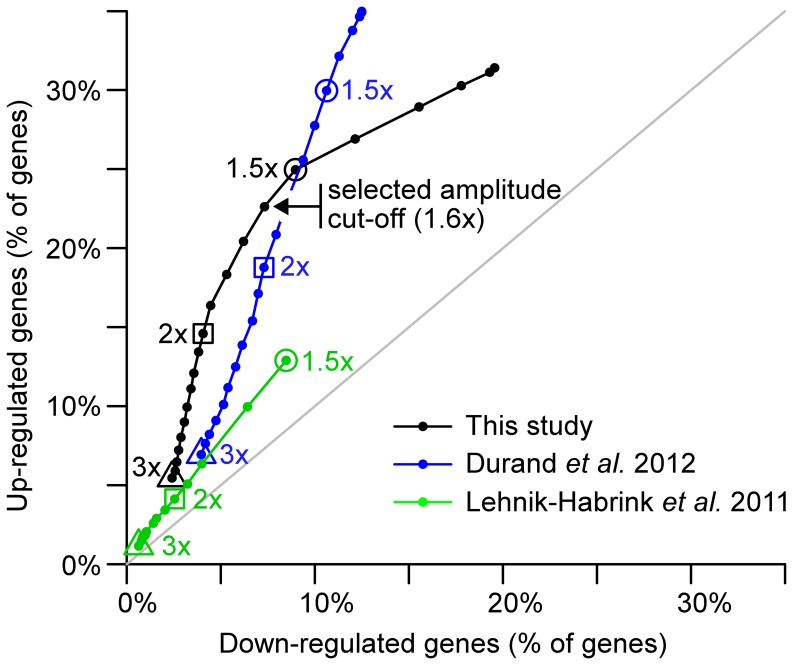
Numbers of up-regulated versus down-regulated genes as a function of the amplitude cut-off applied to the observed effect of RNase Y depletion. The three studies are represented by different colors (our study, black; Durand *et al*. 2012, blue; Lehnik-Habrink *et al.* 2011, green). Numbers reported on x- and y-axes are based on protein coding genes and expressed as a percentage of the total protein coding gene pool in the genome. Three amplitude cut-offs are highlighted : 3x, triangles; 2x, squares; 1.5x, circles. For our and Durand *et al.*, 2012 studies the numbers represented in this plot correspond to genes that also satisfied a FDR criterion warranting the statistical significance of the detected differential expression. Such a statistical analysis was not reported in Lehnik-Habrink et al. 2011. In our study, the change of slope reflecting a preponderance of up-regulated genes among high amplitude changes and down-regulated genes among low amplitude changes lead us set the cut-off to 1.6x (black arrow).

In order to subset only the most biologically relevant genes within this initial set of differentially expressed genes we applied a second cut-off on the amplitude of the measured effects. A plot of the evolution of the number of up- versus down-regulated genes when the amplitude cut-off is lowered (Fig. 2) revealed a break point in the slope at a gene expression variation of about 1.6x. Above this threshold up-regulated genes were more represented than down-regulated genes whereas an excess of down-regulated genes was visible below this threshold. Since down regulation of specific transcripts could be considered largely indirect, but upregulated transcripts were almost always direct substrates for RNase Y (see below), we decided to establish a final data set containing only those genes associated with variations of expression of an amplitude ≥1.6x (Tables S1 and S2). This choice is aimed at minimizing the fraction of genes whose slight expression changes are only a consequence of the mild modification of the physiological state when the concentration of RNase Y decreases.

### Comparison With Similar Transcriptome Studies

The effect of RNase Y on global gene expression has been analysed previously in a microarray study performed under conditions of mild RNase Y depletion in a defined medium [23]. A second study using tiling arrays was carried out in parallel to this work under conditions of severe RNase Y depletion by growing the cells into stationary phase [11]. The growth conditions used here ressembled the Lehnik-Habrink *et al.* study [23] while the tiling arrays were the same as those used in the Durand *et al.* study [11].

Interestingly, and in contrast to what we observed in our study, plotting the evolution of the number of up- versus down-regulated genes when the amplitude cut-off is lowered (Fig. 2) revealed no break point in the slope when we used the data from the Lehnik-Habrink *et al.* and the Durand *et al.* studies (Fig. 2). Thus, this analysis provided no hint for the selection of a relevant cut-off to distinguish direct and indirect effects that were set respectively to 1.5x and 2x in Lehnik-Habrink *et al.* 2011 and Durand *et al.* 2012.

These differences are probably due to variations in the degree of RNase Y depletion between the three studies. Indeed the low amplitude of the effects described by Lehnik-Habrink *et al.* 2011 (Fig. 2) suggests that depletion was not as strong as in our work, whereas Durand *et al.* 2012 applied a deliberate strategy of extreme depletion. Presumably, the strategy applied by Lehnik-Habrink et al. 2011 minimizes indirect effects but hampers the ability to detect statistically significant differential expression. In contrast, the experimental set-up of Durand *et al.* 2012 could yield indirect effects that would be difficult to distinguish from direct effects in terms of amplitude.

Our final list of differentially expressed genes accounted for about 30% of all protein encoding genes in *B. subtilis* : within this list, 958 annotated transcripts exhibited an increased expression and 310 a decreased expression.

**Figure 3:**
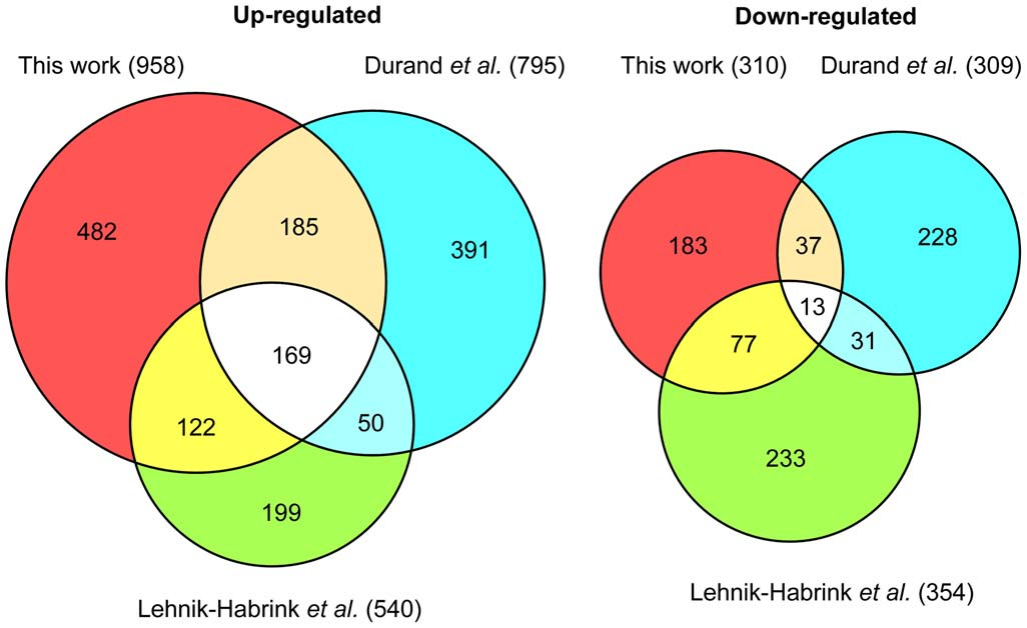
Effects of RNase Y depletion on the abundance of *B. subtilis* mRNAs. A comparison of three studies. The Venn diagrams show the numbers of mRNAs altered following RNase Y depletion and highlights the number of mRNAs common to two or all three studies. The color code is as follows : Red, this work. Blue, a tiling array study by Durand *et al.* (2012). Green, a classical transcriptome study by Lehnik-Habrink *et al.* (2011). The numbers in parentheses indicate the total number of mRNAs considered to be altered in each study.

The results obtained were surprisingly different from both previous studies. Only about one third of the upregulated transcripts identified here (958 mRNAs) were in common with one or the other study. Even more striking, out of 1598 mRNAs more abundant in the RNase Y mutant (corresponding to the cumulative non redundant total of the three studies) only 169 transcripts (10.5%) were in common in all three studies (Fig. 3). The equivalent values were even lower when we compared the downregulated transcripts, largely caused by indirect effects of RNase Y depletion. In that case, only 13 transcripts out of a non redundant total of 802 mRNAs were observed in all studies (Fig. 3).

### Direct Versus Indirect Effects On Mrna Abundance

Depletion of RNase Y can influence mRNA abundance by directly altering the stability of an individual transcript. However, if this transcript is for example a regulatory protein, indirect effects affecting the expression of the associated regulon would likely be observed. Typically, the strong downregulation of many competence genes that we observed in the RNase Y mutant was most likely due to the 4-fold reduced level of the *comK* mRNA, encoding the master regulator of the competence regulon (Table S2). We measured the half-lives of a few transcripts that were less abundant under conditions of RNase Y depletion and which a priori were not associated to a known regulon (e.g. *nhaX*, Table S2). We observed no direct effect of RNase Y on the stability of a downregulated transcript, i.e. a significant destabilization of a transcript in the RNase Y mutant (data not shown). This suggested that the decrease of most transcripts observed under our RNase Y depletion conditions is indirect but does by no means exclude that direct cleavage of a transcript by RNase Y can in certain cases lead to a more stable transcript.

In contrast, mRNA transcripts induced in the RNase Y mutant turned out to be direct substrates for the nuclease in all cases analysed. However, in one case we could show that induction of a non-coding RNA was indirect (see below). We observed no strong increase in the mRNA level of the major sigma factors and, in accordance, no increase of large numbers of transcripts belonging to the respective regulons.

### Individual Case Studies Of Annotated Gene Transcripts

We carried out a number of Northern blots to assess the level of confidence in the tiling array data and to analyse whether the differences in mRNA abundance are caused at the transcriptional and/or post-transcriptional level.

**Figure 4:**
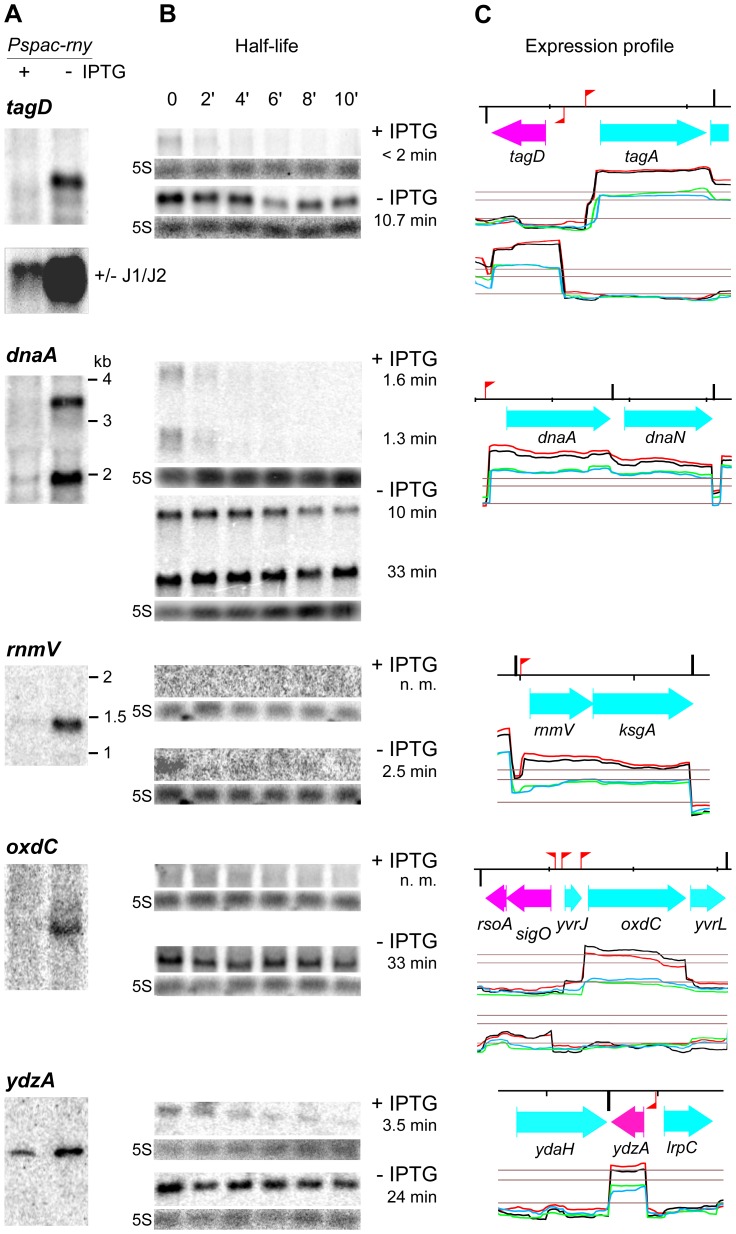
Transcription profiles and effect of RNase Y on the half-life of various mRNAs. A) Northern blot of total RNA isolated from the RNase Y depletion strain SSB447 (*Pspac-rny*) grown in the presence or absence of IPTG. The blots were probed with specific riboprobes complementary to 5′ proximal sequences of the genes *tagD*, *dnaA, rnmV*, *oxdC* and *ydzA*. (see Table S5). For *tagD,* the lower panel shows a Northern blot probed with the same probe but the RNA was isolated from a RNase J1 depletion strain where the gene *rnjB* encoding RNase J2 was deleted (reproduced from [10]). The blot shows that a slightly shorter species of the *tagD* mRNA accumulates in the absence of RNase J1/J2, probably by stabilizing an mRNA initially cut by RNase Y close to the 5′ end. B) Northern blot of total RNA isolated at times after addition of rifampicin from strain SSB447 grown in the presence or absence of IPTG. The time points indicated for the *tagD* gene were the same for all genes. The measured half-lives in min are indicated to the right. C) Close-up of the transcriptional landscape corresponding to the gene investigated, extracted from Fig. S1. The red flag poles and black vertical bars indicate sigma A type promoters and intrinsic transcription terminators, respectively, based on the annotation by Nicolas *et al.* [31]. The red/black and blue/green lines show independent duplicate transcription profiles from RNase Y depleted (-IPTG) and RNase Y induced cells (+IPTG). The lower horizontal line represents the global median over the whole chromosome and the two upper lines indicate 5x and 10x this value. The distance between the small markers on the top black line corresponds to 1 kb on the chromosomal DNA. Color codes for the annotation are as follows : cyan and magenta, protein coding sequences on the positive and negative strands, respectively. Black, new non coding RNA segment. Green, known non coding RNA.

The first example illustrates how RNase Y and RNase J can collaborate to degrade an RNA. In a previous combined transcriptome/proteome study we had identified transcripts that were upregulated in a *rnjA/rnjB* double mutant caused by an increase in mRNA stability [10]. One of them was the *tagD* mRNA encoding an essential enzyme for the biosynthesis of teichoic acid. However, the size of the *tagD* transcript that accumulated in the RNase J1/J2 mutant was slightly smaller than the full-length mRNA (Fig. 4A, reproduced from [10]). This had suggested that the mRNA was cleaved endonucleolytically by an unknown nuclease close to the 5′ end but not further degraded exonucleolytically from the 5′ end by RNase J. The *tagD* mRNA was strongly induced (5 to 10-fold) in a strain depleted for RNase Y (grown without IPTG) indicating that the initial cleavage close to the 5′ end of the *tagD* messenger can be mediated by RNase Y (Fig. 4A). In accordance, the increased abundance of the *tagD* messenger was clearly associated with an increased half-life of the transcript in the RNase Y mutant (10.7 min versus <2 min in the wild type strain, Fig. 4).

Interestingly, a reduced level of RNase Y in the cell strongly increases the expression of the entire *tagD* and the divergent *tagA* operon as well as the *tagO* gene, all of which are crucial for cell wall synthesis in *B. subtilis* (Fig. 4 and Fig. S1, Table S1). This suggests that RNase Y could also control the expression of a common regulator of cell wall synthesis (see discussion).

The transcript level for two other essential proteins, DnaA and DnaN, involved in the initiation of chromosomal replication is also directly dependent on the action of RNase Y. The two genes encoding the replication initiator protein (*dnaA*) and the β-subunit of DNA polymerase III (the beta clamp, *dnaN*) constitute an operon transcribed into mono- and bicistronic mRNAs (Fig. 4). Both transcript species are strongly increased in the RNase Y mutant and their half-lives increase from 1.3 to 10 min and 1.6 to 33 min, respectively (Fig. 4).

The observed deregulation of the *tag* and *dna* genes could be a cause for several effects on cell morphology observed during RNase Y depletion (see also Discussion).

Transcription of the major ribonucleases known in *B. subtilis* was not affected significantly in the RNase Y mutant. A notable exception was the *rnmV* gene encoding RNase M5, the 5S ribosomal RNA maturase. We found that the *rnmV* mRNA is co-transcribed with the *ksgA* gene encoding a 16S rRNA methyltransferase. The abundance of the 1.4 kb bicistronic mRNA was increased about 8-fold under RNase Y depletion. At the same time, the half-life of the transcript increased from not measureable to 2.5 min indicating that the effect of RNase Y on the *rnmV* transcript is direct (Fig. 4).

OxdC is an abundant manganese-dependent oxalate decarboxylase that accumulates in the cell wall of cells grown in acidic medium [26],[27]. The *oxdC* transcript was barely detectable in the presence of RNase Y but induced about seven-fold when the nuclease was depleted (Fig. 4). With a half-life of 33 min the induced *oxdC* transcript was very stable. The transcript appeared to be less stable under wild type conditions but due to the low level of the transcript in the presence of RNase Y we could not obtain a reliable measure of the *oxdC* mRNA half-life. *oxdC* is the major gene of the small *sigO* (*yvrI*) regulon [28],[29]. The *sigO* mRNA was also slightly induced in the RNase Y mutant, although remaining at a very low level (Fig. 4). Part of the increase in *oxdC* mRNA could thus be due to increased transcription.

Another clear example where RNase Y directly affects the abundance of a transcript is the *ydzA* mRNA (Fig. 4). It encodes a small predicted membrane protein of unknown function. The half-life of the *ydzA* mRNA increases about 7-fold from 3.5 to 24 min upon RNase Y depletion.

### Effects On The Expression Of Regulatory/antisense Rnas

Potential effects of RNase Y depletion should, in addition to mRNA transcripts, also lead to changes in abundance of regulatory and antisense RNAs, all of which can be detected by tiling arrays. In this category, we observed 399 RNAs that were more abundant/detectable following RNase Y depletion (Table S3) while 145 RNAs were reduced under these conditions (Table S4). Among them were 346 RNAs that were not seen in the Rasmussen and Irnov studies [22],[30]. However, practically all of them were also identified in a recent large study using the same tiling array setup as in this work but analysing expression profiles under more than one hundred different growth conditions [31]. We thus refer to all RNAs described here using the same « segment » (S) number as in the Nicolas *et al.* 2012 paper which corresponds to their order of appearance on the *B. subtilis* genome.

**Figure 5:**
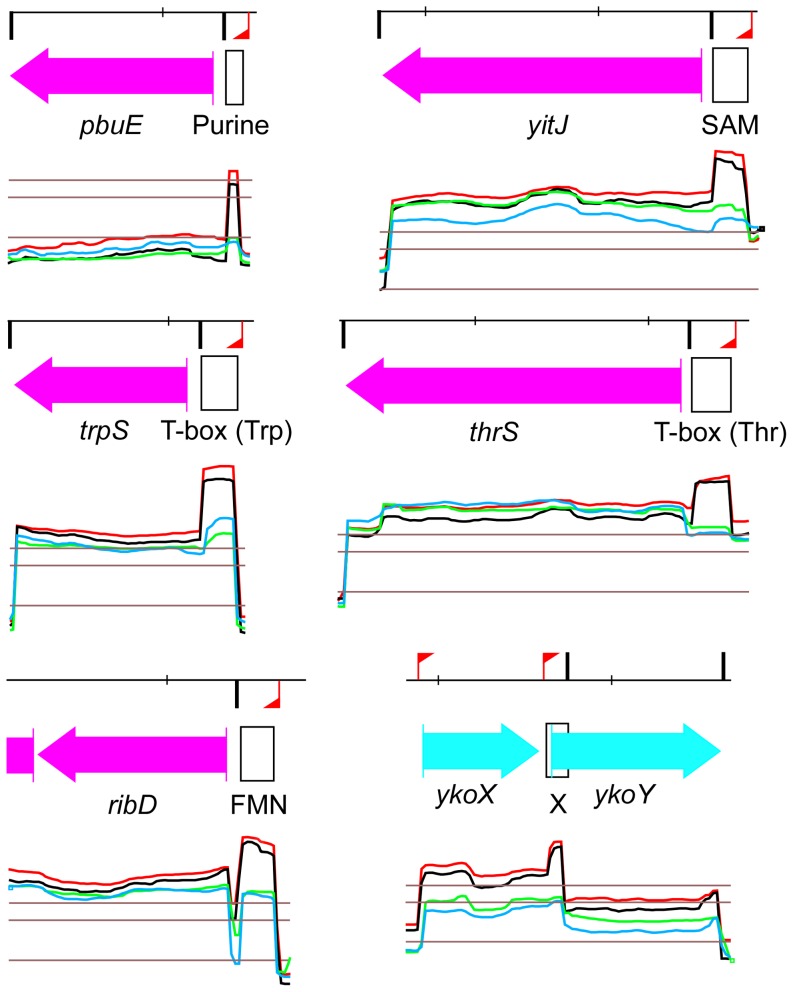
Riboswitch RNAs whose abundance is dependent on RNase Y expression. Transcriptional profiles of a selection of six riboswitches controlling the expression of the downstream gene via transcriptional attenuation are shown. In all cases the terminated leader transcript accumulates strongly when strain SSB447 (*Pspac-rny*) is grown in the absence of IPTG. The color codes are as described in Fig. 4C. The positions of the riboswitch RNAs extending to the leader terminator are indicated as black outlined boxes. Purine : adenine riboswitch. T-box (trp) : tryptophane dependent tRNA mediated antitermination. FMN : flavin mononucleotide-dependent riboswitch. SAM : S-adenosylmethionine dependent riboswitch, RNase Y effect experimentally shown in [12]. T-box (thr) : threonine dependent tRNA mediated antitermination. X : predicted riboswitch regulation [44].

The RNAs whose expression profile was altered were classified into six categories which include RNA genes and 5′ cis-acting structures (Tables S3 and S4, see legends explaining the meaning of the different categories). We identified 27 5′ cis-acting structures that were all upregulated (up to 10-fold) following RNase Y depletion. They include a wide variety of riboswitches, all of them involved in transcriptional regulations. Among them are five out of 11 SAM dependent riboswitches including the *yitJ* leader which has been shown to be cleaved by RNase Y *in vitro* and *in vivo* [12]. The list also contains half of all T-box directed antitermination systems (Table S3). A selection highlighting the role of RNase Y in the degradation of prematurely terminated leader transcripts in diverse antitermination (riboswitch) systems is shown in Figure 5.

**Figure 6:**
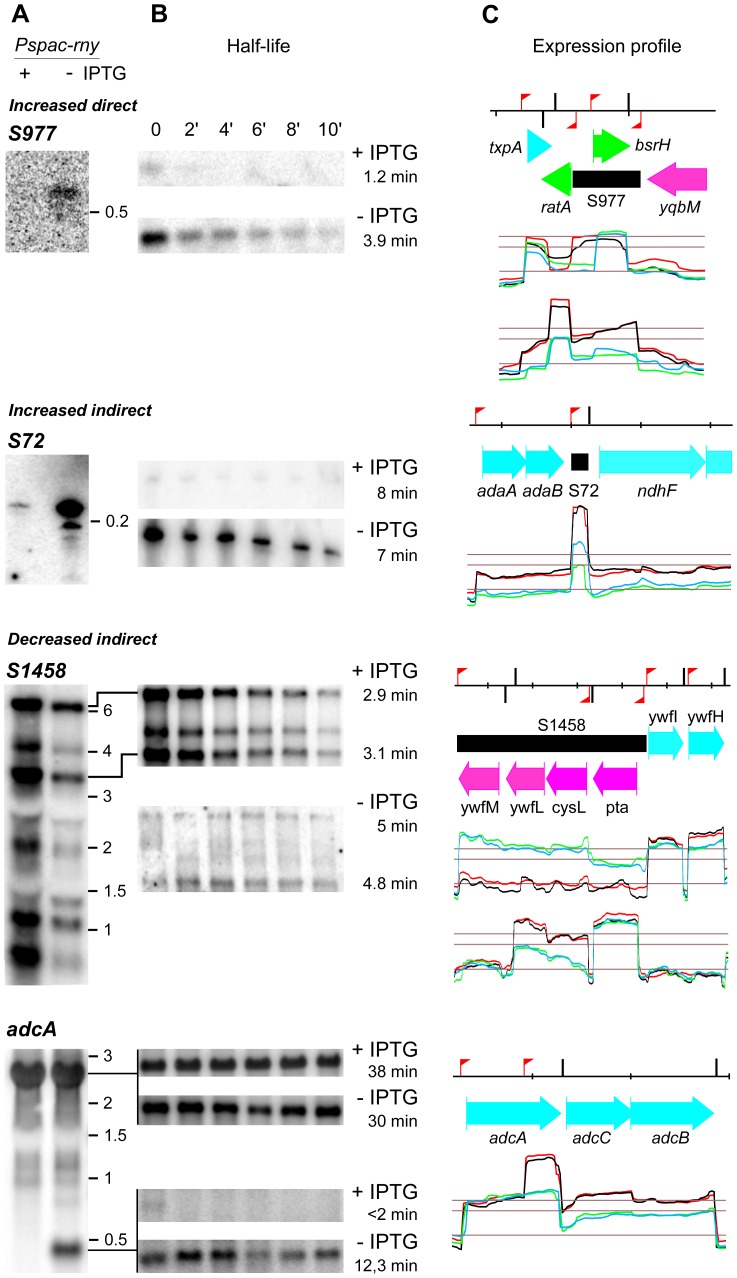
Transcription profiles and effect of RNase Y on the half-life of various non coding RNAs. A) Northern blot of total RNA isolated from the RNase Y depletion strain SSB447 (*Pspac-rny*) grown in the presence or absence of IPTG. The blots were probed with specific riboprobes complementary to 5′ proximal sequences of the respective DNA regions (see Table S5). B) Northern blot of total RNA isolated at times after addition of rifampicin from strain SSB447 grown in the presence or absence of IPTG. The measured half-lives in min are indicated to the right. C) Close-up of the transcriptional landscape corresponding to the gene investigated, extracted from Figure S1. The red flag poles and black vertical bars indicate sigma A type promoters and intrinsic transcription terminators, respectively, based on the annotation by Nicolas *et al.* (2012). The red/black and blue/green lines show independent duplicate transcription profiles from RNase Y depleted (-IPTG) and RNase Y induced cells (+IPTG). The lower horizontal line represents the global median over the whole chromosome and the two upper lines indicate 5x and 10x this value. The distance between the small markers on the top black line corresponds to 1 kb on the chromosomal DNA. Color codes for the annotation are as follows : cyan and magenta, protein coding sequences on the positive and negative strands, respectively. Black, new non coding RNA segment. Green, known non coding RNA.

We analysed several examples where RNase Y directly affects the abundance of a small non coding RNA in more detail. One of them is the S977 RNA that is barely detectable in the presence of RNase Y. When the nuclease is depleted, the S977 transcript, presumably transcribed from a Sigma A promoter, accumulates and shows a 3-fold increased half-life of 3.9 min compared to 1.2 min when RNase Y is normally expressed (Fig. 6). The transcript size of about 700 bases indicated that S977 is co-transcribed with *ratA* (Fig. 6) a small untranslated RNA antitoxin that blocks the accumulation of the mRNA for the toxic peptide TxpA [32]. The *ratA* RNA can be transcribed from a dedicated Sigma A promoter [32]) and this small RNA of about 200 bases is also strongly induced in the RNase Y mutant (Table S3, S976). Overexpression of the S977 RNA which is antisense to the previously identified non coding RNA *bsrH* (Fig. 6) does not significantly affect the abundance of the latter (see upper profile in Fig. 6). However, the *bsrH* RNA might also be part of a longer transcript that only accumulates during RNase Y depletion (Fig. 6).

All mRNA transcripts tested whose abundances were increased in the RNase Y mutant were directly affected in their stability (Fig. 4). The small 200 base S72 non coding RNA is an example of how RNase Y depletion can increase the abundance of a transcript indirectly (without changing RNA stability), probably by increasing the transcription of the RNA gene (Fig. 6).

The long non coding S1458 RNA is transcribed from a sigma A type promoter producing at least 6 RNA species ranging in length from about 500 bases to 6 kb (Fig. 6). The S1458 RNA has a length of 4 kb, the longest 6 kb transcript detected in the Northern analysis thus includes the two downstream mRNAs *ywfI* and *ywfH*. The two major species of 3.5 and 6 kb are stabilized in the absence of RNase Y. However, under these conditions S1458 transcription is strongly reduced due to an indirect effect, leading to an overall decrease of the transcript level. The S1458 transcript is an antisense RNA with respect to four genes transcribed from the opposite strand (Fig. 6). Its decrease in the RNase Y mutant is accompanied by a strong increase in the level of the *ywfL* (*lipL*) and *cysL* mRNAs, an amidotransferase involved in lipoic acid biosynthesis [33] and a transcriptional repressor of sulfur assimilation [34].

An interesting case of a previously not described non coding RNA is located within the *adcA* (*ycdH*) gene encoding a zinc-binding lipoprotein which, together with the products of the two downstream genes *adcC* (*ycdI*) and *adcB* (*yceA*), constitutes an ABC transporter responsible for zinc uptake [35],[36]. The non coding RNA actually corresponds to the 400 3′ proximal nucleotides of the *adcA* mRNA transcribed from an internal sigma A promoter identified in [31]. Under our conditions, this RNA is only detected when RNase Y is depleted (Fig. 6). The effect of RNase Y on the abundance of this small RNA is direct as its half-life increases from less than 2 min to over 12 min upon RNase Y depletion. At the same time neither the abundance nor the stability of the major 2.5 kb tricistronic *adcA-adcC-adcB* mRNA is significantly altered (Fig. 6). This pattern is consistent with a direct cleavage by RNase Y within the 3′ 400 nucleotides of the *adcA* mRNA that only occurs when this region is not translated, i.e. when transcribed from the internal promoter.

## Discussion

Evidence that endonucleolytic cleavage plays a major role in mRNA metabolism in *B. subtilis* came from the initial characterization of RNase Y which showed a strong effect on global mRNA stability in strains depleted for this enzyme [12]. The data described here are clearly consistent with a key role for RNase Y in initiating mRNA decay through endonucleolytic cleavage similar to the RNase E dependent *E. coli* model of mRNA degradation. The expression of almost one third of the genome was affected by RNase Y depletion, a number that increases to almost 60% if we integrate the results obtained by similar studies (see below). Nevertheless, the involvement of RNAse Y in mRNA turnover is likely to be even more important if one considers that depletion of the nuclease (6-fold in the present study) is not equivalent to a knock-out strain. The remaining RNase molecules might well be sufficient to cleave very high affinity substrates. In addition, about 90 transcripts were expressed close to saturation levels in the presence of RNase Y so that a further increase in mRNA in the depletion strain would become undetectable. This likely explains why for example our measurements for *rpsO* mRNA, a known RNase Y substrate [19] indicated only a 1.4-fold accumulation, just below the applied treshold of 1.6x.

However, other previously identified substrates of RNase Y show up in our global analysis. This is the case for five SAM riboswitch leaders including the *yitJ* gene [12] which accumulated up to 6-fold in the depleted strain (Table S3, Fig. 4). In addition, many of the T-box leaders accumulated under these conditions. Interestingly, some of them (e.g. the *thrS* and *thrZ* leaders can also be a substrate for the endonucleolytic activity of RNases J1/J2 [5]. Indeed, RNase J and RNase Y and to that matter also *E. coli* RNase E have very similar endonucleolytic cleavage specificities and the *thrS* T-box leader can actually be cut *in vitro* at the same position by all three nucleases [17]. The respective contributions of RNases J and Y to T-box leader cleavage *in vivo* remains to be analysed but the strong and broad effect of RNase Y observed here suggests that this enzyme plays a major role in the turnover of T-box leader RNAs and riboswitch RNAs in general in *B. subtilis.* Indeed, the 27 riboswitch RNAs (all of which act on the transcriptional level) with increased abundance under RNase Y depletion represent 29% of all 5′ *cis* acting structures known in *B. subtilis* (92 RNAs, as referenced in Table S6.6 of [31]). In addition, the effect of RNase Y on the *infC* leader containing an L20-dependent transcriptional attenuator [20] is also accurately reproduced (S1089 in Table S3).

An increase in the abundance of a given transcript following RNase Y depletion was generally caused by a direct effect of the nuclease, i.e. reduced cleavage of the transcript leading to an increase in mRNA stability. The mRNAs of two sigma factors SigI and SigM were significantly increased, 3.7- and 3.5-fold, respectively. However, their regulons are not well defined. Among 14 genes reported as possible members of the SigI regulon [31], six mRNAs (*ftsW*, *ypuD, yvyE, bcrC* and *murAA*) where more abundant under RNase Y depletion. Whether their induction is due to an increase of SigmaI directed transcription rather than a direct effect of RNase Y on their stability remains to be confirmed. The only case where we have experimentally shown that an increase in RNA abundance following RNase Y depletion was indirect concerned a small non coding RNA gene. Transcription of the S72 ncRNA presumably from a dedicated sigma A type promoter is strongly induced in the RNase Y mutant without affecting the stability of the RNA. The S72 ncRNA could also be co-transcribed with the upstream *adaAB* operon involved in the alkylative DNA damage response whose mRNA is also induced in the RNase Y mutant. However, in that case the S72 RNA must be processed very efficiently to its final size as we detected no longer transcripts in the Northern analysis.

Decreased abundance of a given transcript following RNase Y depletion is likely due to indirect effects. Despite our efforts to keep any growth defect following nuclease depletion to a minimum, a minor slow down in growth rate still occurred and actually determined the point where RNase Y depleted cells were harvested. This likely explains the reduction in the mRNA levels encoding ribosomal proteins and amino acid biosynthetic operons both of which are very sensitive to growth variations (Table S2). Nevertheless, direct RNase Y targets can also be found among RNAs with decreased abundance. The S1458 antisense RNA is stabilized in the RNase Y mutant but strongly reduced due to an overriding transcriptional effect. The reduction of the S1458 antisens RNA could be directly responsible for the concomitant increase in the *cysL-ywfL* mRNA transcribed from the opposite strand but we cannot exclude that this mRNA is directly cleaved by RNase Y.

The increase in abundance of a 3′ portion of an mRNA following RNase Y depletion is a rather rare event and we observed this phenomenon in only a few cases on the entire transcriptome. The 400 3′-proximal nucleotides of the *adcA* mRNA is an example of RNA that strongly accumulates under these conditions. In this case, this is accompanied by a parallel increase of the transcript levels of the downstream *adcA* and *adcB* genes, which together with *adcA* encode a zinc ABC transporter (Fig. 6). This is most likely due to a higher stability of the transcript originating at the *adcA* internal promoter in absence of RNase Y. In fact, the 3′-terminal region of *adcA* is probably not translated when transcribed from the internal promoter and only susceptible to cleavage by RNase Y under these conditions. This would explain why the level of the functional *adcA* mRNA remains unchanged in the presence or absence of RNase Y. By contrast, the *adcC-adcB* transcript originating at the internal *adcA* promoter would contain an RNase Y cleavage site (the untranslated *adcA* 3′ region), hence its level is increased in the absence of the nuclease.

The comparison of the transcriptome data obtained here with those of the two recent studies mentionned above has revealed very significant differences. The exceptionally low concordance of downregulated mRNAs following RNase Y depletion (13 out of a non redundant total of 802 mRNAs) between the three studies is in agreement with an essentially indirect effect of RNase Y deficiency on the level of these transcripts. It is for example not surprising to see very little overlap in downregulated genes when comparing our experimental conditions designed to minimize growth effects with the Durand *et al.* study where RNase depletion was maximized by growing the cells into stationary phase.

The weak overlap in induced mRNAs between the three studies which are considered in the large majority to be caused by a direct effect of RNase Y are more surprising. For example, more than half of the mRNAs with increased abundance under RNase Y depletion identified here (958 transcripts) are not found in the Lehnik-Habrink and Durand studies. Actually only about 10% of all induced mRNA transcripts are in common in the three studies. Curiously, the expression of the majority of genes from prophage PBSX are increased following RNase Y depletion in our study and that of Durand *et al.* but decreased in the Lehnik-Habrink study. However, the culture conditions were much more similar between this study and that of Lehnik-Habrink. We also compared the results of the three studies using a general 1.5x cut-off without considering any statistical evaluation (there was no statistical evaluation carried out in the Lehnik-Habrink study). While the number of transcripts affected obviously increased, the overlap in induced or reduced mRNAs following RNase Y depletion between the three studies was not improved (Fig. S3).

This implies that the experimental conditions are extremely important for the outcome of the experiment. Differences in medium, growth conditions, degree of RNase depletion and statistical data evaluation are all critical. A single experimental condition does obviously not permit to identify all or even a majority of the major RNase Y substrates. Most obvious is the fact that RNase Y depletion can impact directly (via increased half-life) only those genes that are expressed. However, detection of differential expression for highly expressed genes might also be difficult due to technical limitations in the dynamic range of the microarray measurements. To some extent it is also likely that the most highly expressed genes in the reference condition are more prone to be subject to downward indirect effects – a pattern of ‘regression towards the mean’ - that could mask upward direct effects. As a probable consequence, our ability to detect upward effects of RNase Y depletion reaches a maximum in the central part of the expression level spectrum (Fig. S2) where as much as ∼35% of the genes are seen up-regulated versus ∼7% seen down-regulated. It can thus be reasoned that if the indirect effects cause the same amount of up- and down-regulation and if all down-ward effects are indirect, then indirect effects contribute to ∼20% (7/35) of the up-regulated genes and thus genes directly affected by RNase Y depletion would account for ∼28% (35–7) of the total gene repertoire according to the degree of RNase Y depletion and the amplitude cut-off selected in our study. This projection is aimed to account for limitations due to the dynamic range of our measurements and the fact that only one reference condition was considered. Still, it almost certainly underestimates the number of RNase Y targets as our mild depletion set-up (measured to 1/6th of its original concentration) might not reveal the targets with the highest affinity: this could explain some or most of the differences between our results and those of Durand *et al.* (2012). Indeed, addition of the list of differentially expressed genes found in our study and in Durand *et al.* study increases by ∼46% the number of potential RNase Y targets.

The global effect of this nuclease does not allow to draw any meaningful conclusions as to why RNase Y has such an important effect on growth. An earlier study has shown that depletion of YmdA (RNase Y) resulted in several effects on cell and chromosome morphology [13]. Potentially related to these observations, the essential *tagD* and *dnaA* transcripts were strongly increased following RNase Y depletion (Fig. 4). The *dnaA* gene encodes the replication initiation protein and its overexpression has been linked to aberrant changes in cell shape [37]. The *tagD* gene belongs to the teichoic acid biosynthetic apparatus of which other components like the *tagA* mRNA are also increased. We have shown that RNase Y affects *tagD* mRNA stability directly but the more general effect of RNase Y on the expression of teichoic acid biosynthetic genes suggested that the nuclease could also control the expression of a common regulator. It is noteworthy that the expression of the two-component signal transduction systems *walRK* and *phoPR* implicated in peptidoglycan and teichoic acid metabolism (for reviews, see [38],[39]) is significantly increased (3-fold) under RNase Y depletion conditions. This is also the case for example for some genes activated by WalR, like *ydjM* and the autolysine *lytE* (see Table S1).

We observed no significant effect of RNase Y on the transcript level of any known ribonuclease with the exception of *rnhC* and *rnmV* encoding the RNA-DNA hybrid specific RNase HIII and the 5S rRNA maturase RNase M5, respectively. Notably, no cross-regulation occurs between RNase Y and RNases J1/J2. As shown previously, depletion of RNases J1 and J2 alone or together has no influence on the level of the *rny* (*ymdA*) transcript [10],[11].

We performed a statistical analysis of functional categories of mRNAs affected in the RNase Y mutant. We focused on upregulated RNAs which in their large majority are direct substrates for RNase Y. 114 functional categories and sub-categories were examined (Table S6). A summary of over- or under-represented functional categories is shown in Table 1. RNase Y appears to have an important general role in the synthesis of components involved in DNA replication, iron metabolism and the cell envelope and cell wall. In addition, most of the mRNAs encoded by the prophage PBSX are increased under RNase Y depletion conditions. However, it remains to be determined whether the effect of RNase Y on a given functional category is significant.

**Table 1:** Functional categories of mRNAs more abundant under conditions of RNase Y depletion.

Functional category	Number genes	RNase Yn° up	RNase Y% up
Reference for whole genome	4244	958	22,6
Cellular processes * cell envelope and cell division * cell division	25	9	36,0
Cellular processes * cell envelope and cell division * cell wall synthesis	74	26	35,1
Cellular processes * homeostasis * acquisition of iron	41	19	46,3
Information processing * genetics * DNA replication	24	11	45,8
Information processing * regulation of gene expression * transcription factors and their control	322	103	32,0
Information processing * RNA synthesis and degradation * transcription	33	11	33,3
Lifestyles * coping with stress * heat shock proteins	27	10	37,0
Metabolism * additional metabolic pathways * biosynthesis of cell wall components	56	22	39,3
Metabolism * additional metabolic pathways * iron metabolism	52	19	36,5
Prophages and mobile genetic elements * prophages * PBSX prophage	38	25	65,8
Cellular processes * transporters * phosphotransferase systems	28	2	7,1
Information processing * protein synthesis, modification and degradation * translation	295	16	5,4
Lifestyles * coping with stress * biosynthesis of antibacterial compounds	52	4	7,7
Lifestyles * coping with stress * general stress proteins (controlled by SigB)	151	10	6,6
Lifestyles * exponential and early post-exponential lifestyles * motility and chemotaxis	68	2	2,9
Metabolism * electron transport and ATP synthesis * respiration	37	2	5,4
Prophages and mobile genetic elements * prophages * SP-beta prophage	186	14	7,5

Different levels of functional categories are separated by asterisks.

doi:10.1371/journal.pone.0054062.t001

This study has more than doubled the number of coding and non coding RNAs whose abundance is increased under RNase Y depletion. The large majority of these RNAs are likely to be direct targets of RNase Y and make this nuclease a key player in the initiation of RNA decay in *B. subtilis*, confirming its previously shown strong effect on global mRNA stability. The precision of the whole-genome transcriptional landscapes (Fig. S1) allows a very detailed view of the effect of RNase Y on any RNA transcript.

## Materials And Methods

### Bacterial Strains

The *B. subtilis* strain used in this study is SSB447 a derivative of strain 1A2 (BGSC). In SSB447 the *rny* gene is under control of the IPTG inducible *Pspac* promoter [12]. The strain harbors plasmid pMAP65 [25] overexpressing the *lacI* repressor allowing more efficient depletion of RNase Y in the absence of inducer.

### Growth Conditions

*B. subtilis* RNase Y depletion strain SSB447 was grown in SMS minimal medium [40] supplemented with 0.75% fructose, 0.1% L-glutamine and 2 mM IPTG. Antibiotics were added at the following concentrations : 0.25 µg/ml erythromycin, 12.5 µg/ml lincomycin (*mls* resistance) and 5 µg/ml kanamycin.

### Tiling Array Analysis

Strain SSB447 grown to mid-log phase in LB medium in the presence of 2 mM IPTG was rediluted in SMS medium with IPTG (see above) at an OD_600_ of 0.0015 and incubated overnight at 30°C. Cultures typically are in exponential growth phase the next morning. Cells were washed in SMS medium without IPTG and inoculated in fresh SMS medium at an OD_600_ of 0.05, with or without IPTG. The cultures were grown at 37°C and cell density was monitored every 20 min. When a first slow-down in growth became measurable, typically at an OD_600_ of 0.5, cells were harvested and RNA was prepared using a MiKro-Dismembrator S (Sartorius) as described [31]. RNA concentrations were measured and sent to Roche/Nimblegen for labeling and analysis on second-generation (T2) tiling arrays according to the BaSysBio protocol [31].

### Data Analysis

The processing of the raw hybridization data to obtain gene-level expression measures normalized between experiments was done as described previously [11]. Briefly, the first step consisted in reconstructing expression profiles along the chromosome [41]; Figure S1), expression values were then aggregated at the gene-level by taking the median of the estimated probe-level values as in [31]. Finally the normalization method described in [11] based on the least variant set of gene was applied to reduce the impact of technical variations between hybridizations with mimimum impact on the un-balance between up- and down- effects of RNase Y depletion.

We applied a t-test to the expression values collected for each gene to identify those whose expression level depends on the presence/absence of IPTG. Then, in order to decide whether the null hypothesis of absence of differential expression could be rejected for a particular gene in our multiple testing set-up, we converted the p-values into estimates of the local False Discovery Rate (FDR) using the method implemented in the R library “fdrtool” [42].

In addition to annotated genes, we included in our analysis unannotated transcribed segments mapped in references [31] and [11], respectively denoted «S» and «T» in Tables S3 and S4.

### Northern Blot Analysis

Northern blot analysis was carried out as described [43] using 5 µg of total RNA separated either on a 1.2% agarose or 8% acrylamide gel. Continously α-[^[32]^P] UTP-labelled riboprobes complementary to 5′ proximal sequences of the respective genes were generated by T7 polymerase *in vitro* transcription of specific PCR fragments made with the primers listed in Table S5.

For mRNA half-life determination, Rifampicin (150 µg/ml) was added to exponentially growing *B. subtilis* cells to prevent transcription initiation. Samples for RNA preparation were removed before and at different times after rifampicin addition. These RNA samples were hybridized in Northern experiments with gene specific RNA probes. mRNA half-lifes were calculated from the slopes of the individual decay curves.

### Western Blot Analysis

For Western blot analysis, 10 µg of protein extract was separated by one-dimensional SDS-PAGE (12.5%). After electrophoretic transfer of the proteins, the nitrocellulose membrane was blocked with 5% skimmed milk powder in Tris/NaCl buffer (0.5 M Tris/HCl, pH 7.6, 0.15 M NaCl). Subsequently, the membrane was incubated with polyclonal anti-RNase Y antibodies. After washing, alkaline phosphatase-conjugated anti-rabbit IgG (Dianova, Hamburg, Germany) was added, and the blot was developed with fluorescent ECF substrate (GE Healthcare). The blot was scanned using a Typhoon PhosphoImager (Molecular Dynamics Sunnyvale, California).

Quantification of the bands was carried out with the ImageQuant software (Molecular Dynamics).

## Supporting Information

Figure S1 **Genome-wide transcriptional landscapes of RNase Y depleted cells.** The red/black and blue/green lines show independent duplicate transcription profiles from RNase Y depleted (-IPTG) and RNase Y induced cells (+IPTG). The lower horizontal line represents the global median over the whole chromosome and the two upper lines indicate 5x and 10x this value. Additional information reported here above the transcriptional landscape is from top to bottom: the intrinsic terminators predicted by PETRIN (vertical bars along the x-axis), the genbank annotation (AL009126.3), the new transcription segments and the promoters and terminators identified in Nicolas et al. (2012).
(PDF)

Figure S2 **Detection of regulation by RNase Y is affected by the expression level in the reference samples.** The black line shows the distribution of the expression level for the total repertoire of protein coding genes. The green, red and gray lines correspond respectively to the distribution of expression level for the three sub-categories: up-regulated, down-regulated and other genes. The fraction of up-regulated genes varies as a function of the gene expression level in the reference and reaches a maximum near 10.5, where up-regulated genes account for ∼35% of the total. At the same point down-regulated genes account for ∼7% of the total. Probability density functions were estimated by a kernel method implemented in function ‘density’ of R with bandwidth 0.5. Density functions for the three sub-categories (up-regulated genes, down-regulated genes, other genes) are scaled to be proportional to the fraction of genes in each category.
(PDF)

Figure S3 **Effects of RNase Y depletion on the abundance of** ***B. subtilis*** **mRNAs.** A comparison of three studies where (in contrast to Figure 3) a common arbitrary cut-off value of 1.5x has been applied. The Venn diagrams show the numbers of mRNAs altered following RNase Y depletion and highlights the number of mRNAs common to two or all three studies. The color code is as follows : Red, this work. Blue, a tiling array study by Durand *et al.* (2012). Green, a classical transcriptome study by Lehnik-Habrink *et al.* (2011). The numbers in parentheses indicate the total number of mRNAs considered to be altered in each study.
(TIF)

Table S1 **Fold changes (>1.6x) in annotated genes whose mRNAs are more abundant under RNase Y depletion.** The genes are listed in decreasing order with respect to fold increases in mRNA following depletion of RNase Y compared to a strain expressing RNase Y wild type levels. The last three columns compare the fold increase in expression levels of each gene identified here with those observed in the Lehnik-Habrink (2011) and Durand (2012) studies, respectively. The conclusions drawn for the differential expression of each gene in the respective studies is shown in the last column (U = up, D = down, − = not modified).
(XLSX)

Table S2 **Fold changes (>1.6x) in annotated genes whose mRNAs are less abundant under RNase Y depletion.** The genes are listed in decreasing order with respect to fold decrease in mRNA following depletion of RNase Y compared to a strain expressing RNase Y wild type levels. The last three columns compare the fold decrease in expression levels of each gene identified here with those observed in the Lehnik-Habrink (2011) and Durand (2012) studies, respectively. The conclusions drawn for the differential expression of each gene in the respective studies is shown in the last column (U = up, D = down, − = not modified).
(XLSX)

Table S3 **Fold changes (>1.6x) in regulatory/antisens RNAs that are more abundant under RNase Y depletion.** The RNAs whose expression profile was altered were classified into six categories, all of them described in Nicolas et al., 2012, [31], see Supplementary Online Material, especially the SOM 5 section from 1206848.Nicolas.SOM.pdf, and Table S6.xlsx) : 1) Known RNA genes identified individually by 5′ end mapping and Northern blot analysis, reported in the literature. 2) New RNA genes, so called independent transcription units identified through the large scale tiling array analysis described in [31]; 3) New CDSs, unannotated expressed regions described in [31] and containing a coding sequence detected with high confidence by SHOW, based on an enriched HMM model of DNA sequences [45]; 4) 5′ cis-acting structures compiled from [30], and reliable predictions against the RFAM database [46], reported in the Genome Reviews version of annotated genomes from the EBI [47]; 5) Newly expressed antisense segments, complementary to expressed regions corresponding to 5′ UTRs, 3′ UTRs, and intergenic regions of annotated features; 6) Newly expressed segments not antisense, classified as in the previous item, but not antisense, see [31] for criteria distinguishing antisense regions from those that are not. We refer to all RNAs described here using the same «segment» (S) number and classification as in [31] which corresponds to their order of appearance on the B. subtilis genome. RNAs that were also seen in the Rasmussen and Irnov studies [22],[30] are indicated. The two last columns compare the fold effect in expression levels of each RNA identified here with those observed in the Durand et al. [31] study. The conclusions drawn for the differential expression of each RNA in the respective studies is shown in the last column (U = up, D = down, − = not modified).
(XLSX)

Table S4 **Fold changes (>1.6x) in regulatory/antisens RNAs that are less abundant under RNase Y depletion.** See legend of Table S3 for explanations.
(XLSX)

Table S5 **Oligonucleotides used in this study.** Hybridizing sequences in first PCR cycle are in upper case; non-hybridizing sequences are lower case.
(DOCX)

Table S6 **Functional categories of genes showing increased expression following depletion of RNase Y.** Functional categories are from Subtiwiki v20120508 (http://subtiwiki.uni-goettingen.de/wiki/index.php/Categories). A selection of categories with numbers of increased mRNAs that are significantly above (green) or below (orange) the whole genome reference value of 22.6% (yellow) are indicated.
(XLS)

## References

[1] Carpousis AJ, Luisi BF, McDowall KJ (2009) Endonucleolytic initiation of mRNA decay in Escherichia coli. Prog Mol Biol Transl Sci 85: 91–135. 19215771 10.1016/S0079-6603(08)00803-9

[2] Garrey SM, Mackie GA (2011) Roles of the 5′-phosphate sensor domain in RNase E. Mol Microbiol. 80: 1613–1624. 10.1111/j.1365-2958.2011.07670.x 21518390

[3] Kime L, Jourdan SS, Stead JA, Hidalgo-Sastre A, McDowall KJ (2010) Rapid cleavage of RNA by RNase E in the absence of 5′ monophosphate stimulation. Mol Microbiol 76: 590–604. 19889093 10.1111/j.1365-2958.2009.06935.x PMC2948425

[4] Deana A, Celesnik H, Belasco JG (2008) The bacterial enzyme RppH triggers messenger RNA degradation by 5′ pyrophosphate removal. Nature 451: 355–358. 18202662 10.1038/nature06475

[5] Even S, Pellegrini O, Zig L, Labas V, Vinh J, et al (2005) Ribonucleases J1 and J2: two novel endoribonucleases in *B. subtilis* with functional homology to *E. coli* RNase E. Nucleic Acids Res. 33: 2141–2152. 10.1093/nar/gki505 PMC1079966 15831787

[6] Mathy N, Benard L, Pellegrini O, Daou R, Wen T, et al (2007) 5′-to-3′ Exoribonuclease Activity in Bacteria: Role of RNase J1 in rRNA Maturation and 5′ Stability of mRNA. Cell 129: 681–692. 17512403 10.1016/j.cell.2007.02.051

[7] Li de la Sierra-Gallay I, Zig L, Jamalli A, Putzer H (2008) Structural insights into the dual activity of RNase J. Nat Struct Mol Biol. 15: 206–212. 10.1038/nsmb.1376 18204464

[8] Richards J, Liu Q, Pellegrini O, Celesnik H, Yao S, et al (2011) An RNA pyrophosphohydrolase triggers 5′-exonucleolytic degradation of mRNA in Bacillus subtilis. Mol Cell 43: 940–949. 21925382 10.1016/j.molcel.2011.07.023 PMC3176438

[9] Mathy N, Hebert A, Mervelet P, Benard L, Dorleans A, et al (2010) Bacillus subtilis ribonucleases J1 and J2 form a complex with altered enzyme behaviour. Mol Microbiol 75: 489–498. 20025672 10.1111/j.1365-2958.2009.07004.x

[10] Mäder U, Zig L, Kretschmer J, Homuth G, Putzer H (2008) mRNA processing by RNases J1 and J2 affects *Bacillus subtilis* gene expression on a global scale. Mol Microbiol 70: 183–196. 18713320 10.1111/j.1365-2958.2008.06400.x

[11] Durand S, Gilet L, Bessieres P, Nicolas P, Condon C (2012) Three Essential Ribonucleases-RNase Y, J1, and III-Control the Abundance of a Majority of Bacillus subtilis mRNAs. PLoS Genet 8: e1002520. 22412379 10.1371/journal.pgen.1002520 PMC3297567

[12] Shahbabian K, Jamalli A, Zig L, Putzer H (2009) RNase Y, a novel endoribonuclease, initiates riboswitch turnover in Bacillus subtilis. EMBO J 28: 3523–3533. 19779461 10.1038/emboj.2009.283 PMC2782095

[13] Hunt A, Rawlins JP, Thomaides HB, Errington J (2006) Functional analysis of 11 putative essential genes in *Bacillus subtilis* . Microbiology 152: 2895–2907. 17005971 10.1099/mic.0.29152-0

[14] Commichau FM, Rothe FM, Herzberg C, Wagner E, Hellwig D, et al (2009) Novel activities of glycolytic enzymes in *Bacillus subtilis*: interactions with essential proteins involved in mRNA processing. Mol Cell Proteomics 8: 1350–1360. 19193632 10.1074/mcp.M800546-MCP200 PMC2690492

[15] Lehnik-Habrink M, Newman J, Rothe FM, Solovyova AS, Rodrigues C, et al (2011) RNase Y in Bacillus subtilis: a Natively disordered protein that is the functional equivalent of RNase E from Escherichia coli. J Bacteriol 193: 5431–5441. 21803996 10.1128/JB.05500-11 PMC3187381

[16] Newman JA, Hewitt L, Rodrigues C, Solovyova AS, Harwood CR, et al (2012) Dissection of the network of interactions that links RNA processing with glycolysis in the Bacillus subtilis degradosome. J Mol Biol 416: 121–136. 22198292 10.1016/j.jmb.2011.12.024

[17] Laalami S, Putzer H (2011) mRNA degradation and maturation in prokaryotes: the global players. Biomolecular Concepts 2: 491–506. 25962050 10.1515/BMC.2011.042

[18] Naville M, Gautheret D (2010) Premature terminator analysis sheds light on a hidden world of bacterial transcriptional attenuation. Genome Biol 11: R97. 20920266 10.1186/gb-2010-11-9-r97 PMC2965389

[19] Yao S, Bechhofer DH (2010) Initiation of decay of Bacillus subtilis rpsO mRNA by endoribonuclease RNase Y. J Bacteriol. 192: 3279–3286. 10.1128/JB.00230-10 PMC2897663 20418391

[20] Bruscella P, Shahbabian K, Laalami S, Putzer H (2011) RNase Y is responsible for uncoupling the expression of translation factor IF3 from that of the ribosomal proteins L35 and L20 in Bacillus subtilis. Mol Microbiol 81: 1526–1541. 21843271 10.1111/j.1365-2958.2011.07793.x

[21] Marincola G, Schafer T, Behler J, Bernhardt J, Ohlsen K, et al (2012) RNase Y of Staphylococcus aureus and its role in the activation of virulence genes. Mol Microbiol 85: 817–832. 22780584 10.1111/j.1365-2958.2012.08144.x

[22] Rasmussen S, Nielsen HB, Jarmer H (2009) The transcriptionally active regions in the genome of Bacillus subtilis. Mol Microbiol 73: 1043–1057. 19682248 10.1111/j.1365-2958.2009.06830.x PMC2784878

[23] Lehnik-Habrink M, Schaffer M, Mader U, Diethmaier C, Herzberg C, et al (2011) RNA processing in Bacillus subtilis: identification of targets of the essential RNase Y. Mol Microbiol. 81: 1459–1473. 10.1111/j.1365-2958.2011.07777.x 21815947

[24] Diethmaier C, Pietack N, Gunka K, Wrede C, Lehnik-Habrink M, et al (2011) A novel factor controlling bistability in Bacillus subtilis: the YmdB protein affects flagellin expression and biofilm formation. J Bacteriol 193: 5997–6007. 21856853 10.1128/JB.05360-11 PMC3194898

[25] Petit MA, Dervyn E, Rose M, Entian KD, McGovern S, et al (1998) PcrA is an essential DNA helicase of Bacillus subtilis fulfilling functions both in repair and rolling-circle replication. Mol Microbiol 29: 261–273. 9701819 10.1046/j.1365-2958.1998.00927.x

[26] Antelmann H, Towe S, Albrecht D, Hecker M (2007) The phosphorus source phytate changes the composition of the cell wall proteome in Bacillus subtilis. J Proteome Res 6: 897–903. 17269748 10.1021/pr060440a

[27] Tanner A, Bowater L, Fairhurst SA, Bornemann S (2001) Oxalate decarboxylase requires manganese and dioxygen for activity. Overexpression and characterization of Bacillus subtilis YvrK and YoaN. J Biol Chem 276: 43627–43634. 11546787 10.1074/jbc.M107202200

[28] MacLellan SR, Helmann JD, Antelmann H (2009) The YvrI alternative sigma factor is essential for acid stress induction of oxalate decarboxylase in Bacillus subtilis. J Bacteriol 191: 931–939. 19047353 10.1128/JB.01435-08 PMC2632099

[29] MacLellan SR, Wecke T, Helmann JD (2008) A previously unidentified sigma factor and two accessory proteins regulate oxalate decarboxylase expression in Bacillus subtilis. Mol Microbiol 69: 954–967. 18573182 10.1111/j.1365-2958.2008.06331.x PMC2688449

[30] Irnov I, Sharma CM, Vogel J, Winkler WC (2010) Identification of regulatory RNAs in Bacillus subtilis. Nucleic Acids Res 38: 6637–6651. 20525796 10.1093/nar/gkq454 PMC2965217

[31] Nicolas P, Mader U, Dervyn E, Rochat T, Leduc A, et al (2012) Condition-dependent transcriptome reveals high-level regulatory architecture in Bacillus subtilis. Science 335: 1103–1106. 22383849 10.1126/science.1206848

[32] Silvaggi JM, Perkins JB, Losick R (2005) Small untranslated RNA antitoxin in Bacillus subtilis. J Bacteriol 187: 6641–6650. 16166525 10.1128/JB.187.19.6641-6650.2005 PMC1251590

[33] Christensen QH, Martin N, Mansilla MC, de Mendoza D, Cronan JE (2011) A novel amidotransferase required for lipoic acid cofactor assembly in Bacillus subtilis. Mol Microbiol 80: 350–363. 21338421 10.1111/j.1365-2958.2011.07598.x PMC3088481

[34] Guillouard I, Auger S, Hullo MF, Chetouani F, Danchin A, et al (2002) Identification of Bacillus subtilis CysL, a regulator of the cysJI operon, which encodes sulfite reductase. J Bacteriol 184: 4681–4689. 12169591 10.1128/JB.184.17.4681-4689.2002 PMC135269

[35] Gaballa A, Wang T, Ye RW, Helmann JD (2002) Functional analysis of the Bacillus subtilis Zur regulon. J Bacteriol 184: 6508–6514. 12426338 10.1128/JB.184.23.6508-6514.2002 PMC135443

[36] Bayle L, Chimalapati S, Schoehn G, Brown J, Vernet T, et al (2011) Zinc uptake by Streptococcus pneumoniae depends on both AdcA and AdcAII and is essential for normal bacterial morphology and virulence. Mol Microbiol 82: 904–916. 22023106 10.1111/j.1365-2958.2011.07862.x

[37] Ogura Y, Imai Y, Ogasawara N, Moriya S (2001) Autoregulation of the dnaA-dnaN operon and effects of DnaA protein levels on replication initiation in Bacillus subtilis. J Bacteriol 183: 3833–3841. 11395445 10.1128/JB.183.13.3833-3841.2001 PMC95264

[38] Bhavsar AP, Brown ED (2006) Cell wall assembly in Bacillus subtilis: how spirals and spaces challenge paradigms. Mol Microbiol 60: 1077–1090. 16689786 10.1111/j.1365-2958.2006.05169.x

[39] Vollmer W, Blanot D, de Pedro MA (2008) Peptidoglycan structure and architecture. FEMS Microbiol Rev 32: 149–167. 18194336 10.1111/j.1574-6976.2007.00094.x

[40] Spizizen J (1958) Transformation of biochemically deficient strains of *Bacillus subtilis* by deoxyribonucleate. Proc Natl Acad Sci U S A 44: 407–408. 10.1073/pnas.44.10.1072 PMC528696 16590310

[41] Nicolas P, Leduc A, Robin S, Rasmussen S, Jarmer H, et al (2009) Transcriptional landscape estimation from tiling array data using a model of signal shift and drift. Bioinformatics 25: 2341–2347. 19561016 10.1093/bioinformatics/btp395 PMC2735659

[42] Strimmer K (2008) A unified approach to false discovery rate estimation. BMC Bioinformatics 9: 303. 18613966 10.1186/1471-2105-9-303 PMC2475539

[43] Putzer H, Gendron N, Grunberg-Manago M (1992) Co-ordinate expression of the two threonyl-tRNA synthetase genes in *Bacillus subtilis*: control by transcriptional antitermination involving a conserved regulatory sequence. EMBO J 11: 3117–3127. 1379177 10.1002/j.1460-2075.1992.tb05384.x PMC556796

[44] Barrick JE, Corbino KA, Winkler WC, Nahvi A, Mandal M, et al (2004) New RNA motifs suggest an expanded scope for riboswitches in bacterial genetic control. Proc Natl Acad Sci U S A 101: 6421–6426. 15096624 10.1073/pnas.0308014101 PMC404060

[45] Ibrahim M, Nicolas P, Bessieres P, Bolotin A, Monnet V, et al (2007) A genome-wide survey of short coding sequences in streptococci. Microbiology 153: 3631–3644. 17975071 10.1099/mic.0.2007/006205-0

[46] Gardner PP, Daub J, Tate J, Moore BL, Osuch IH, et al (2011) Rfam: Wikipedia, clans and the “decimal” release. Nucleic Acids Res 39: D141–145. 21062808 10.1093/nar/gkq1129 PMC3013711

[47] Sterk P, Kersey PJ, Apweiler R (2006) Genome reviews: Standardizing content and representation of information about complete genomes. OMICS 10: 114. 16901215 10.1089/omi.2006.10.114

