# Supplementary figures and images for: Bacillus subtilis RNase Y Activity In Vivo Analysed by Tiling Microarrays
Source: PLoS One. 2013 Jan 10;8(1):e54062. doi: 10.1371/journal.pone.0054062 (PMC3542257; doi:10.1371/journal.pone.0054062)

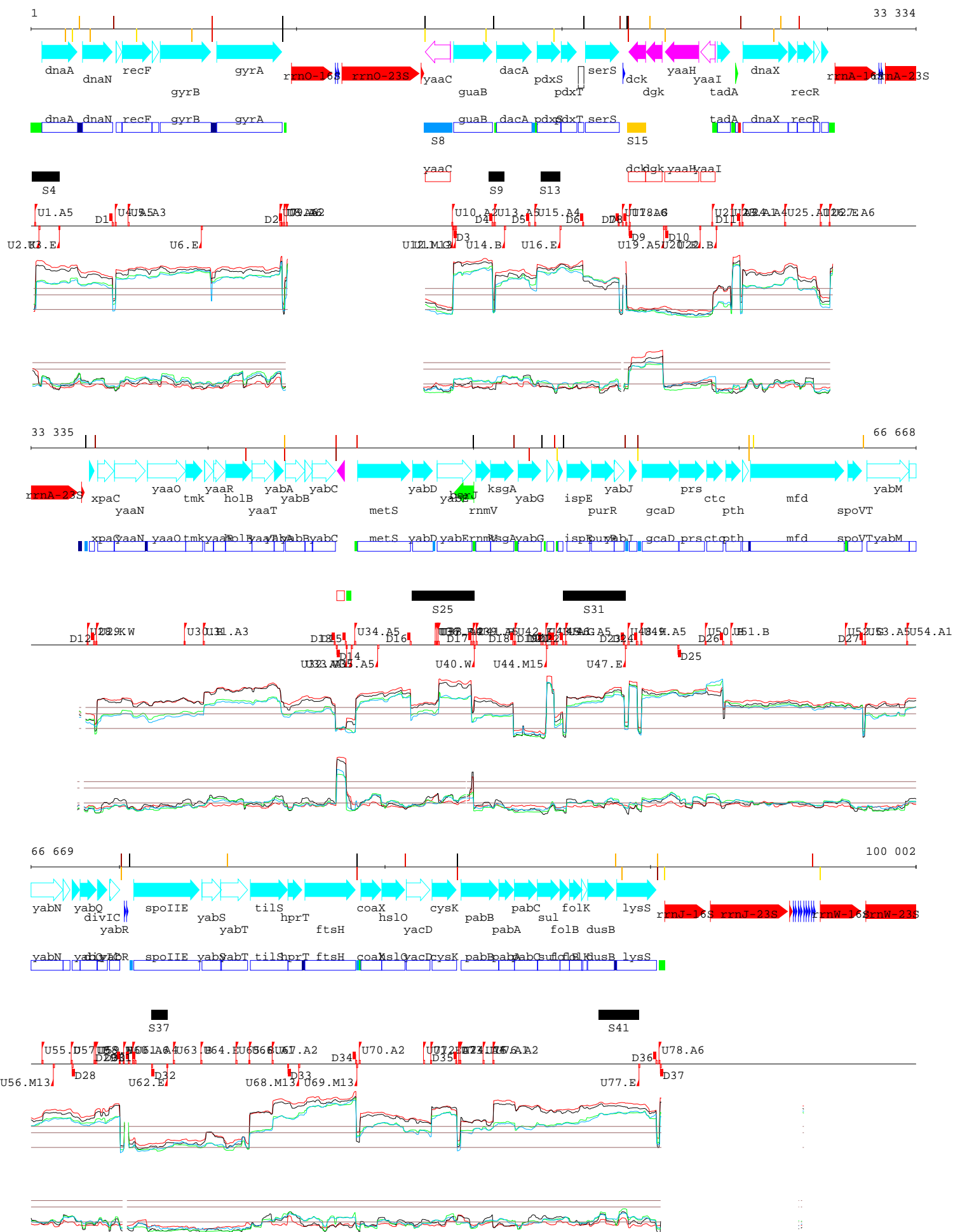

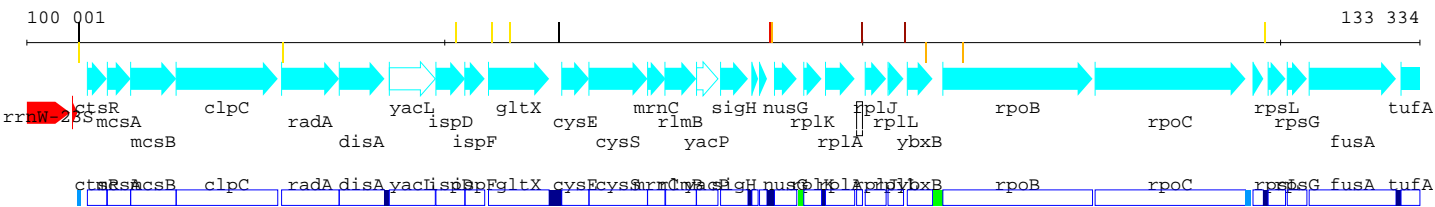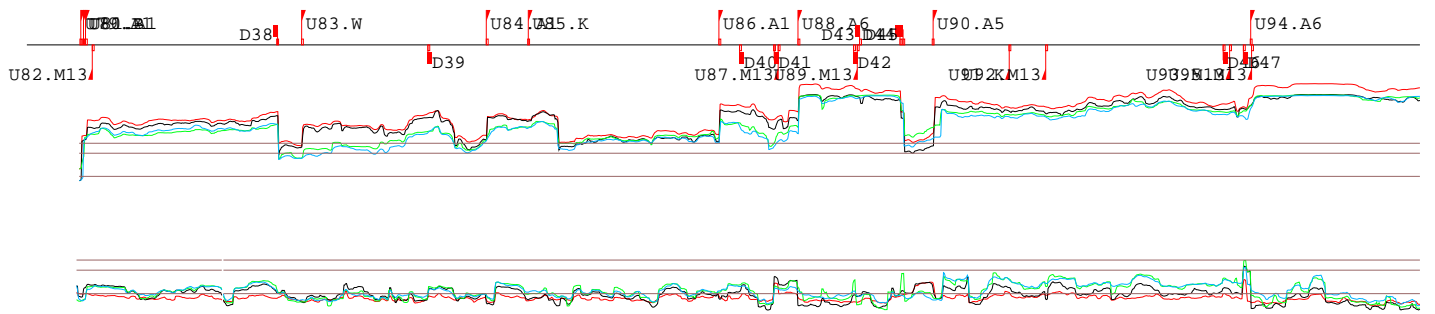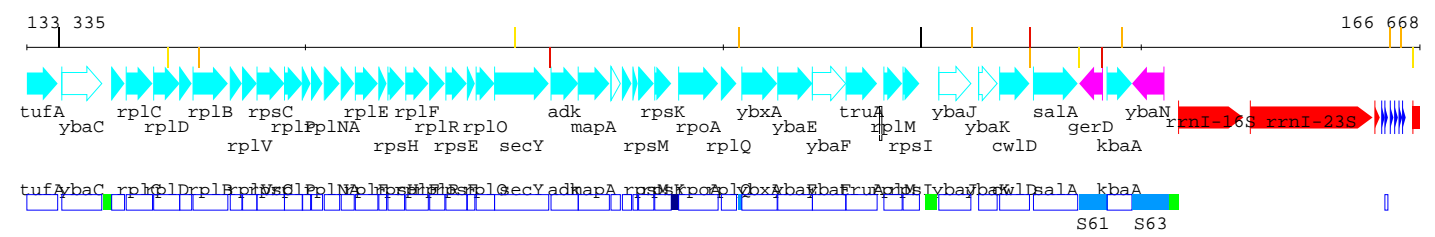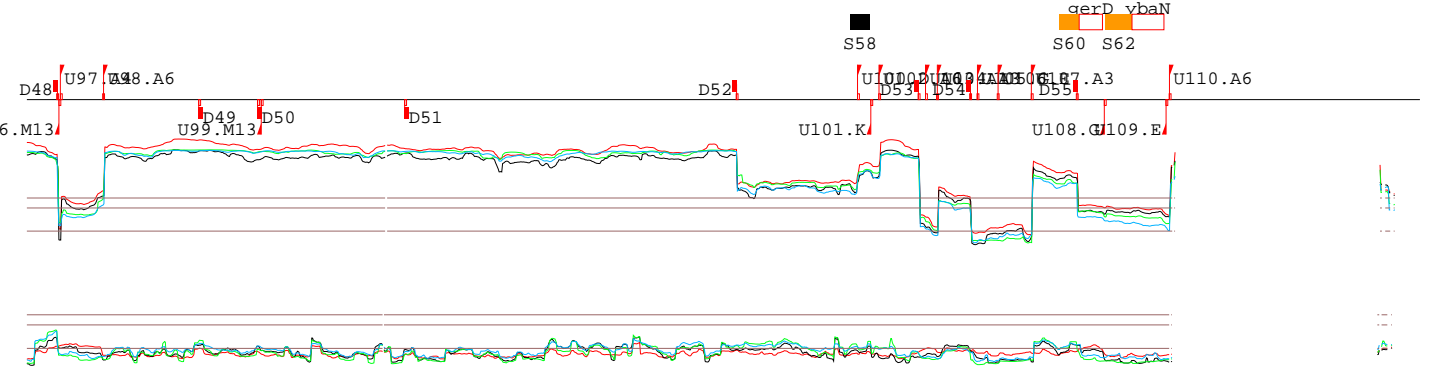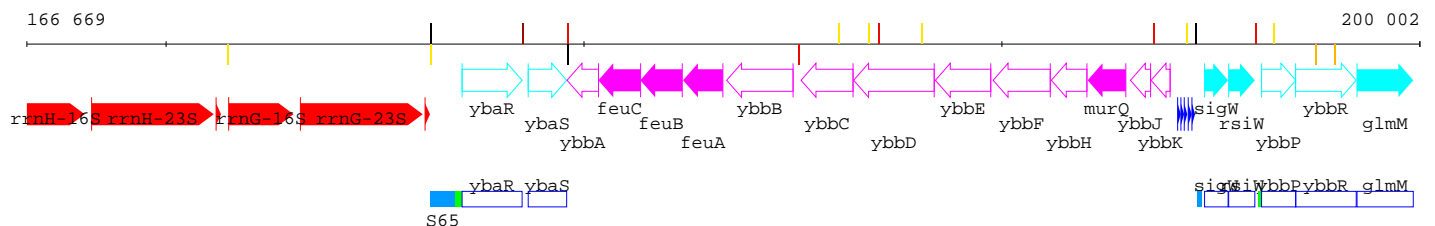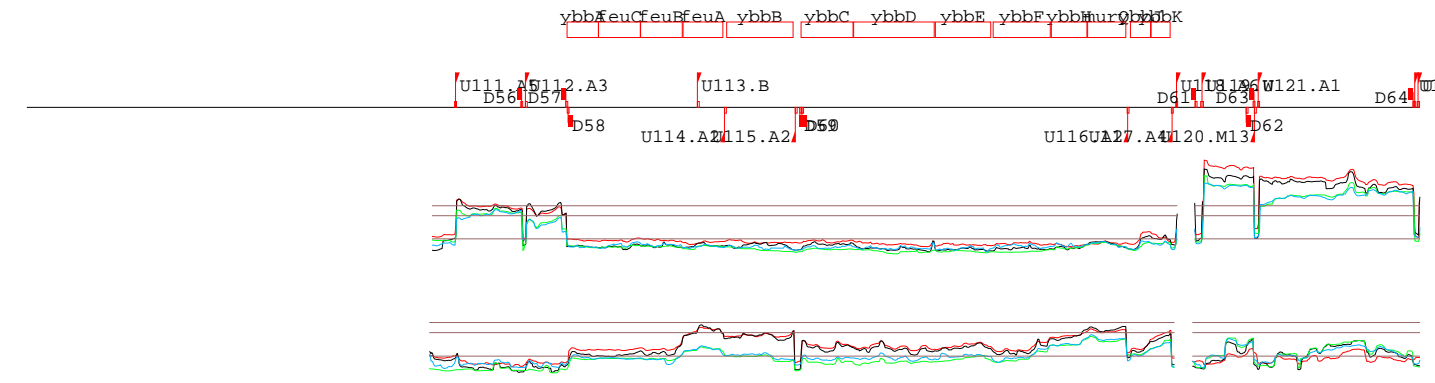

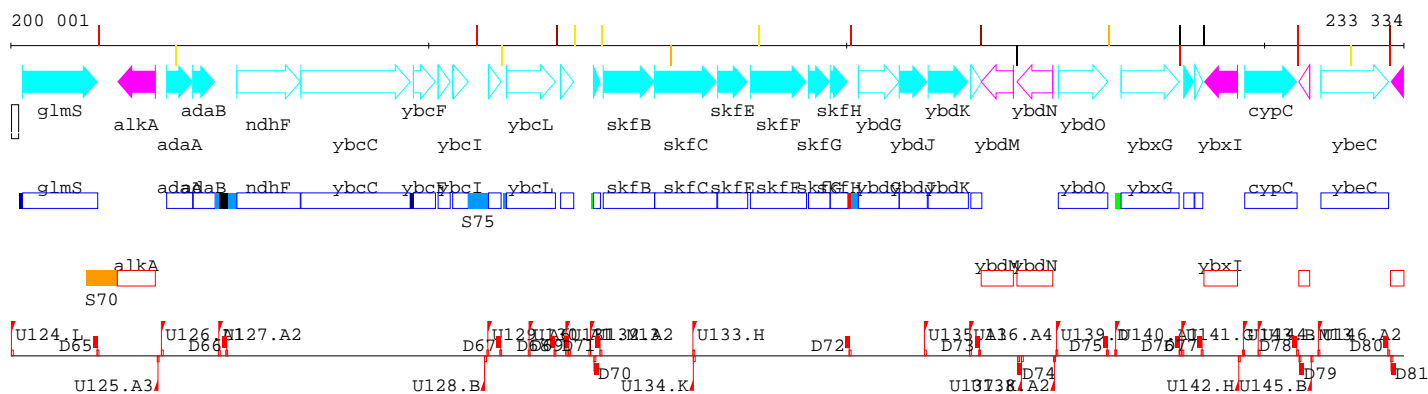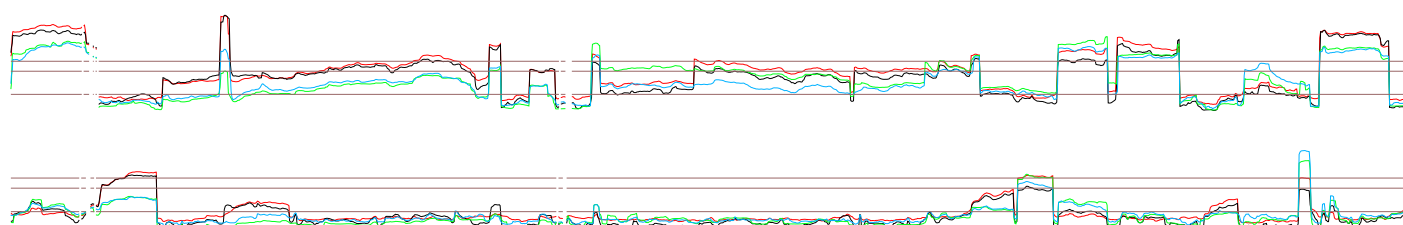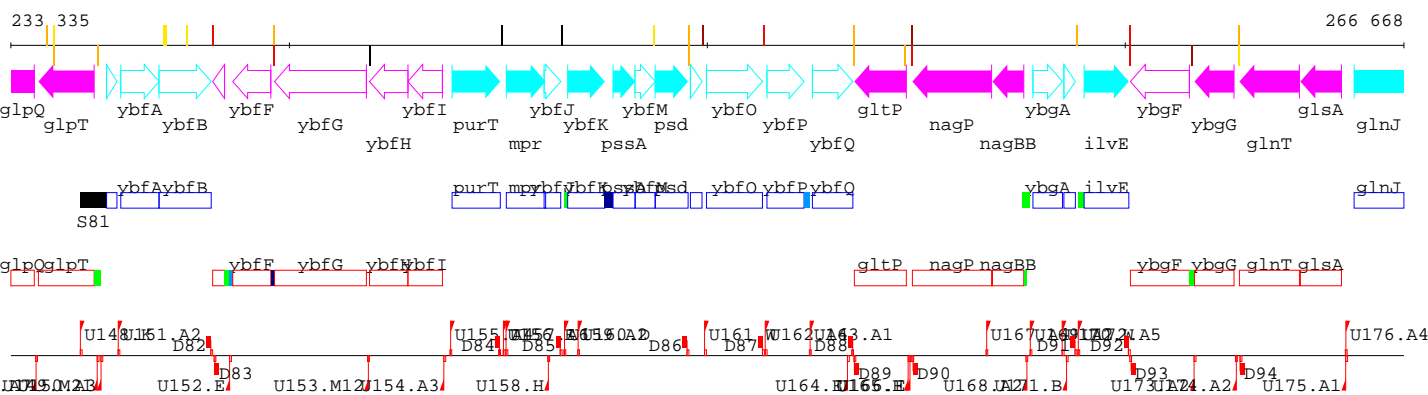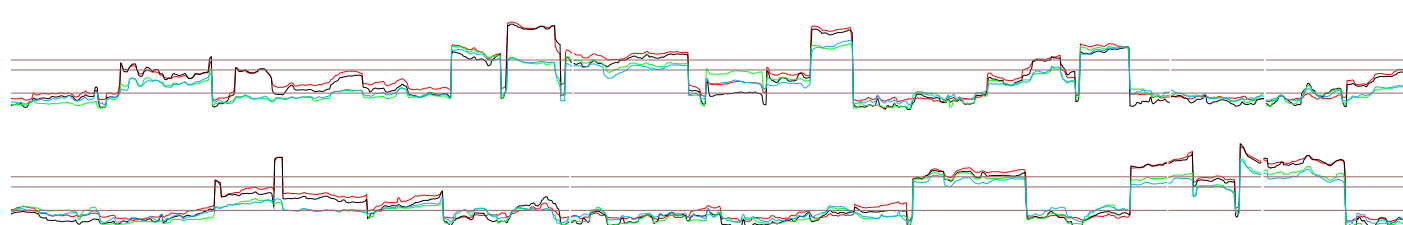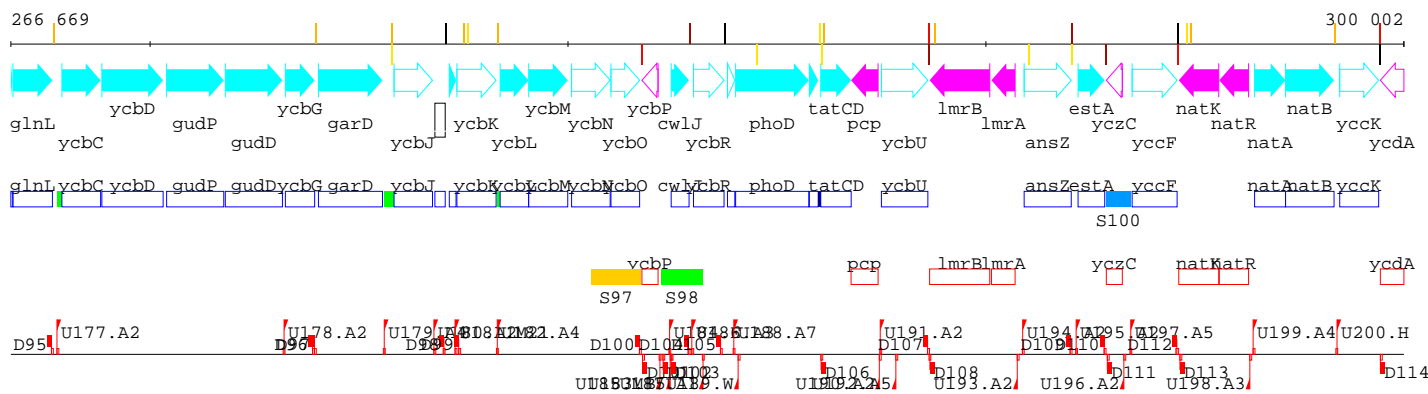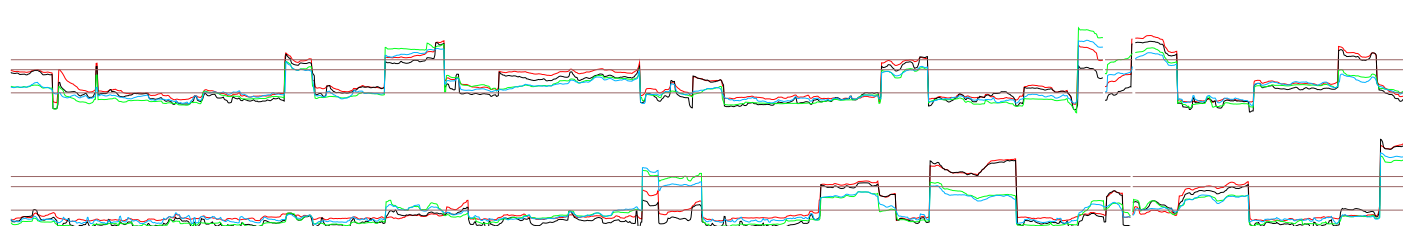

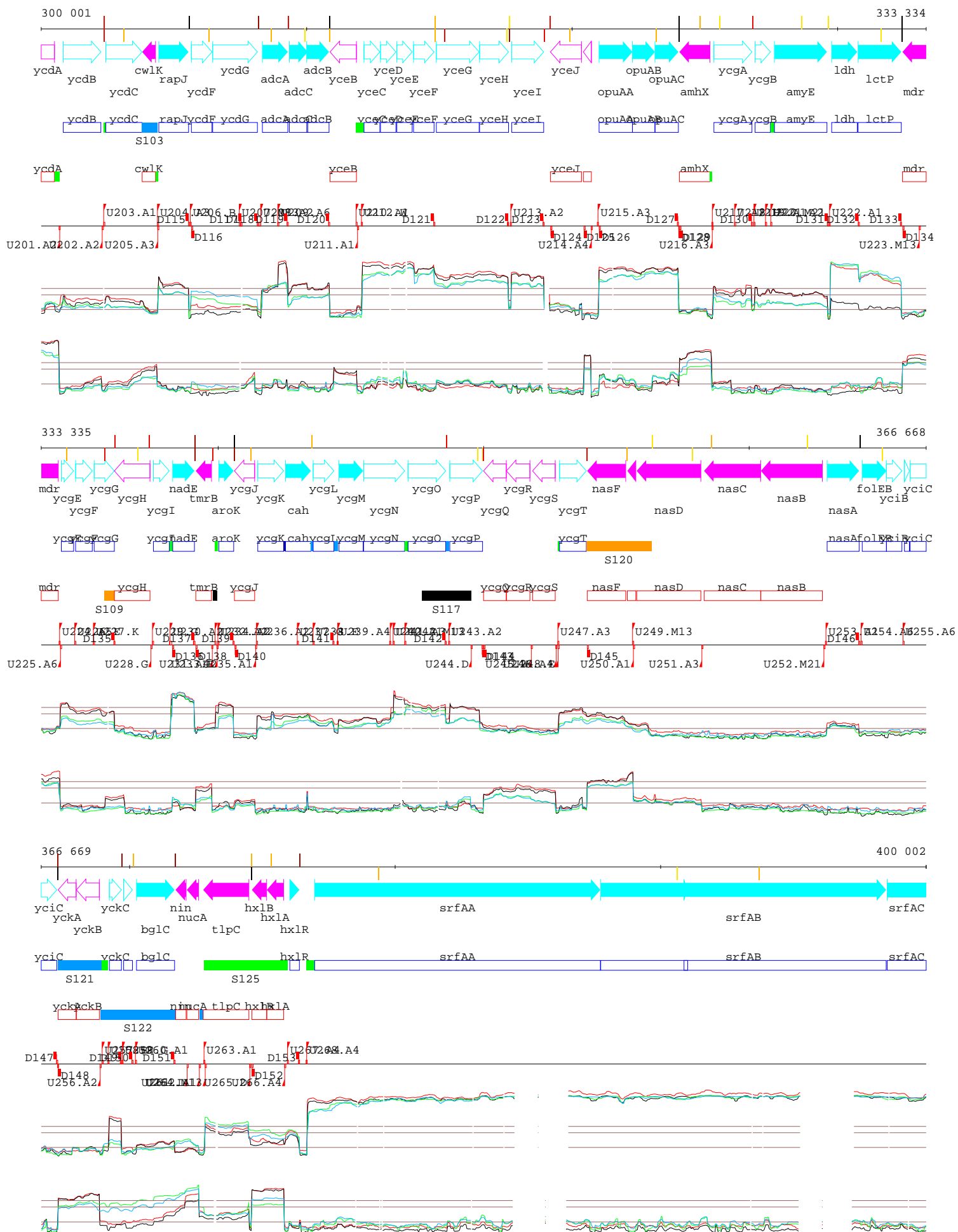

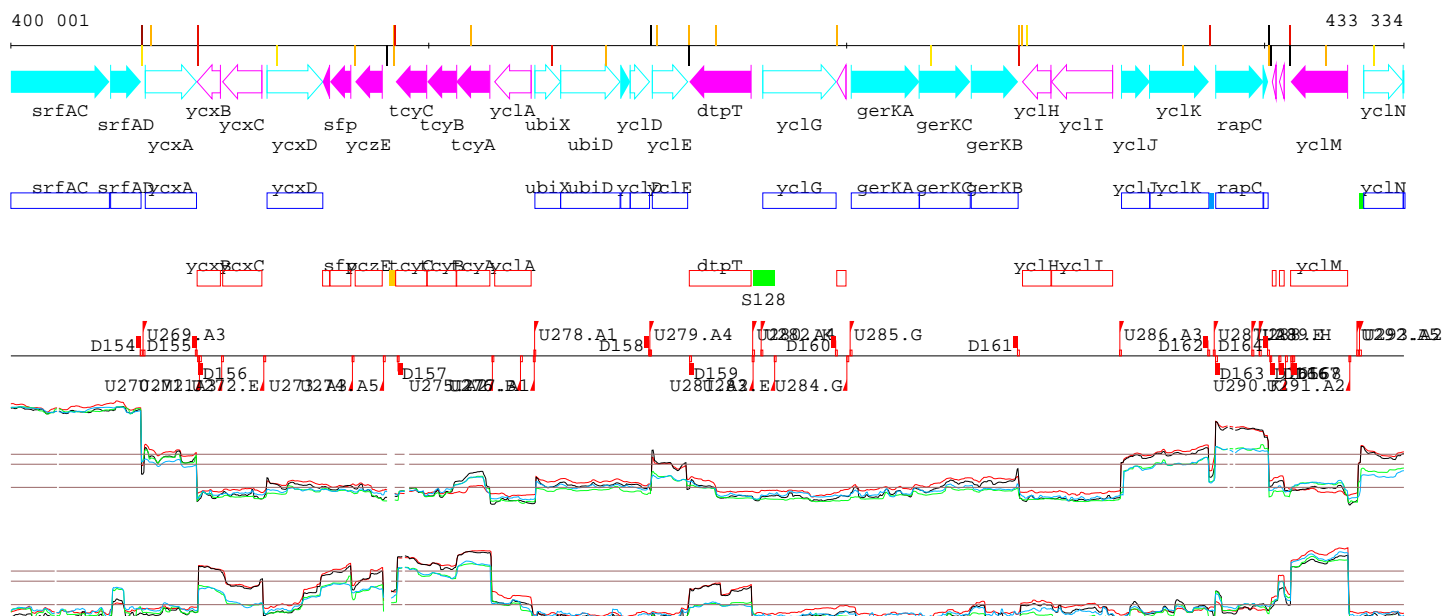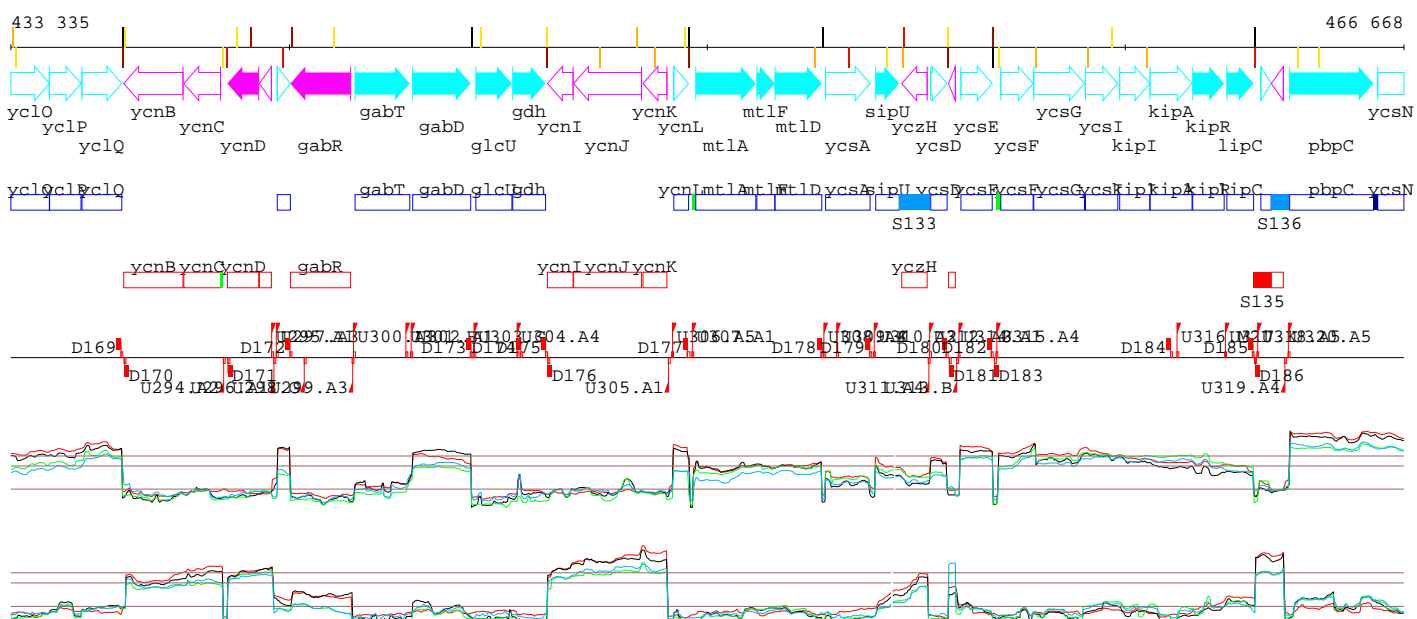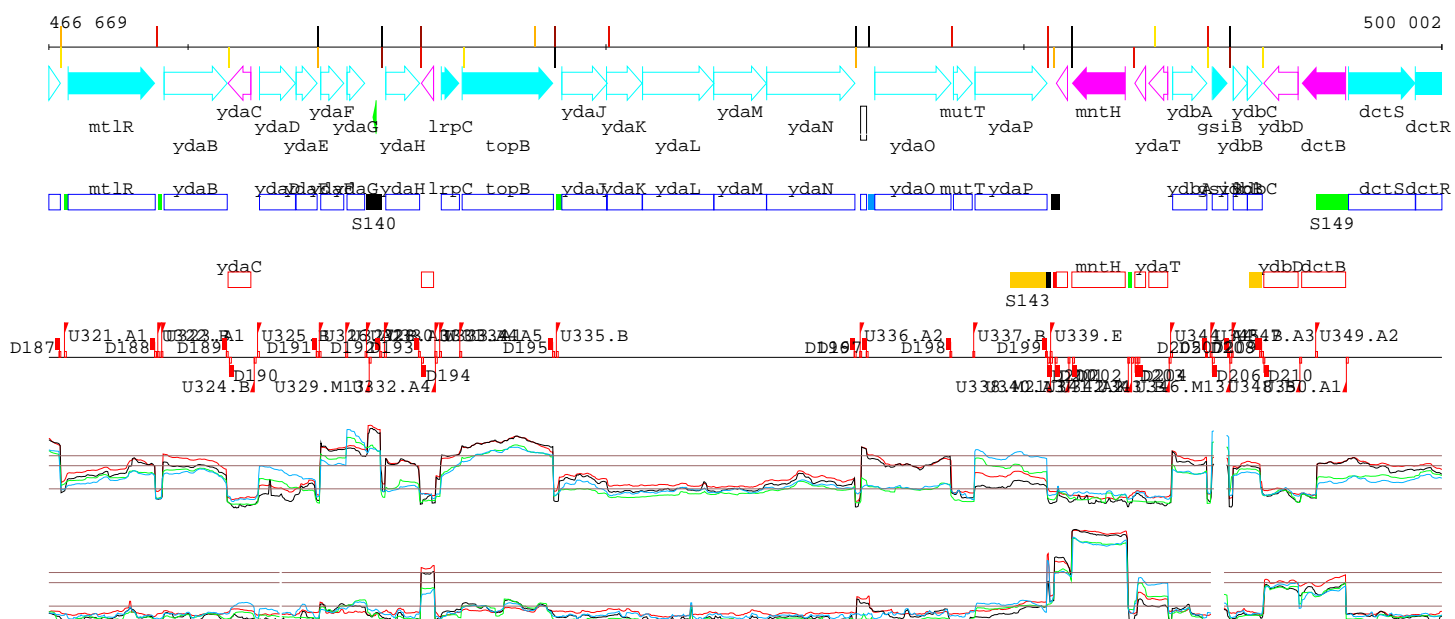

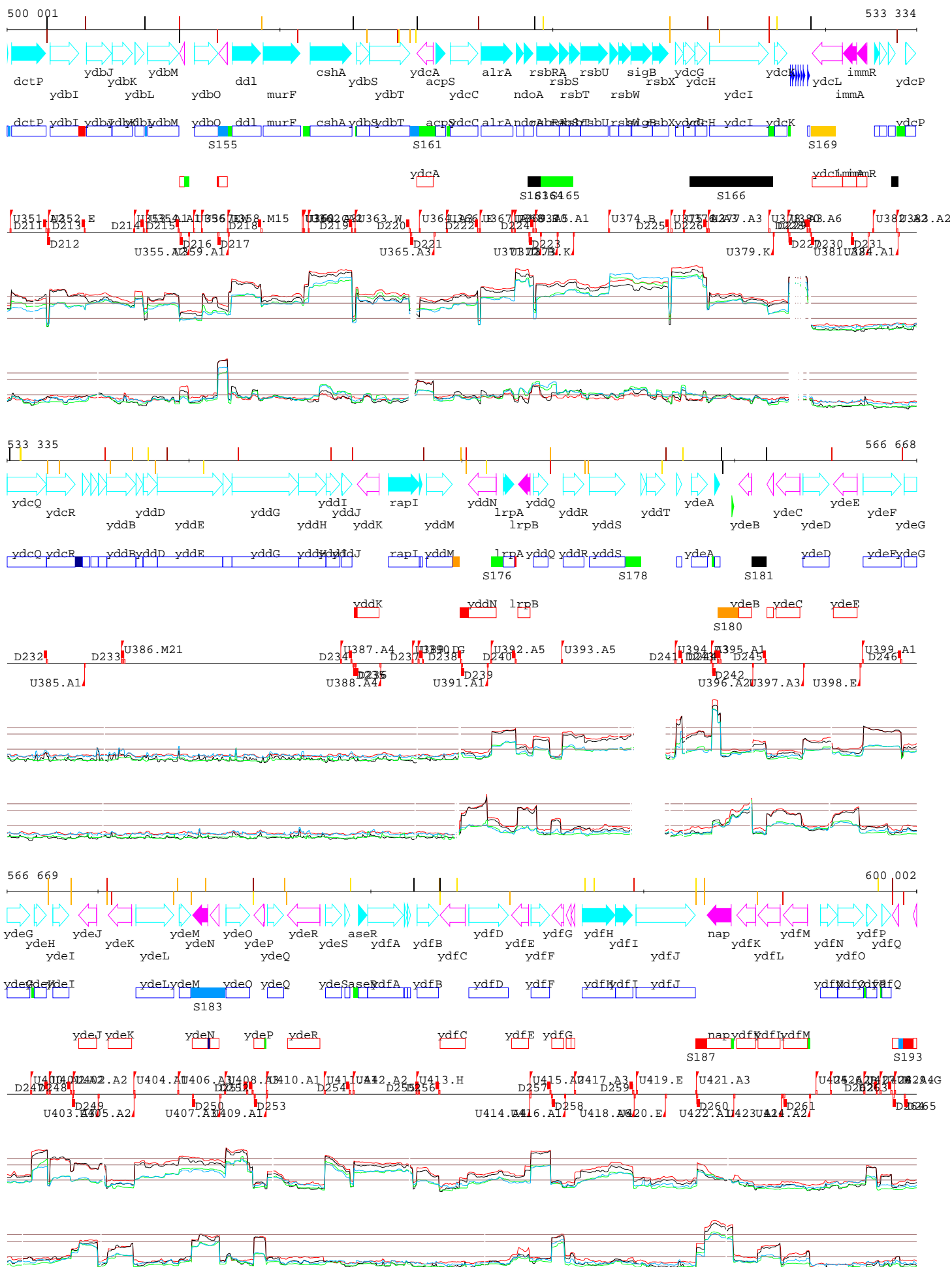

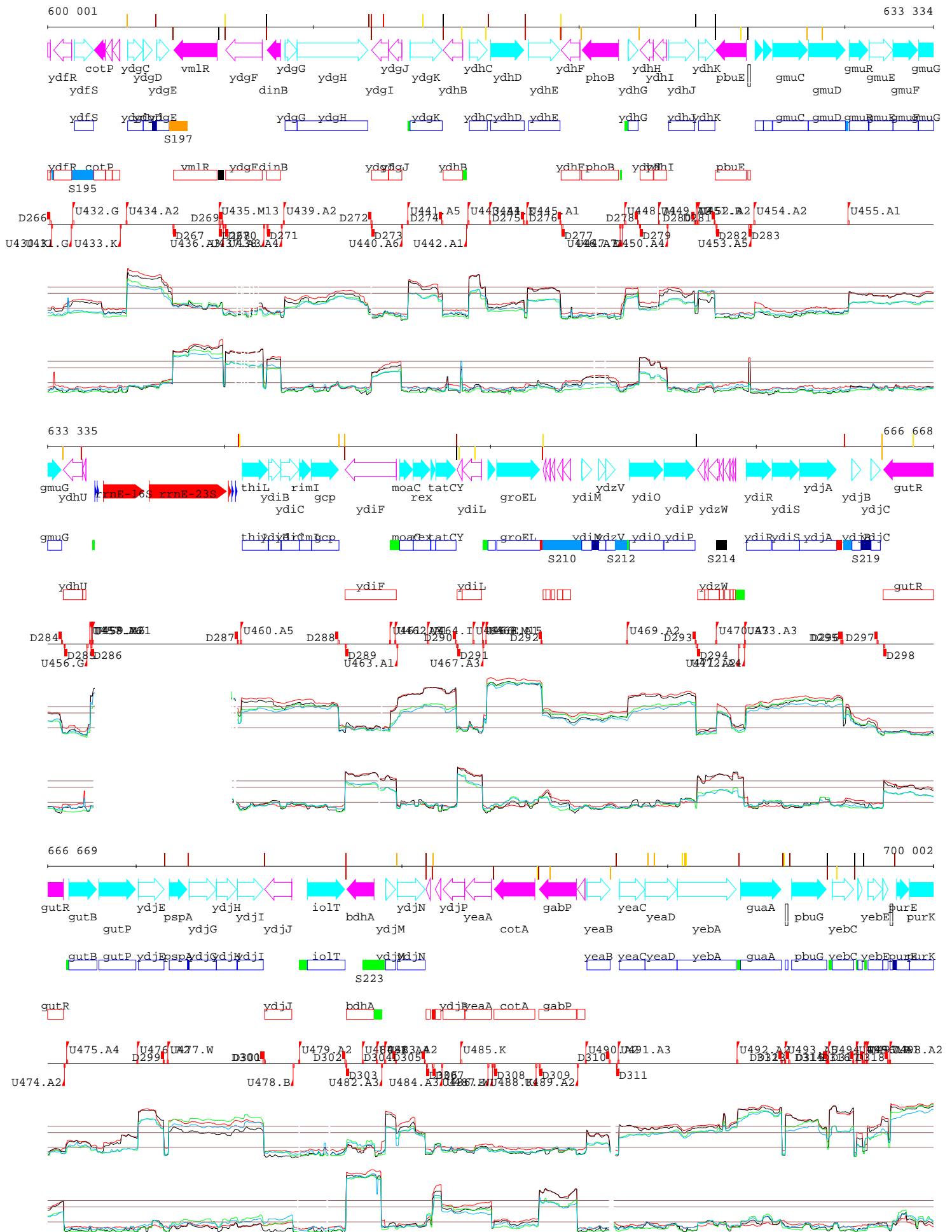

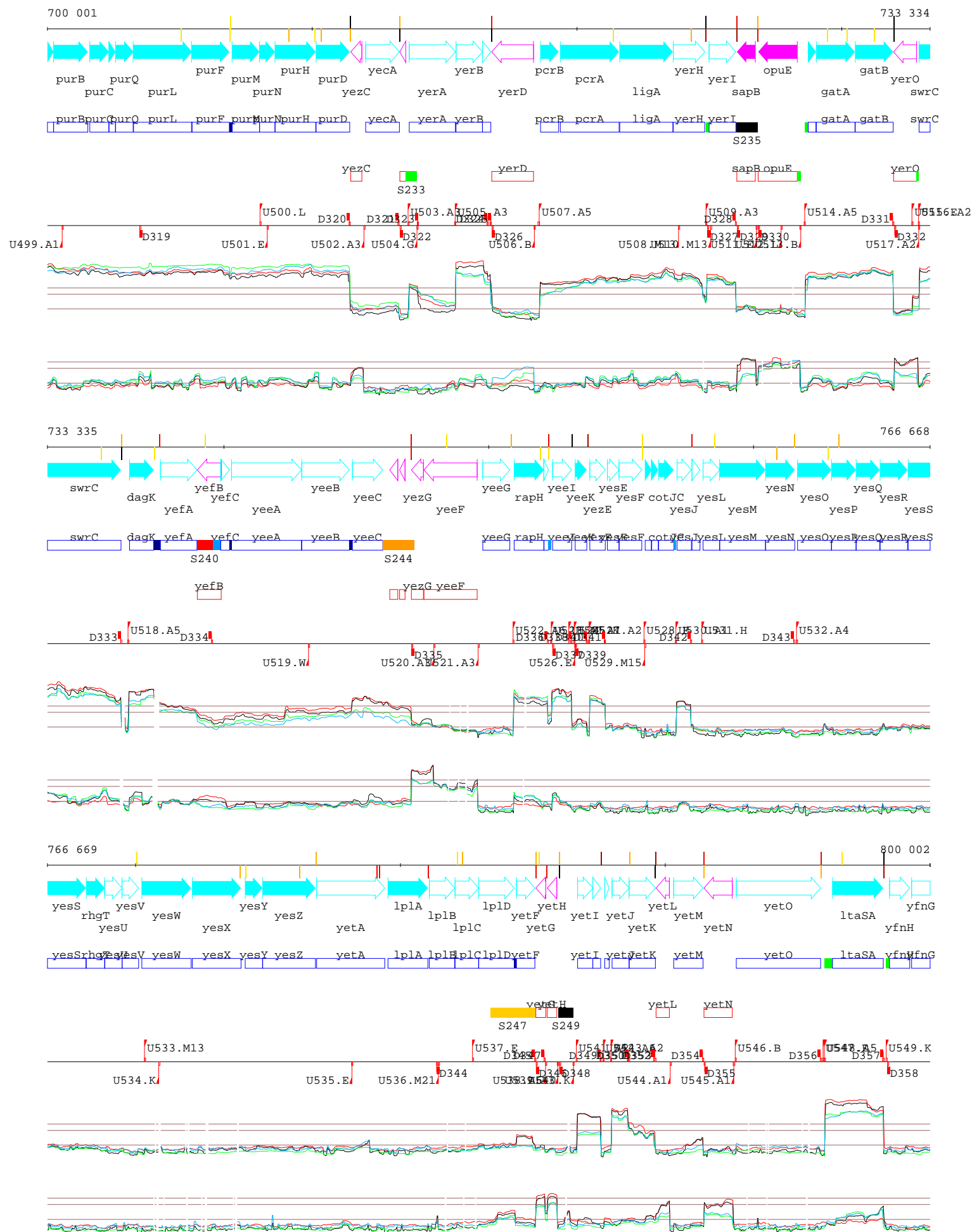

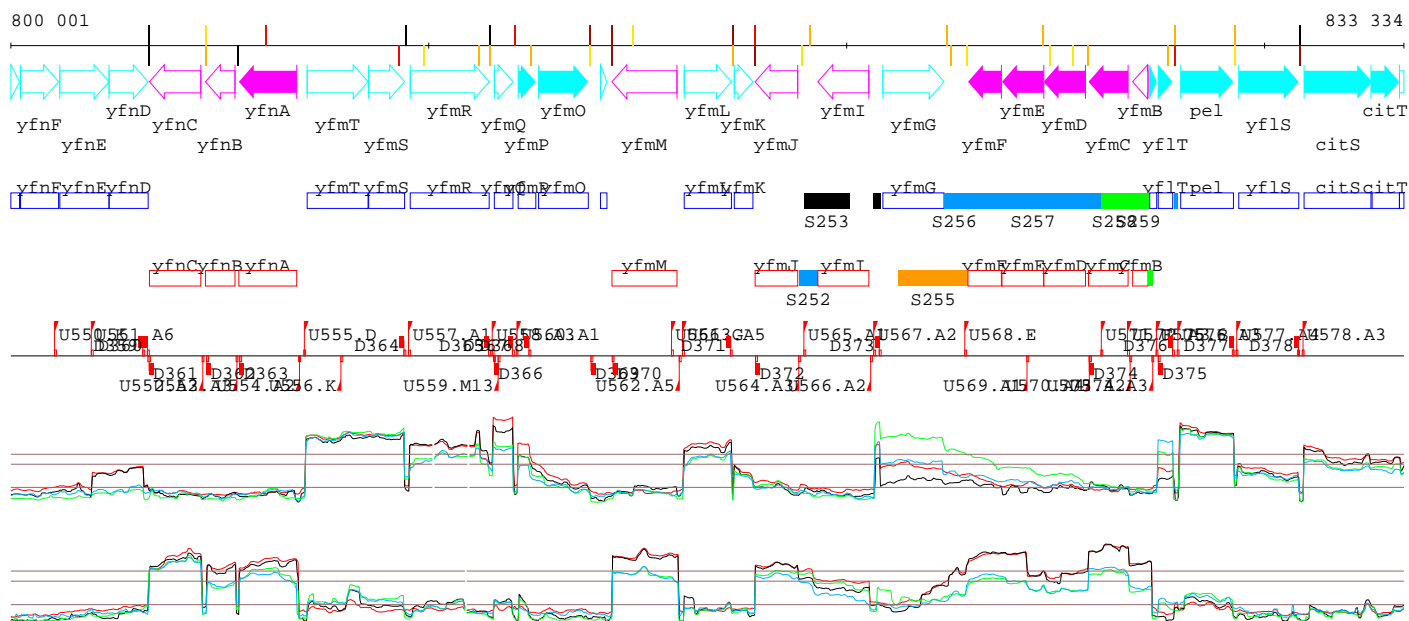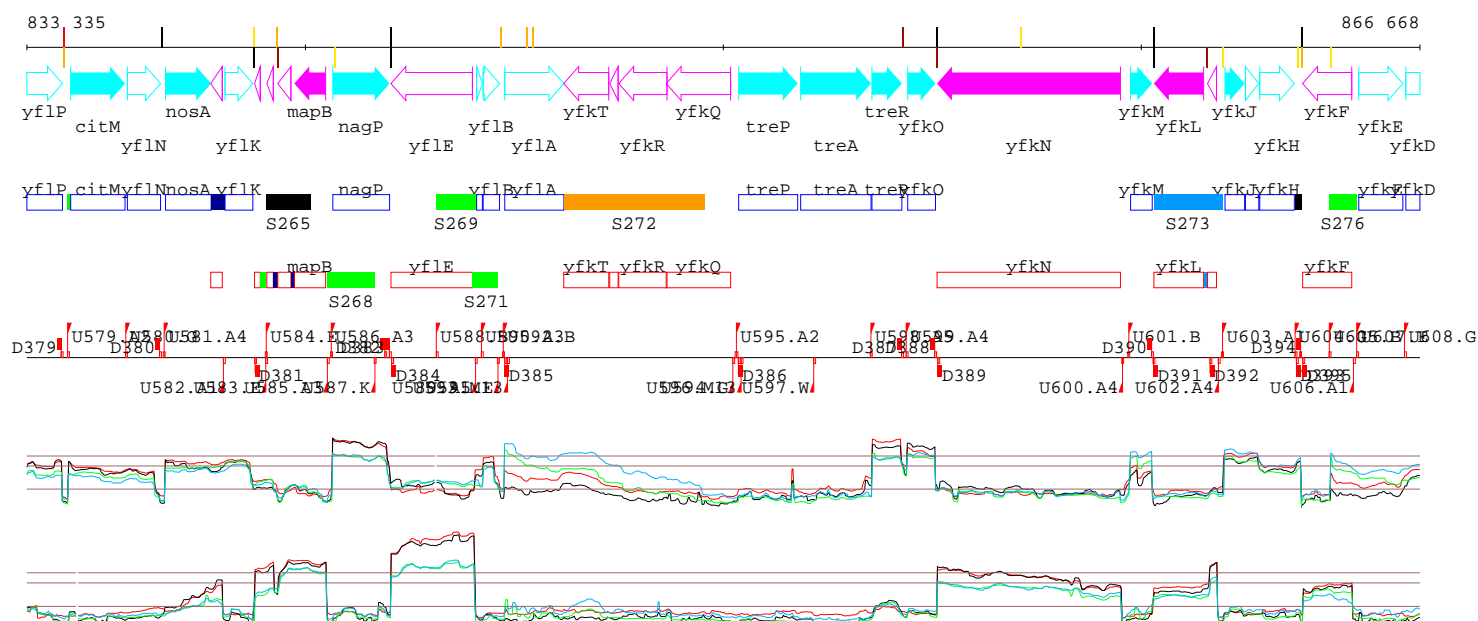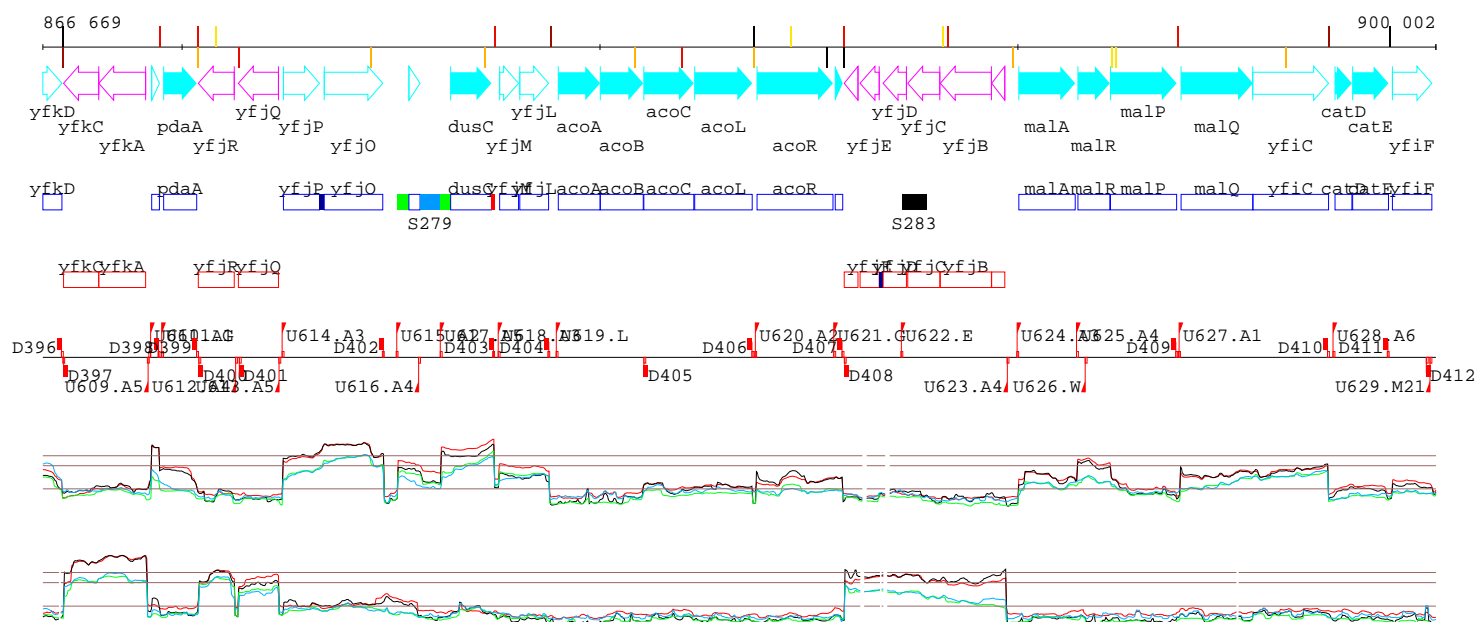

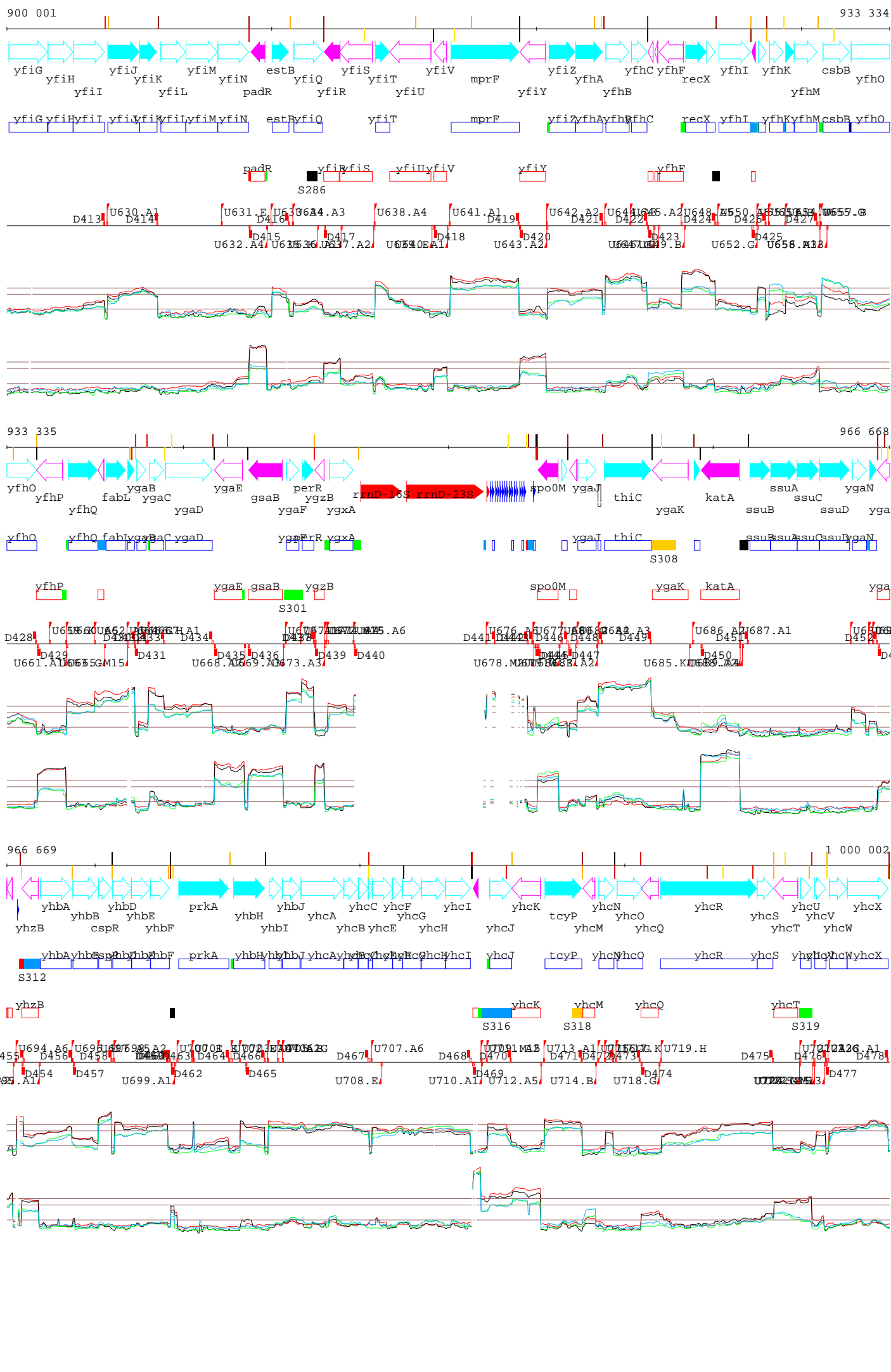



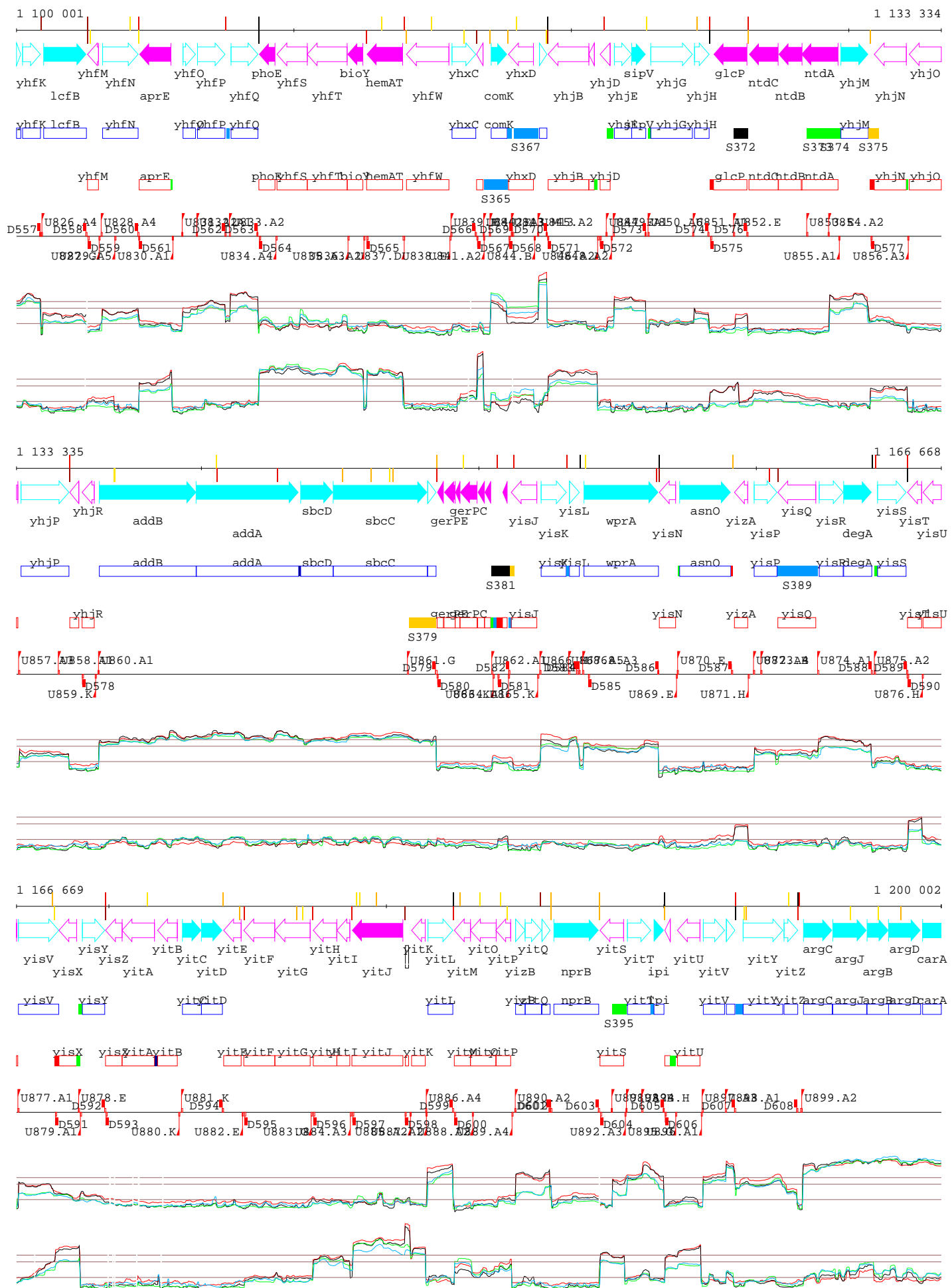

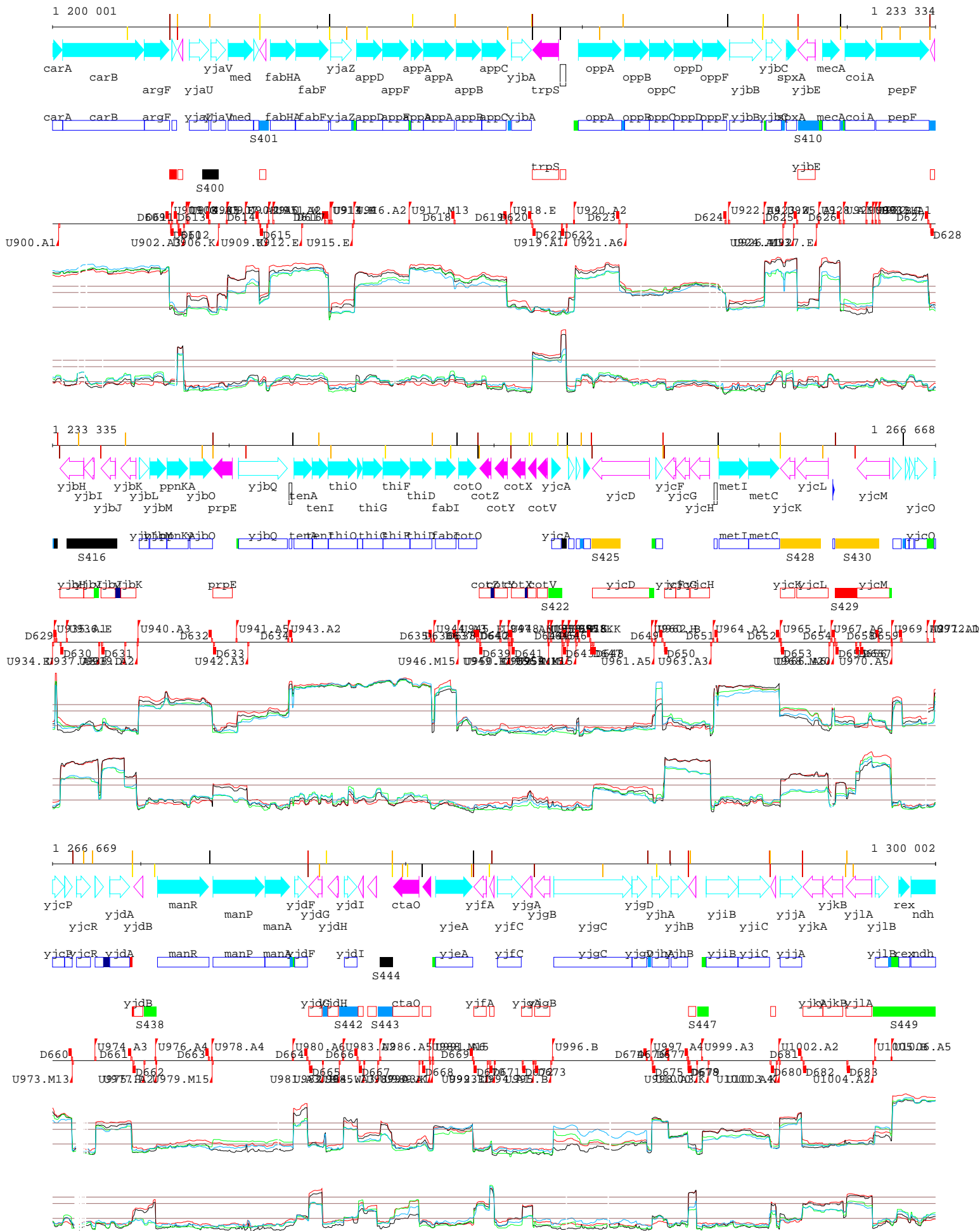

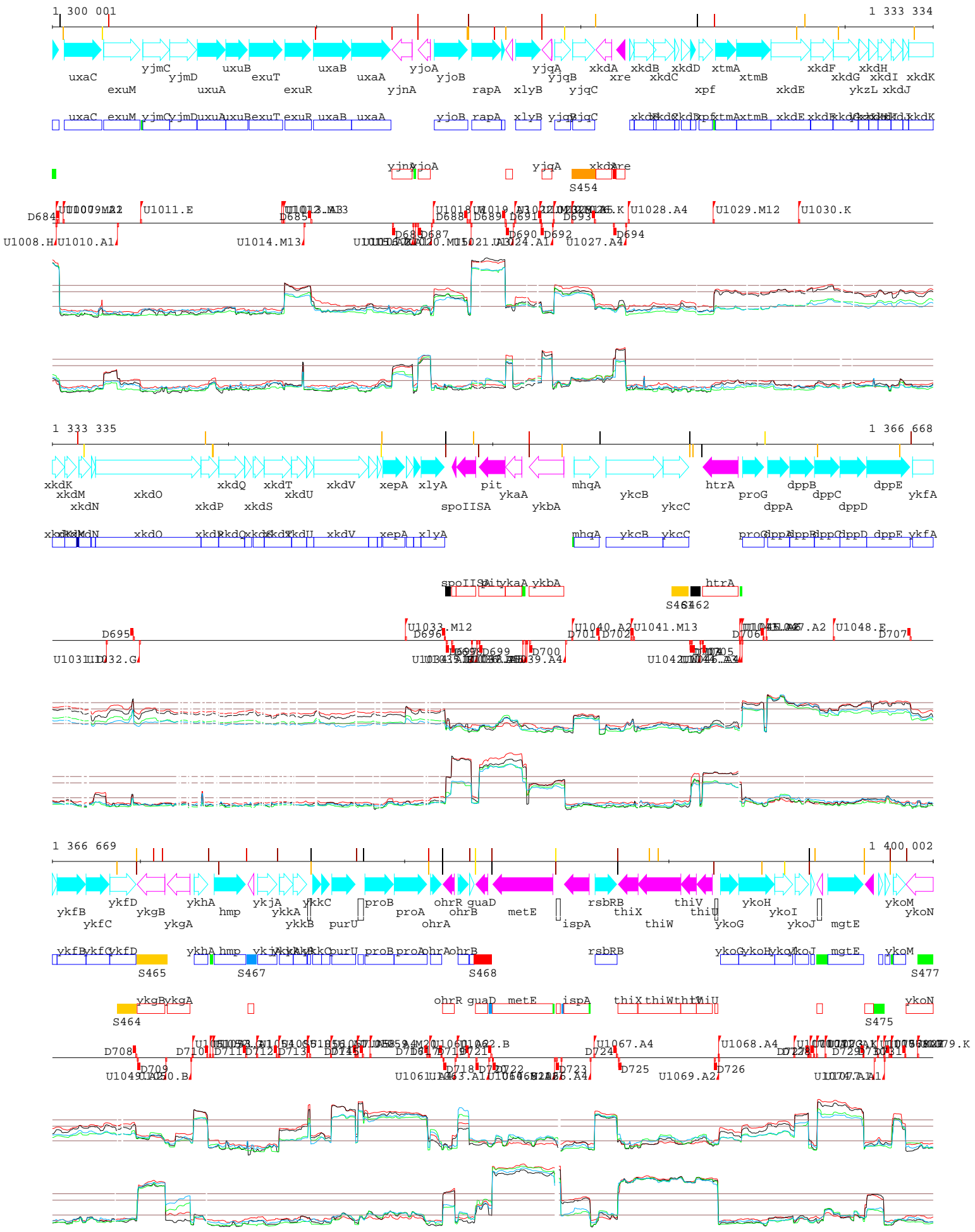

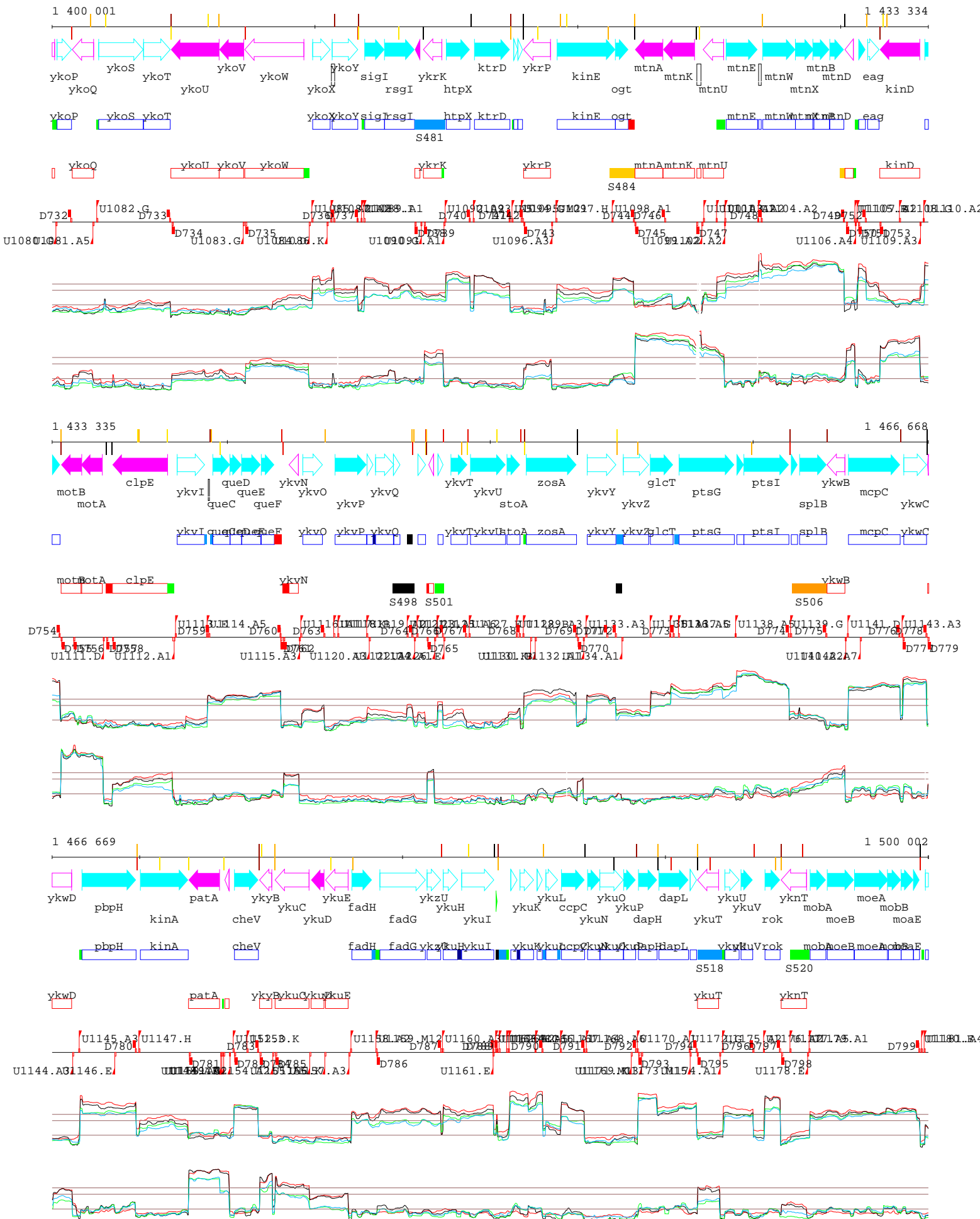

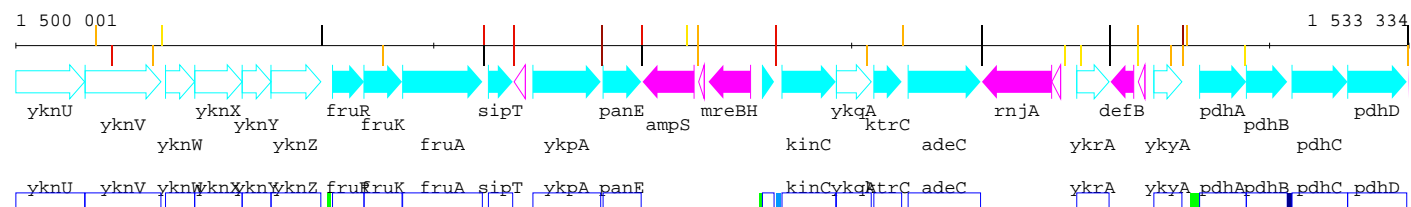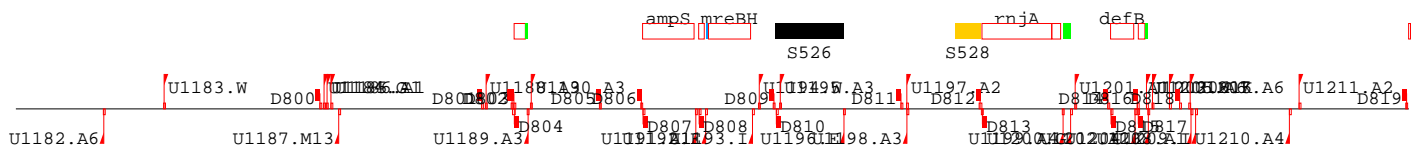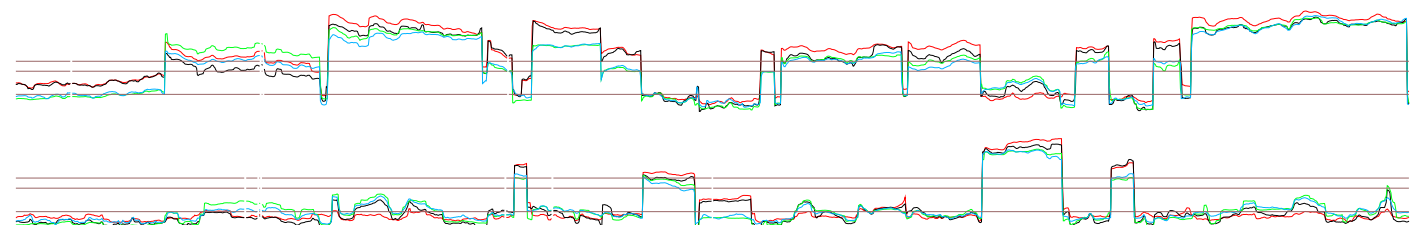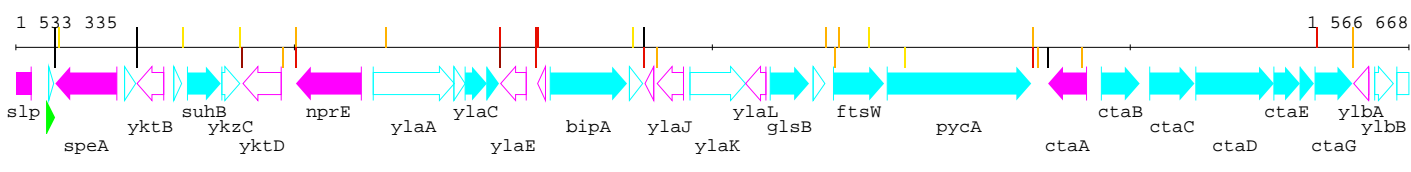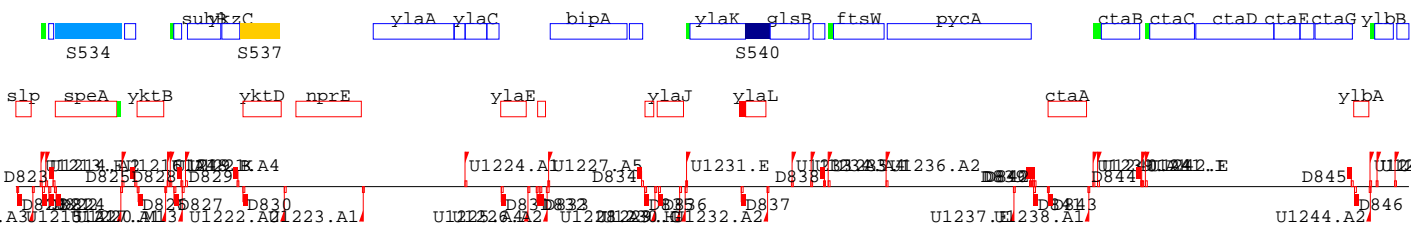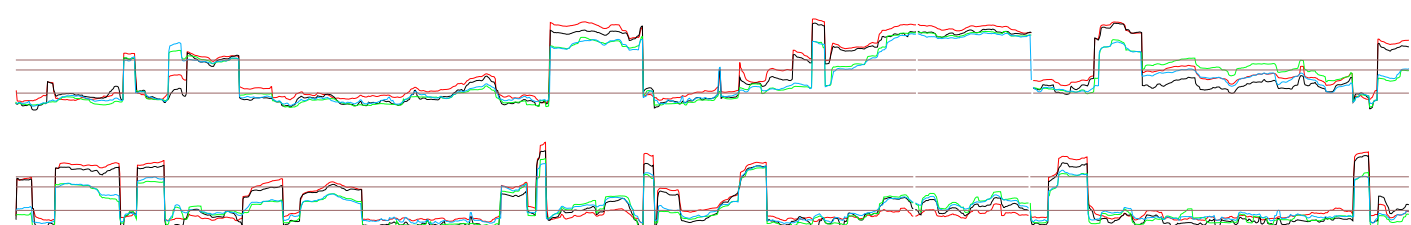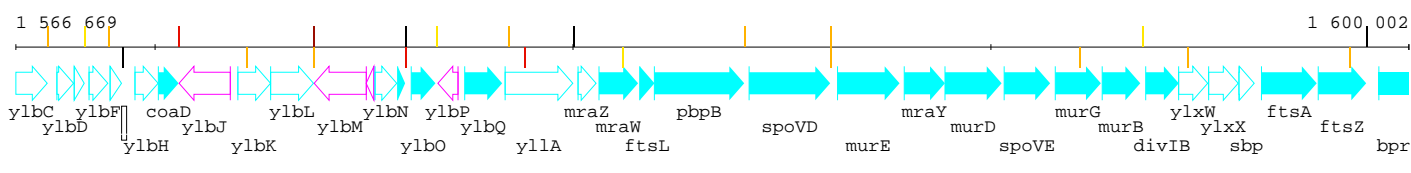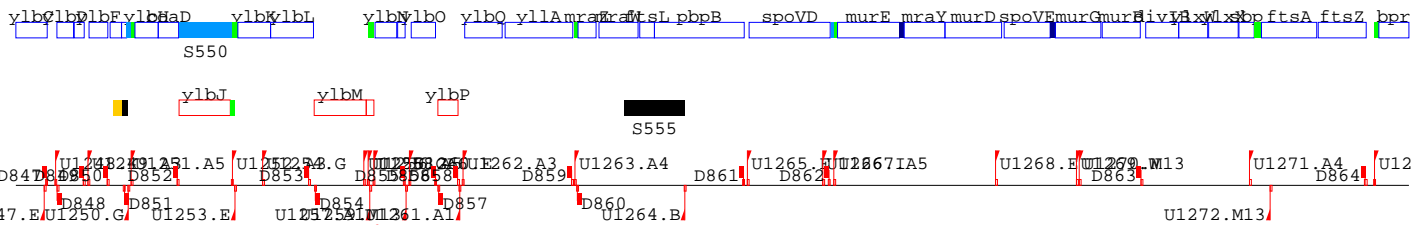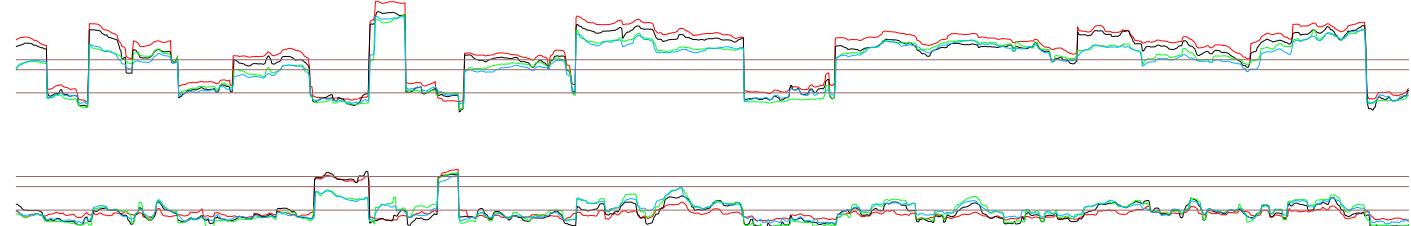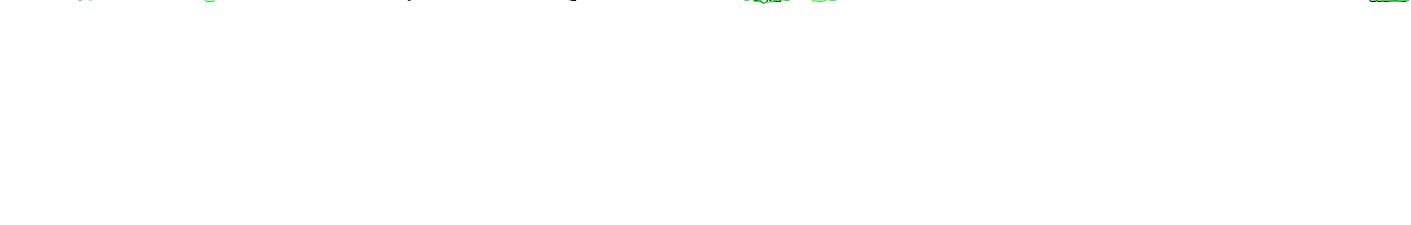

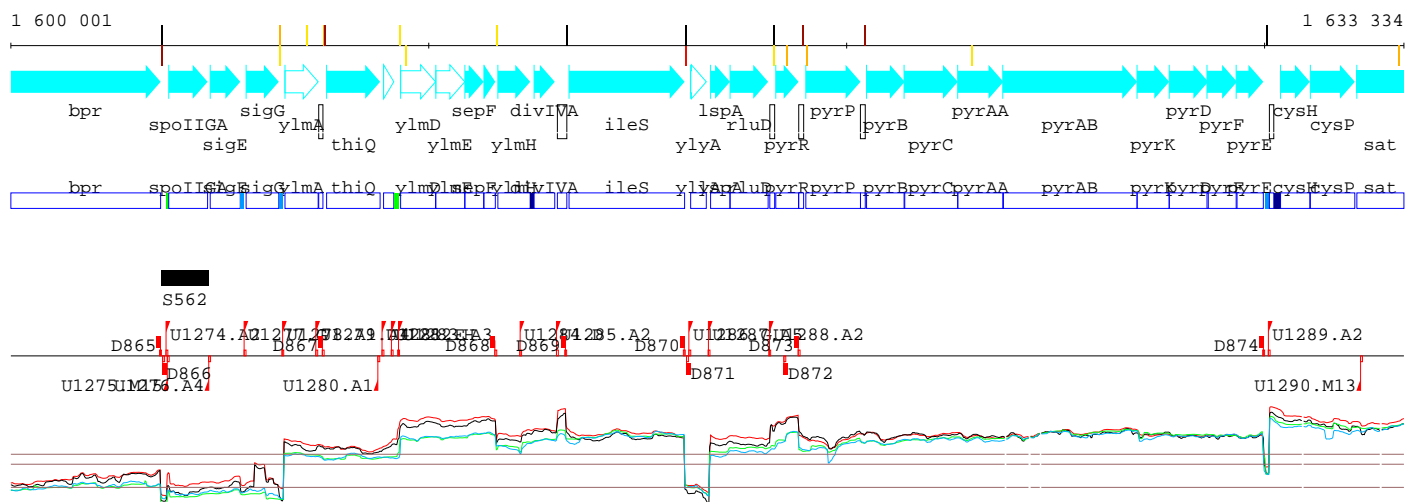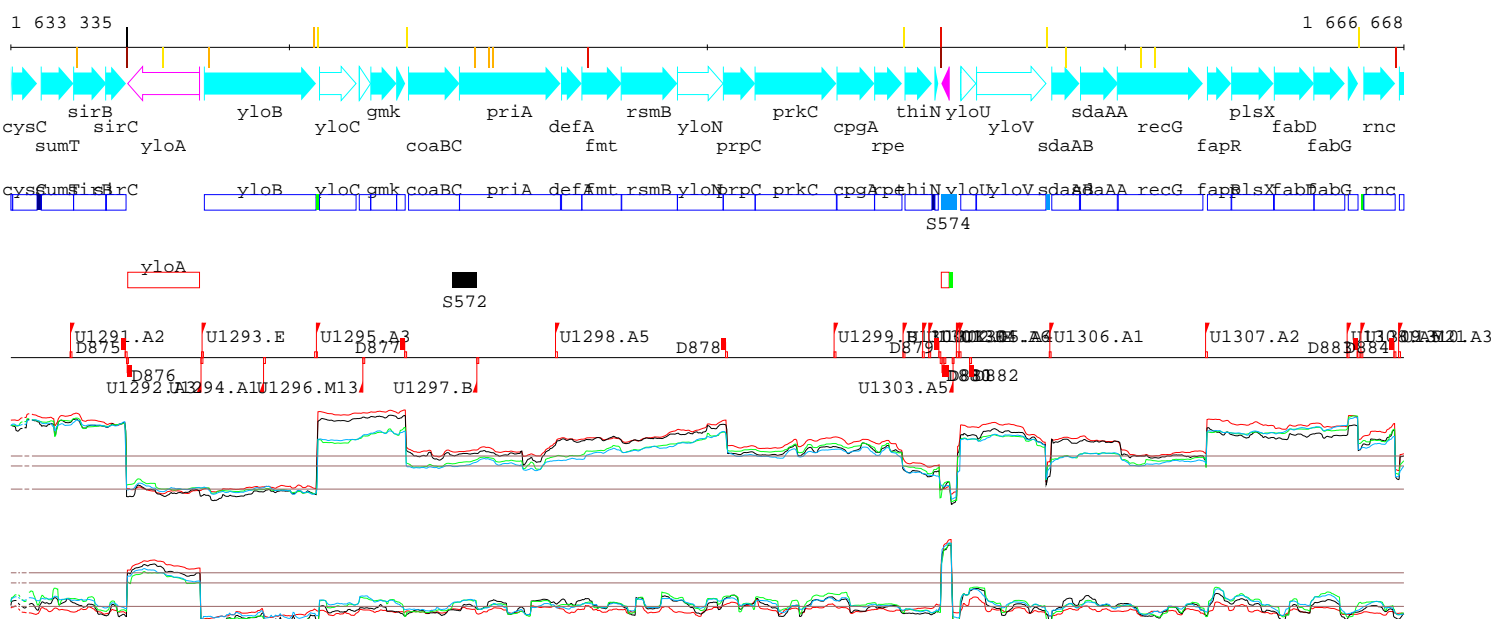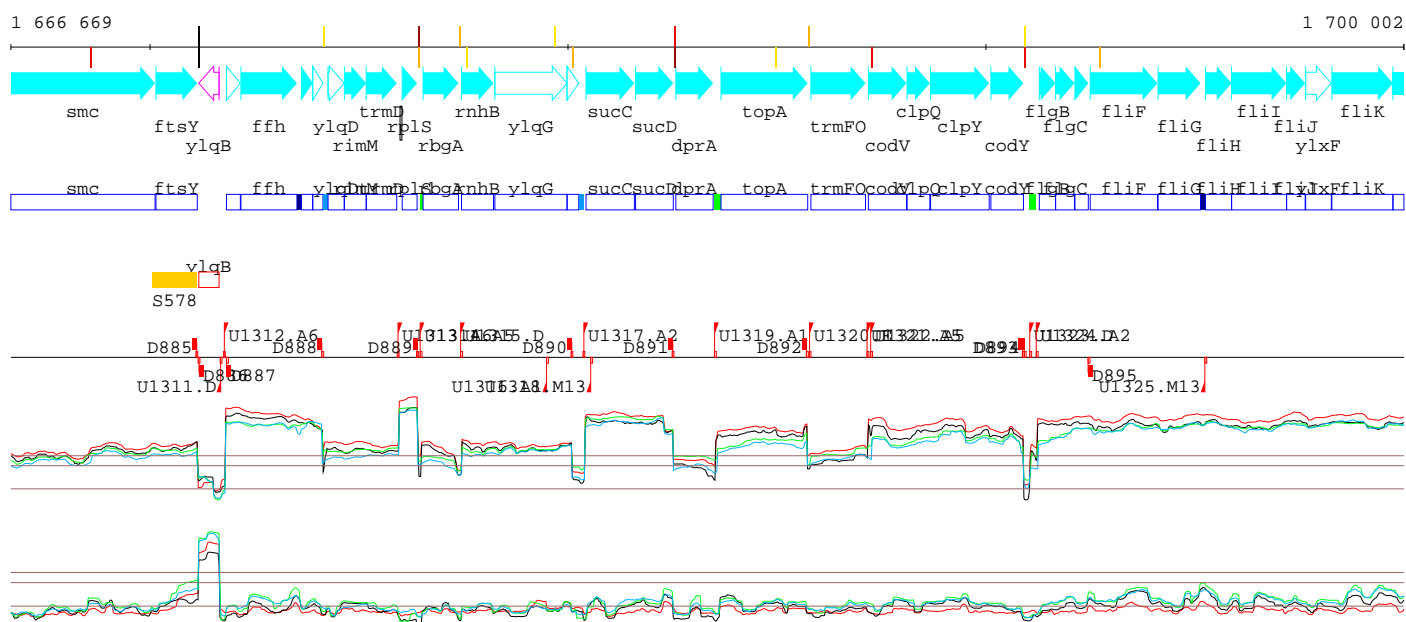

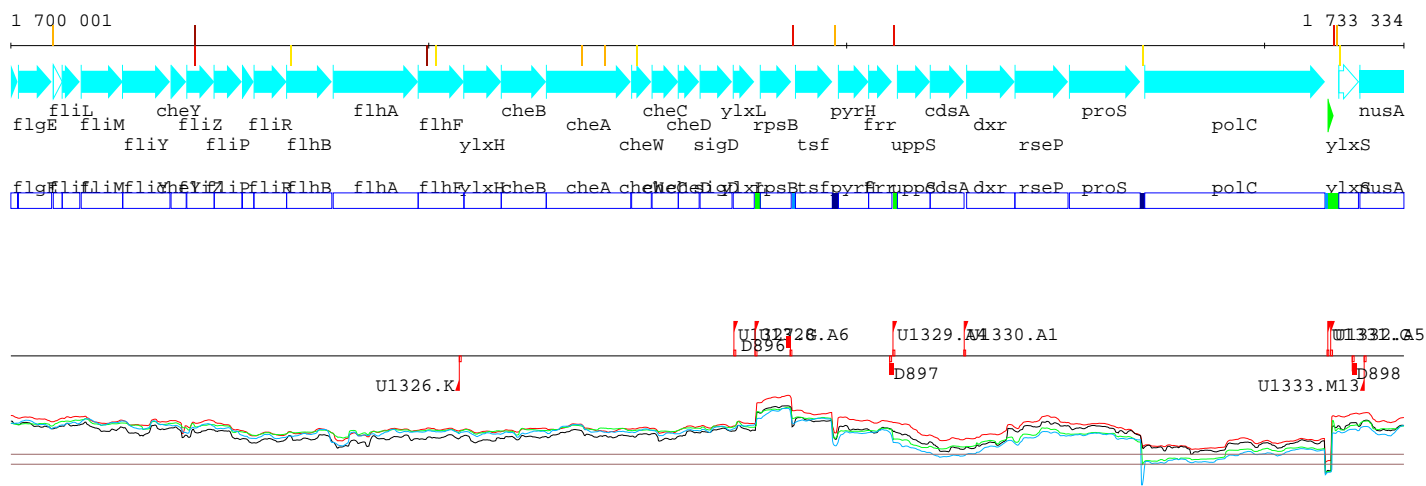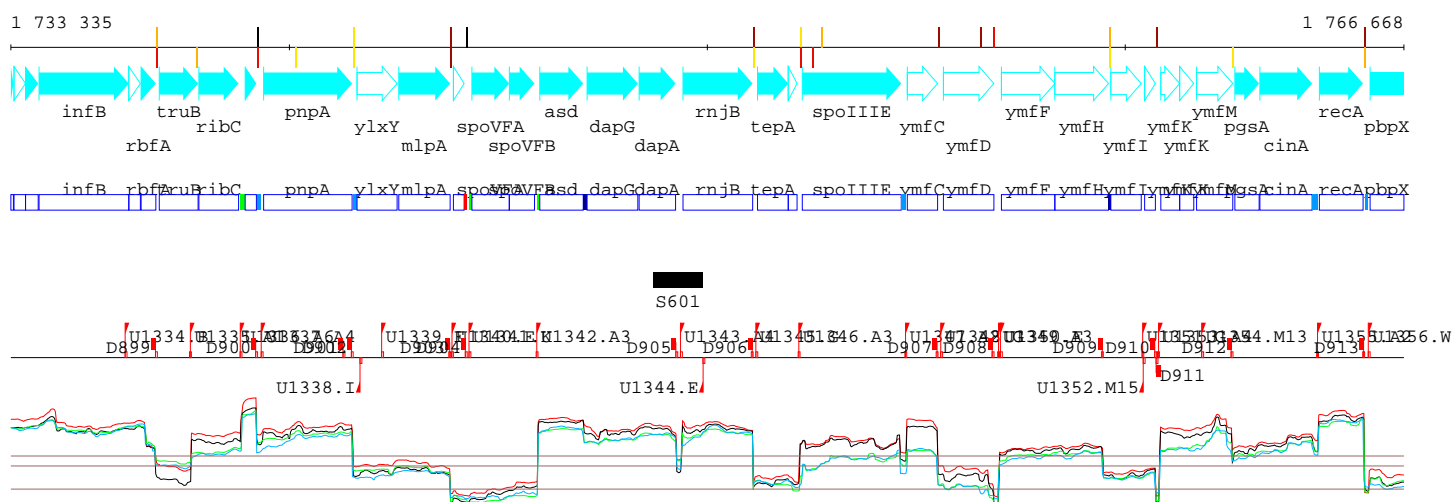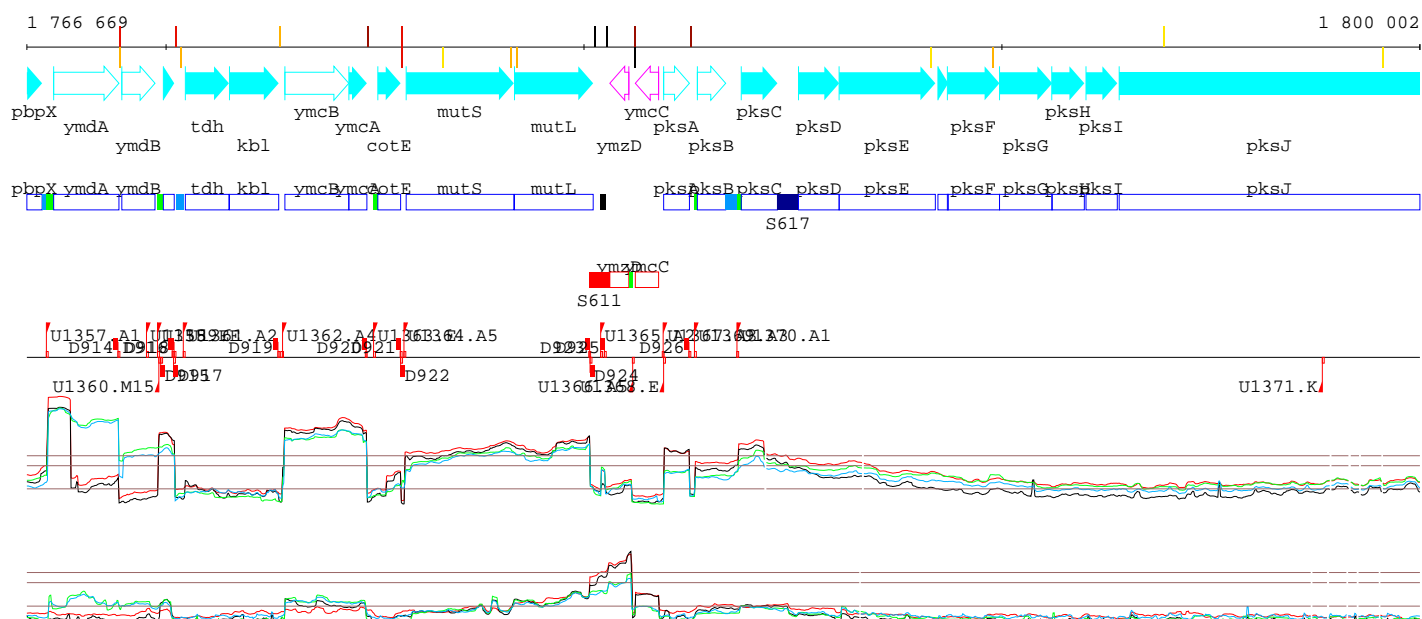

1 800 0011 833 334

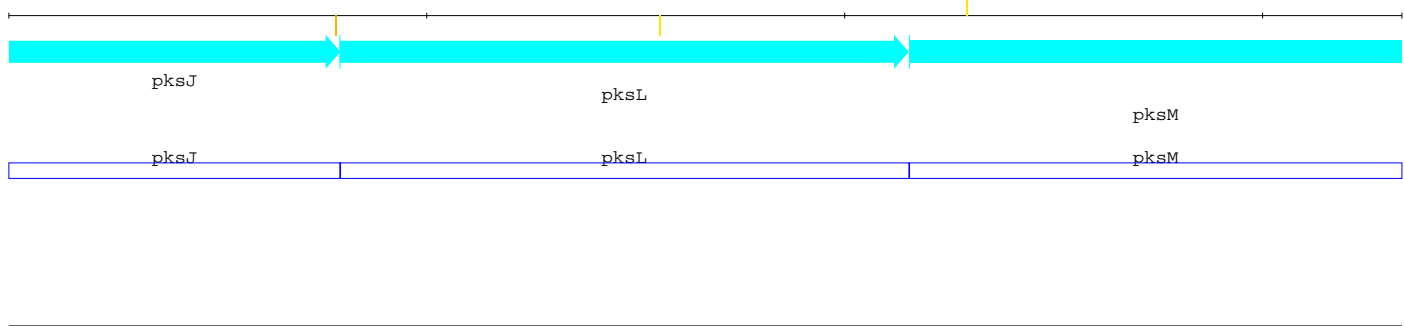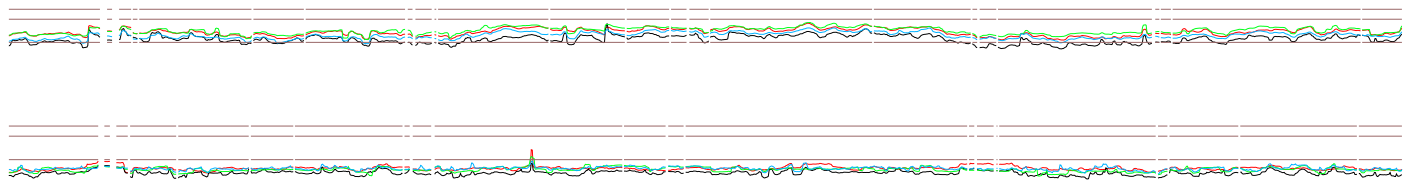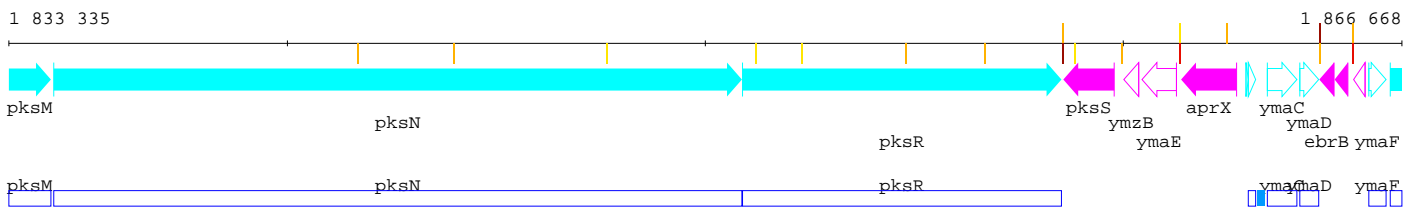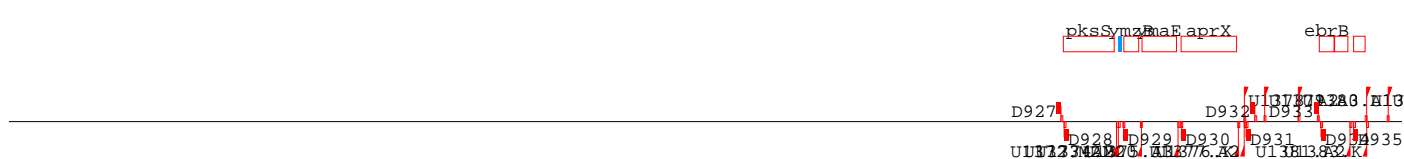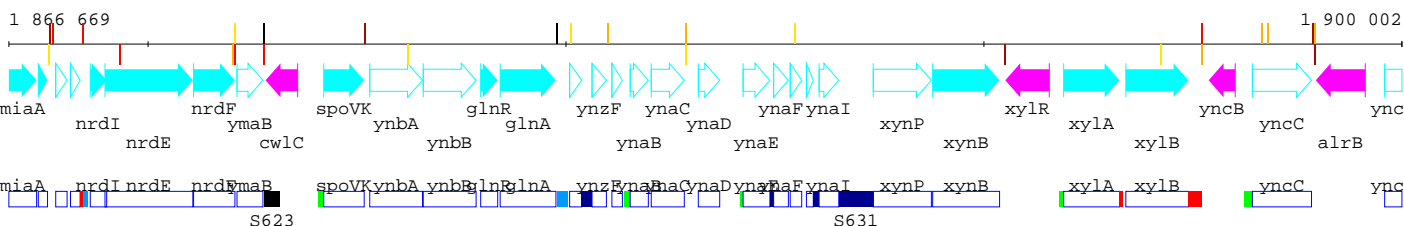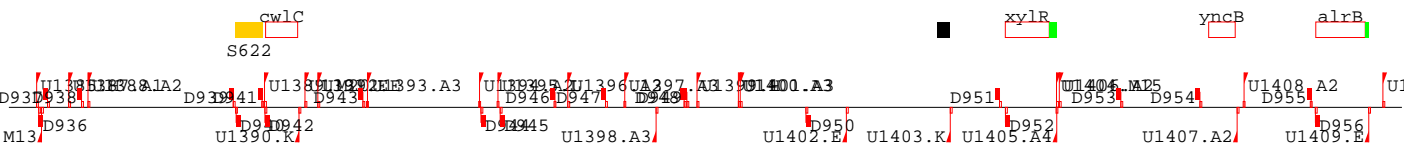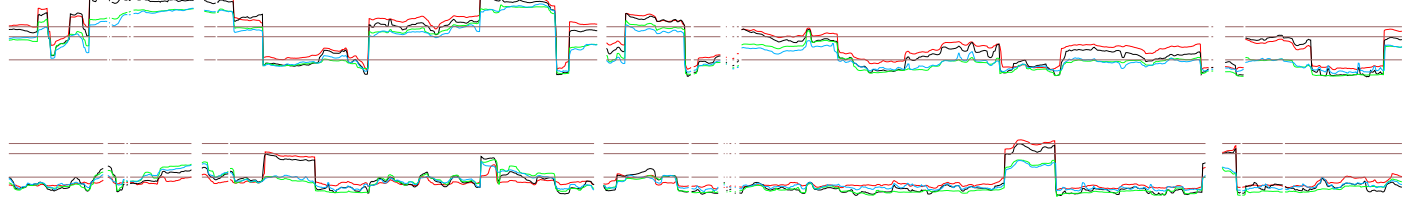

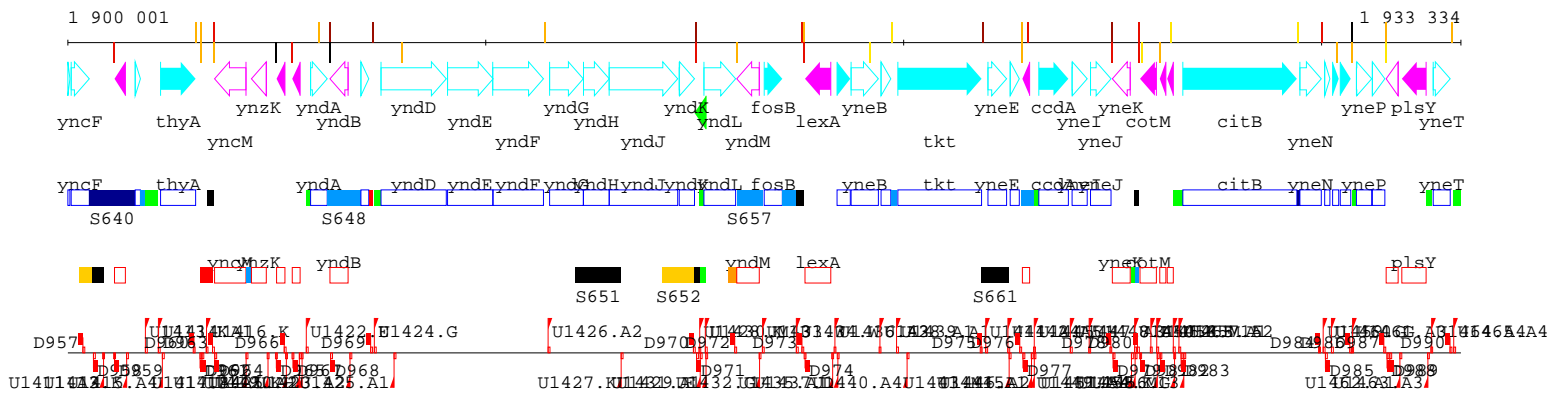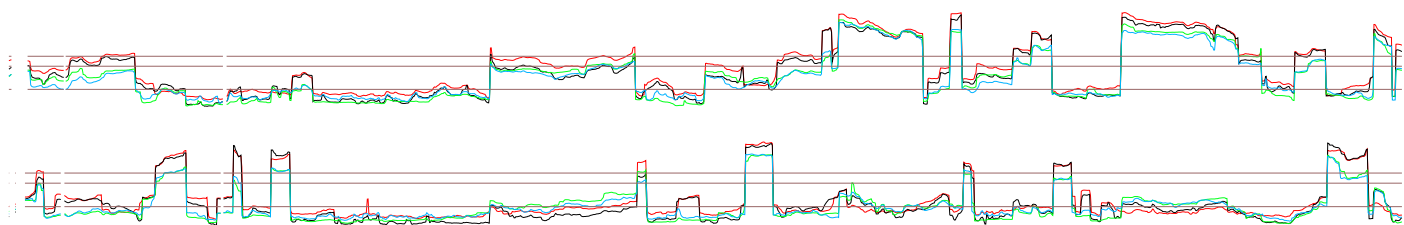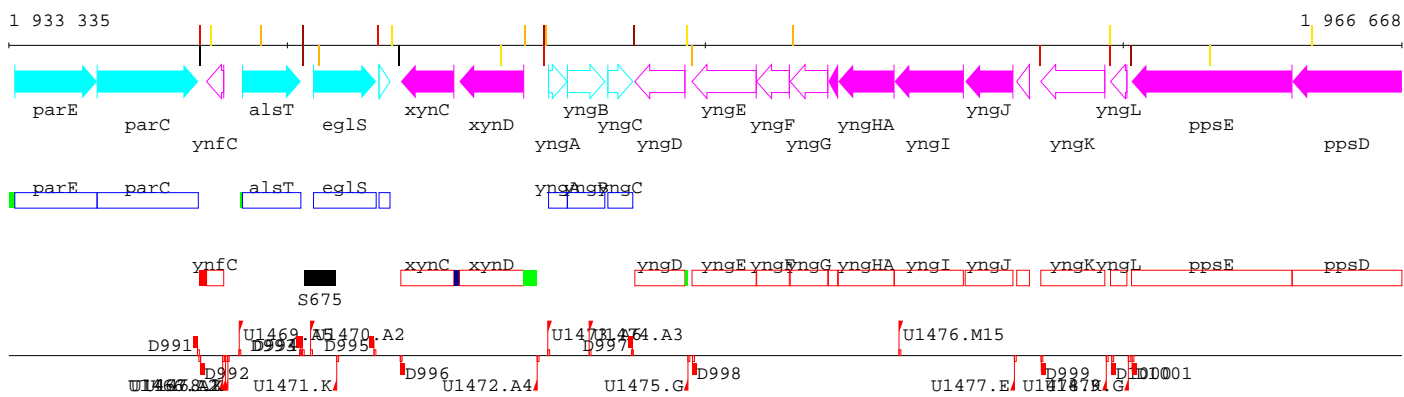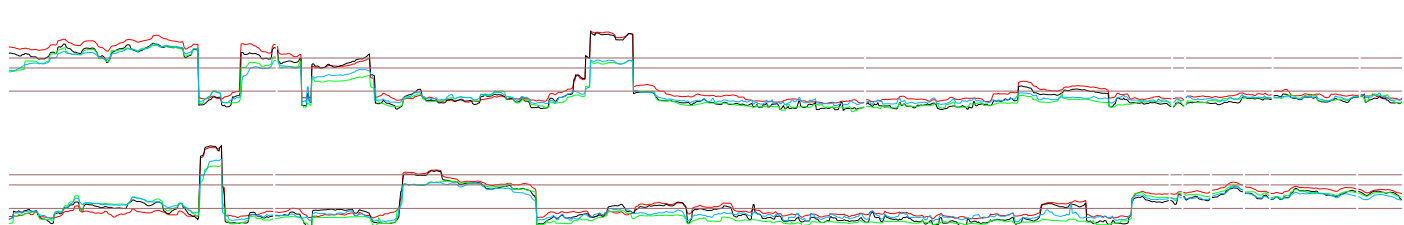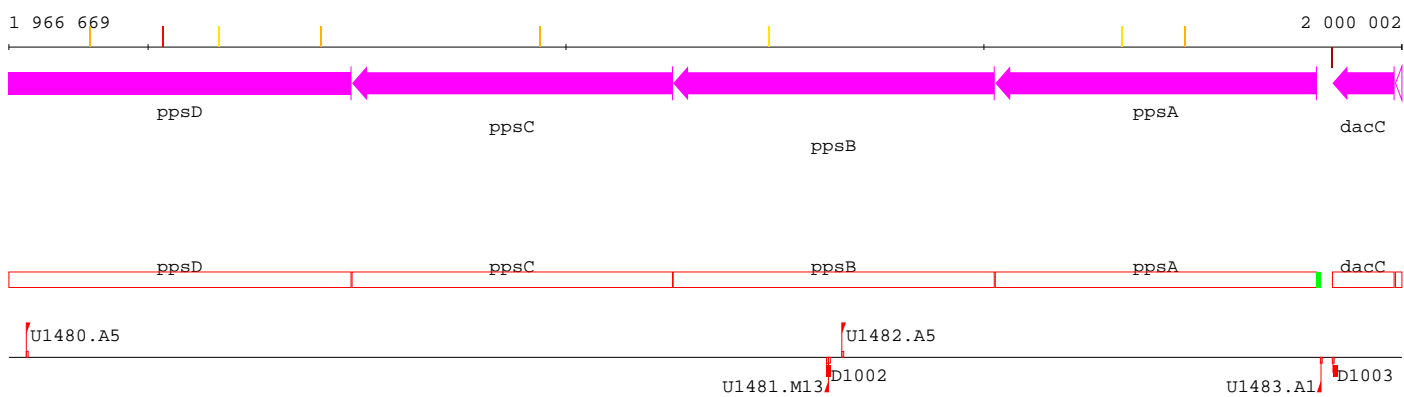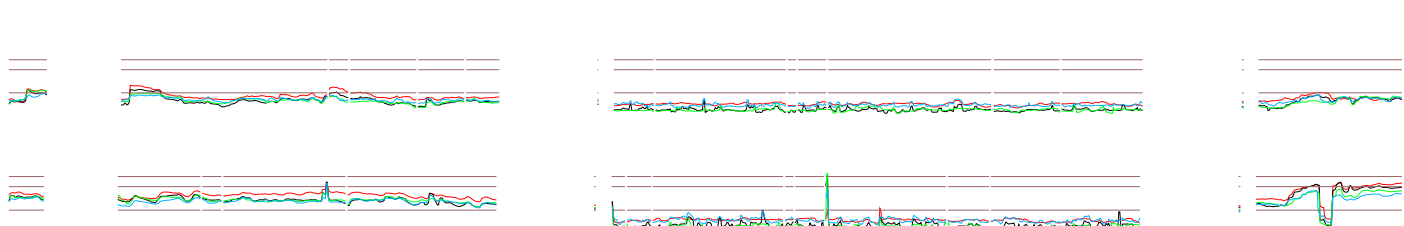

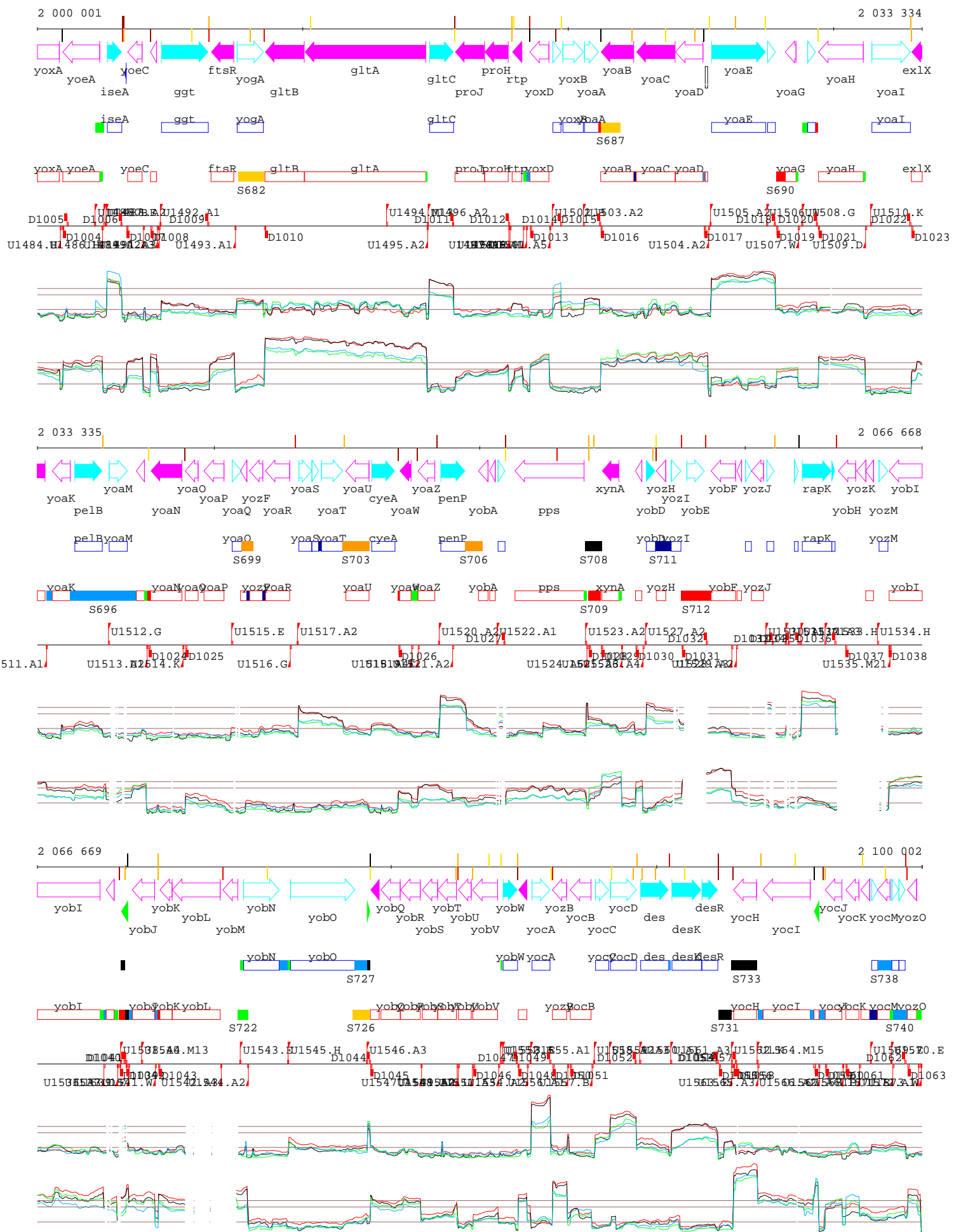

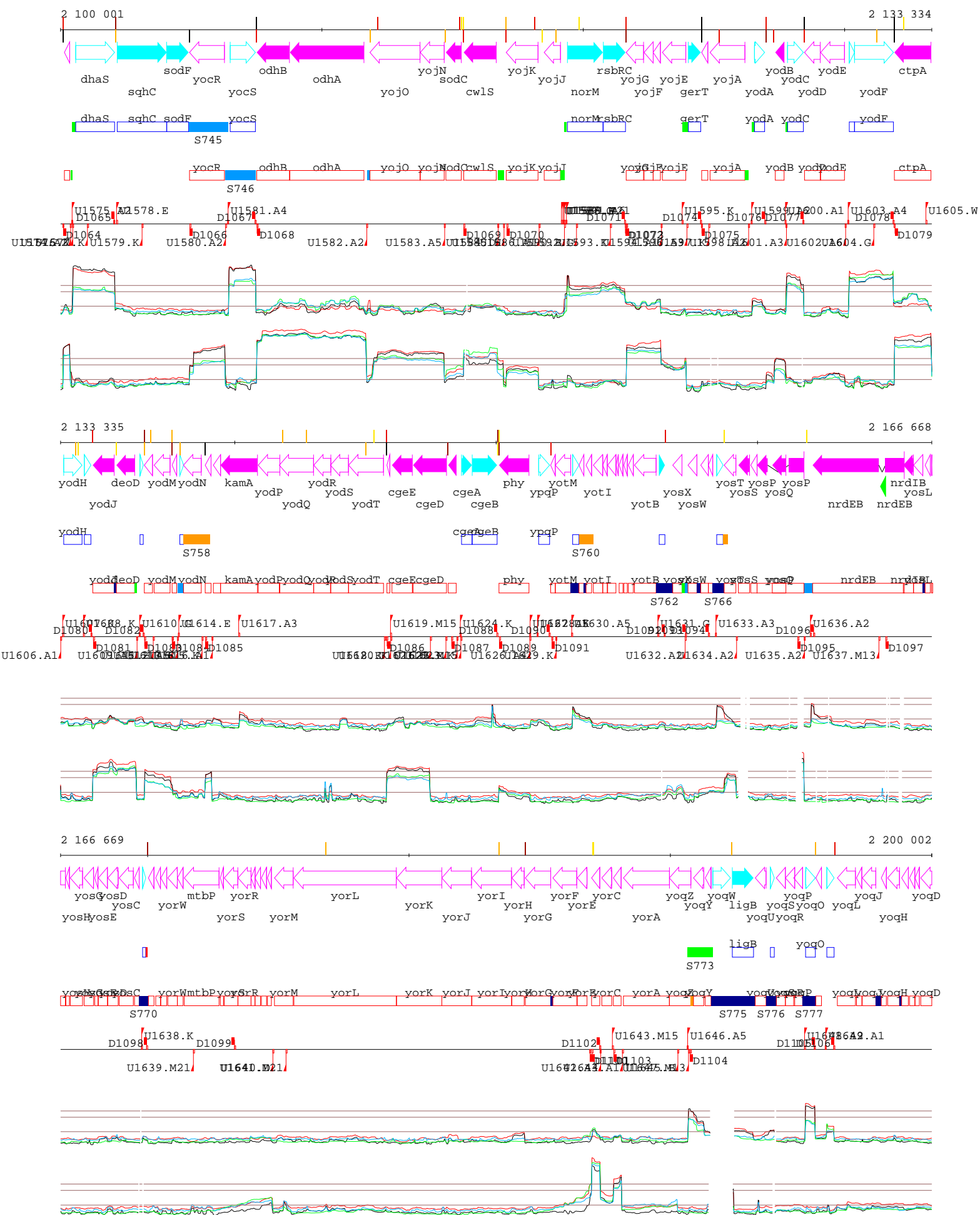

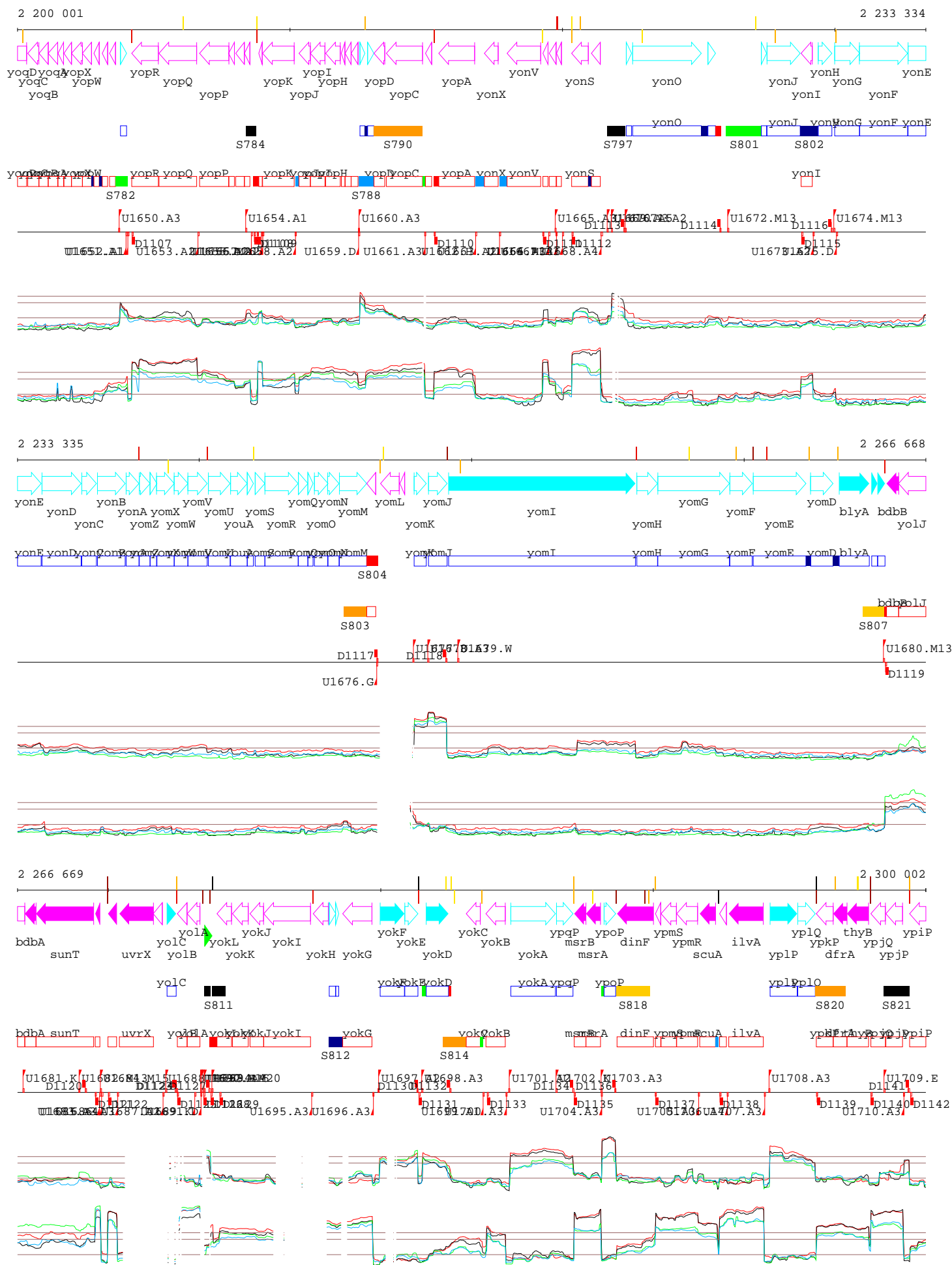



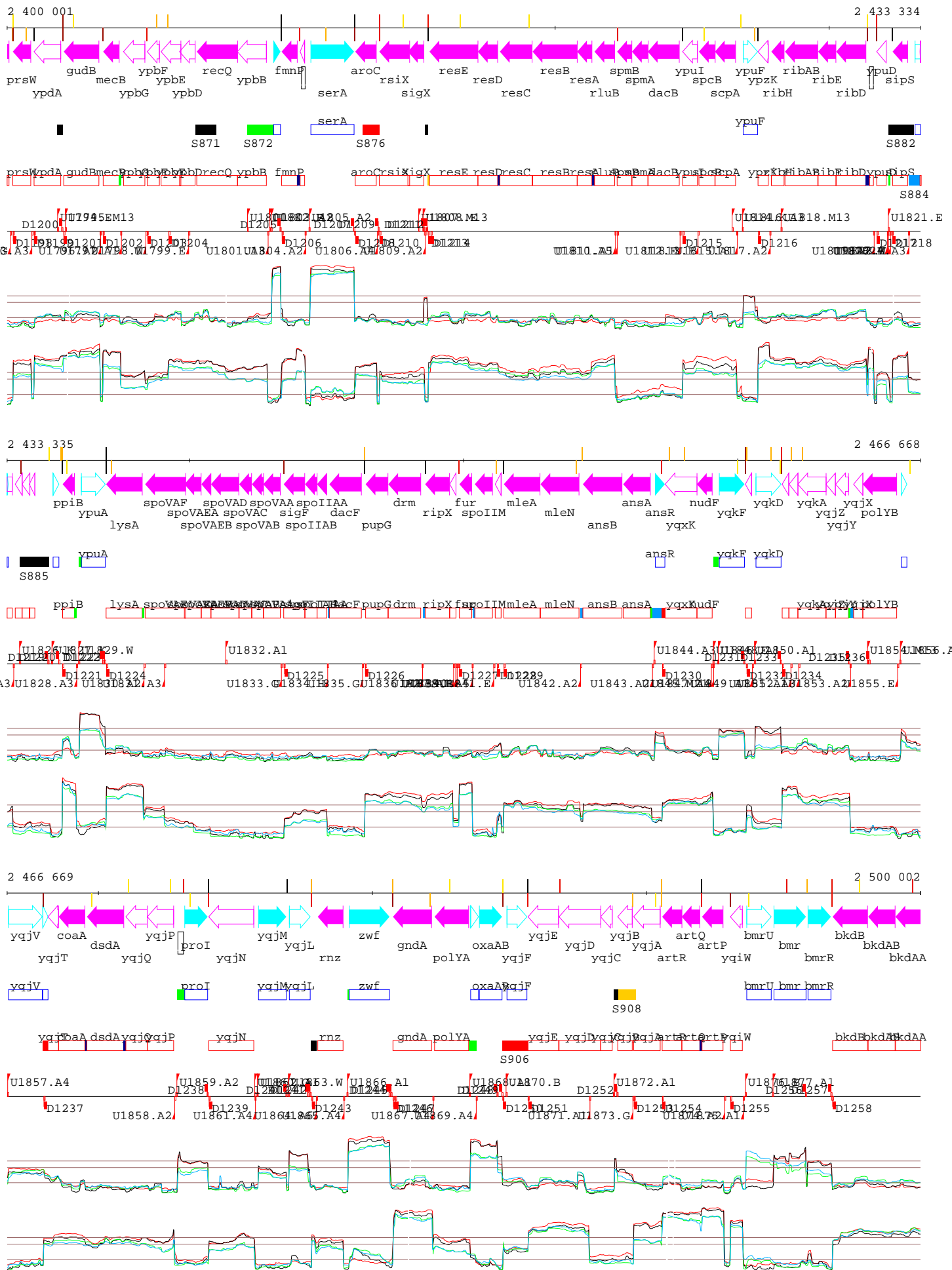

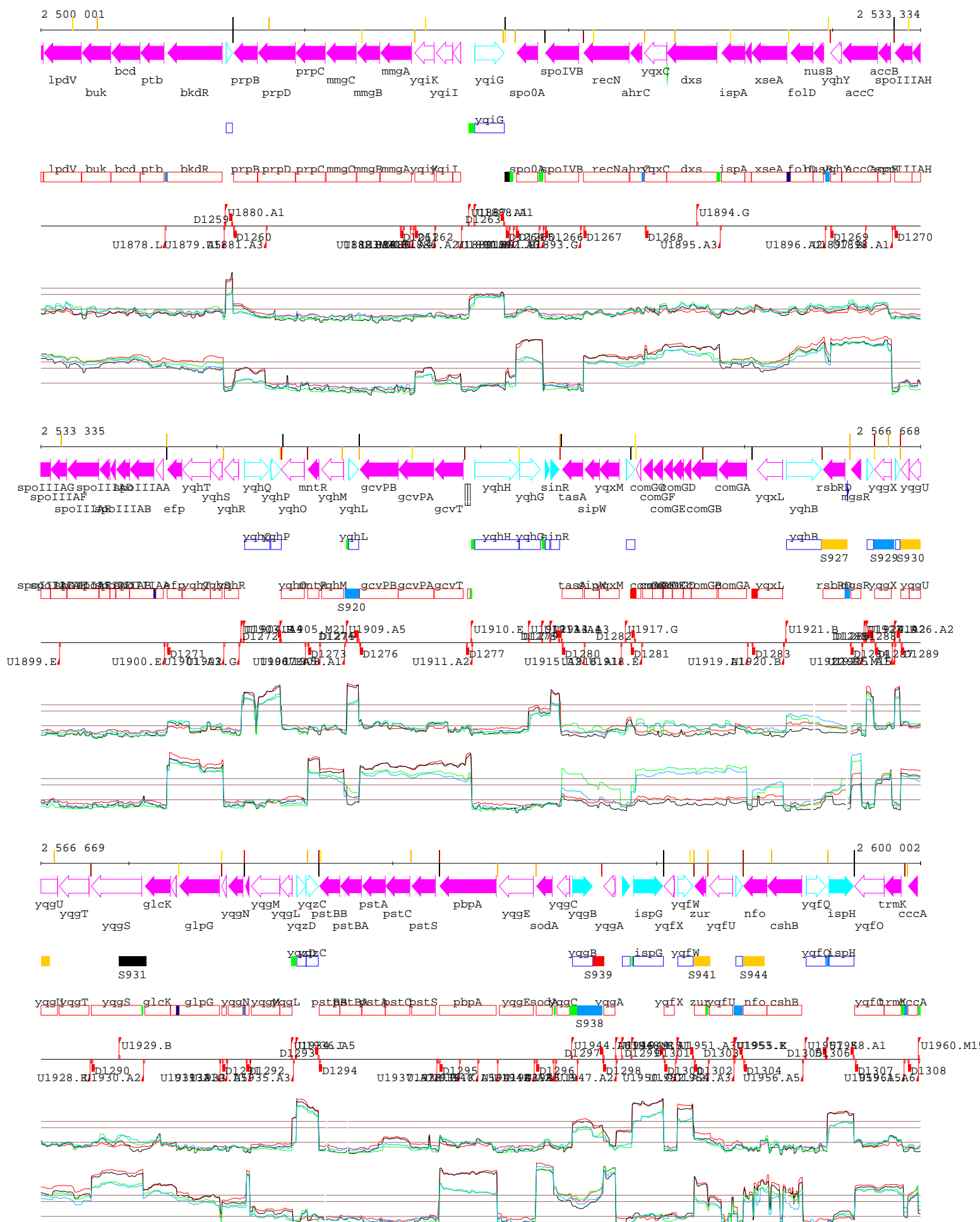

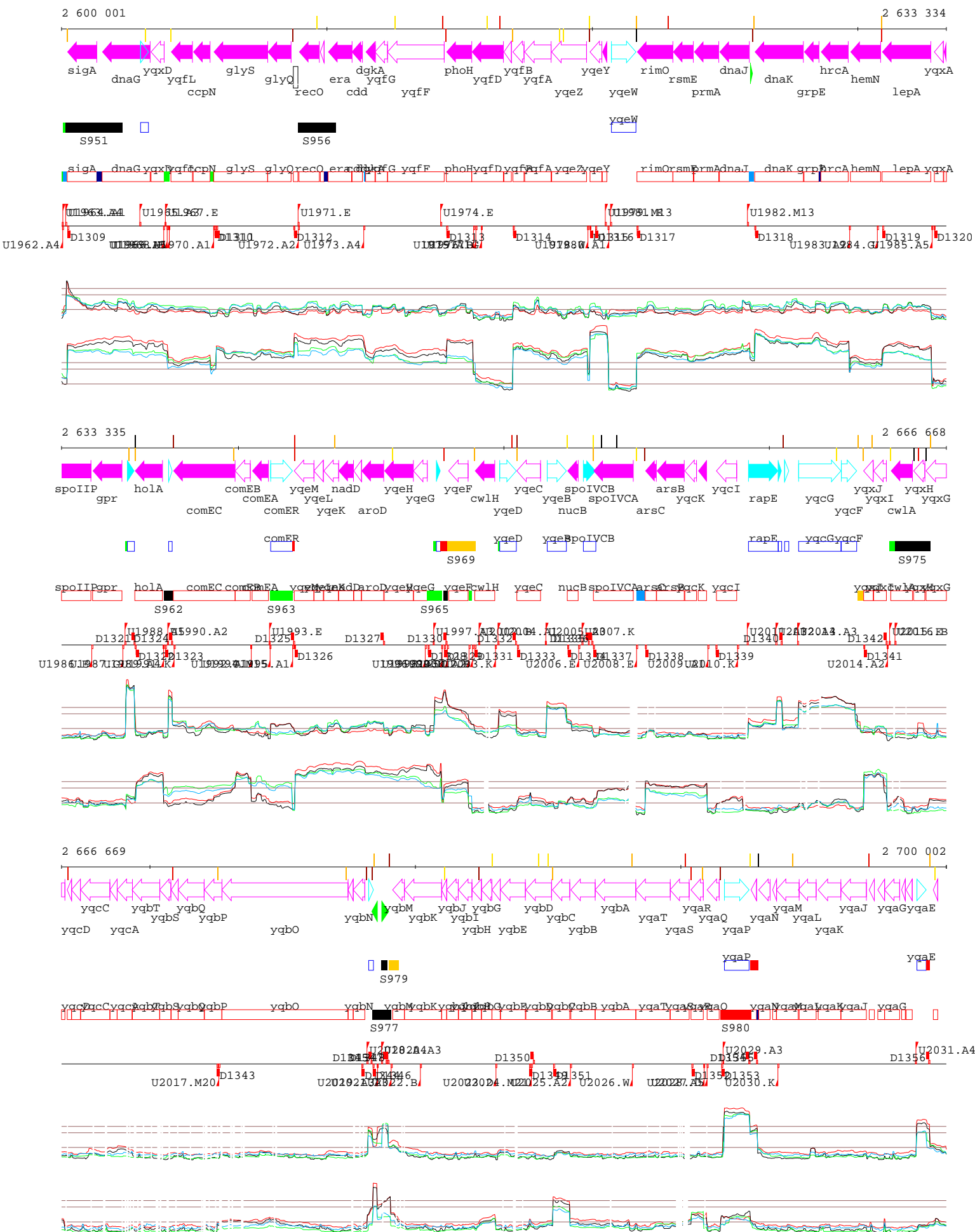



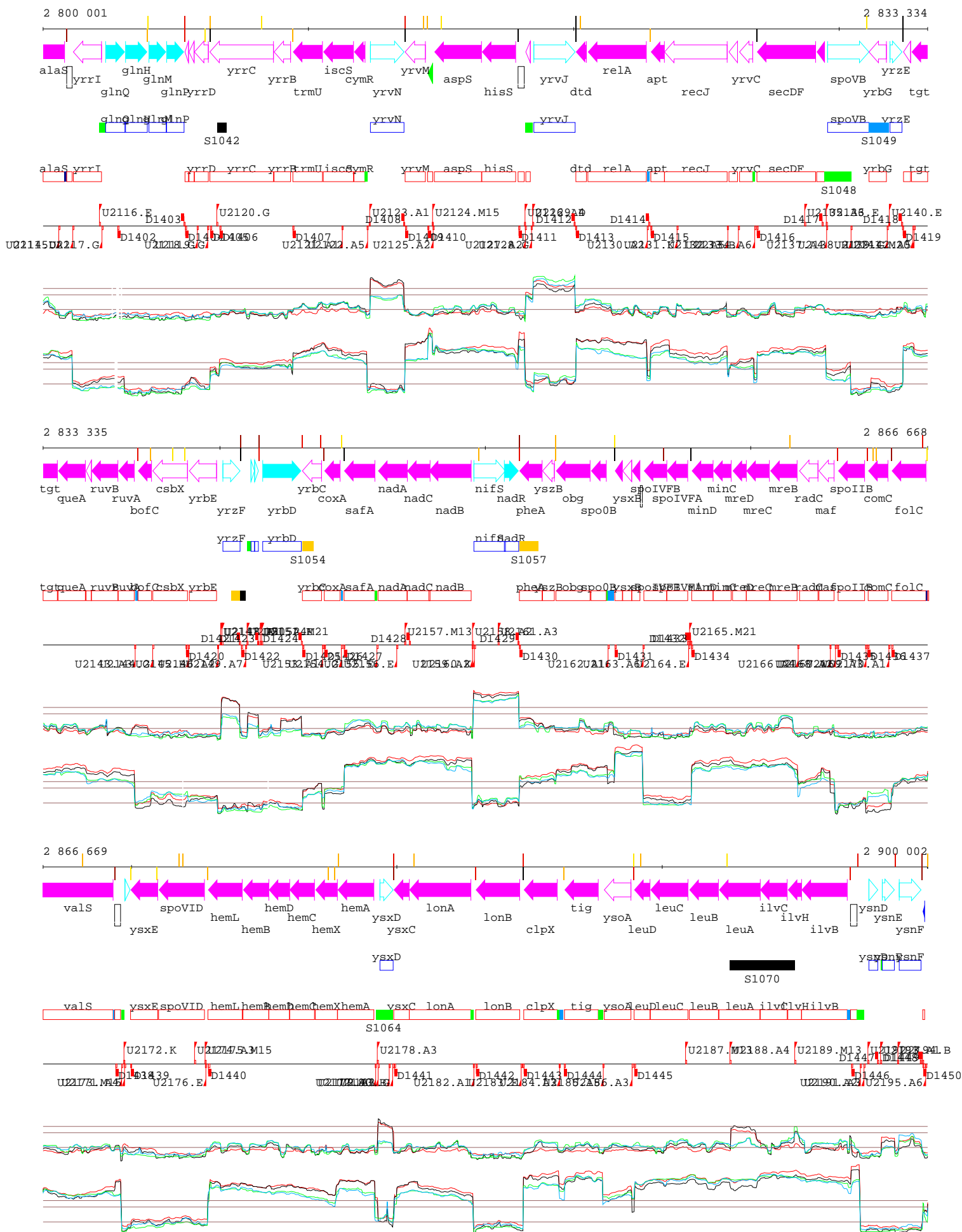



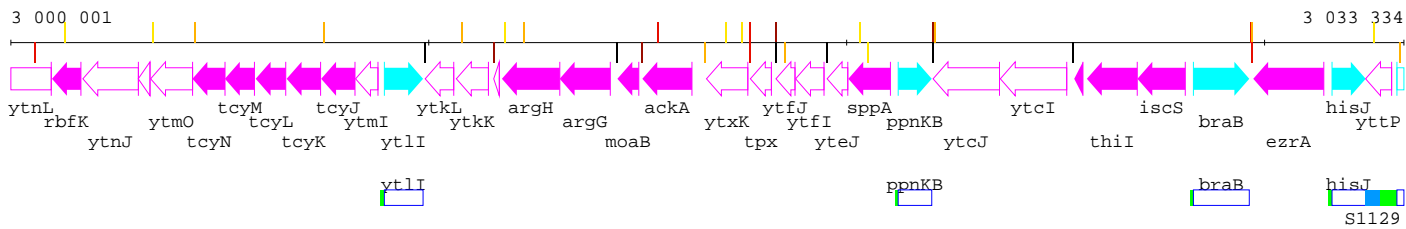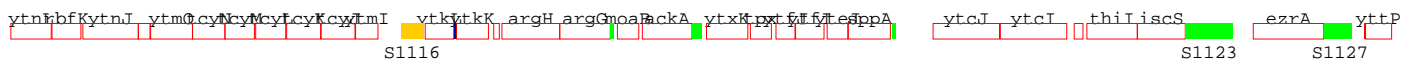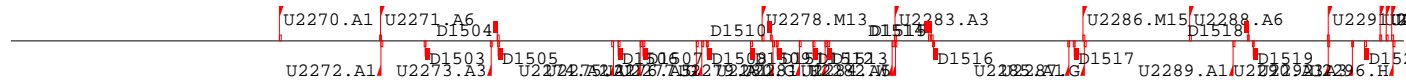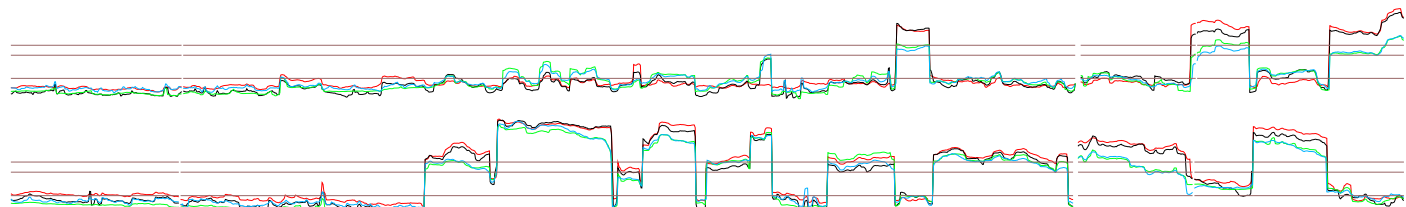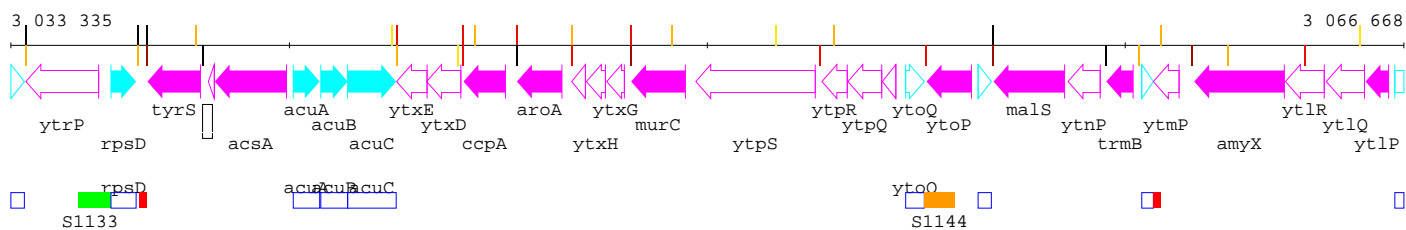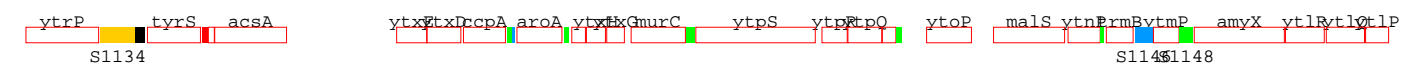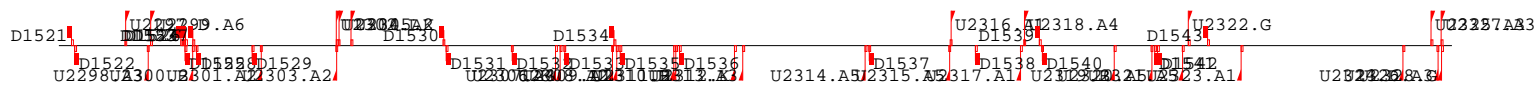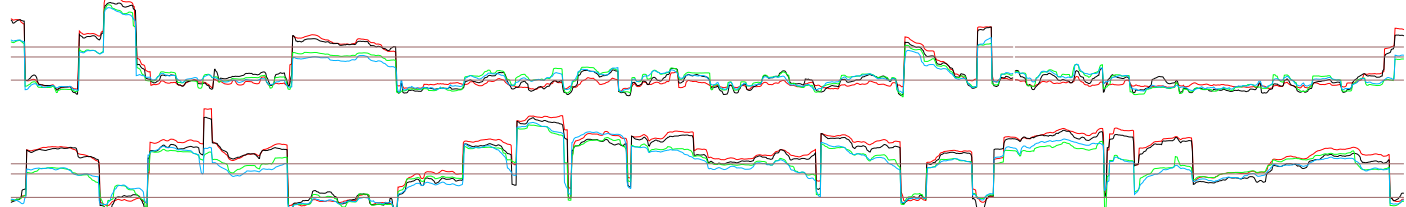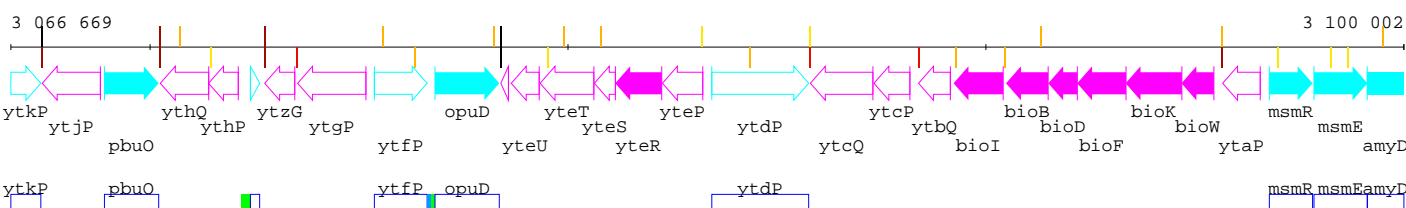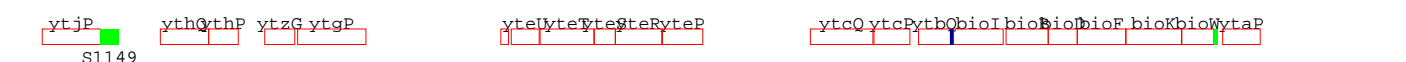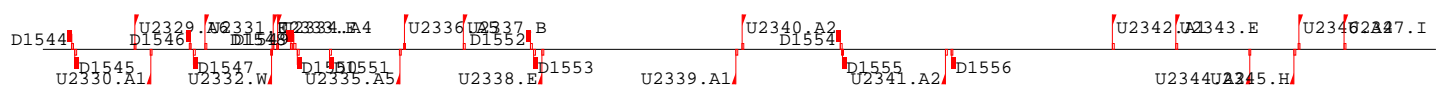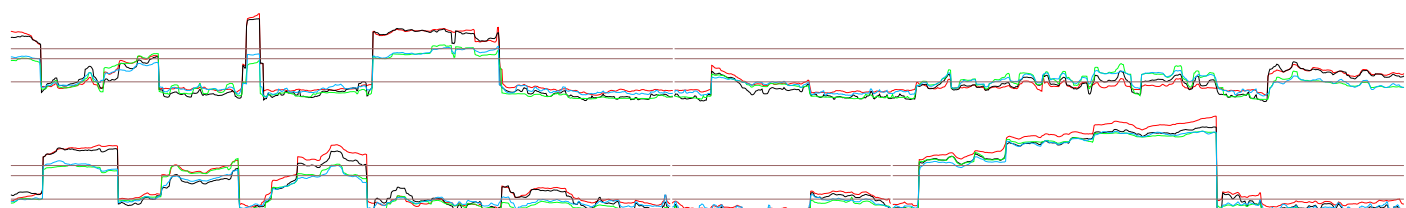

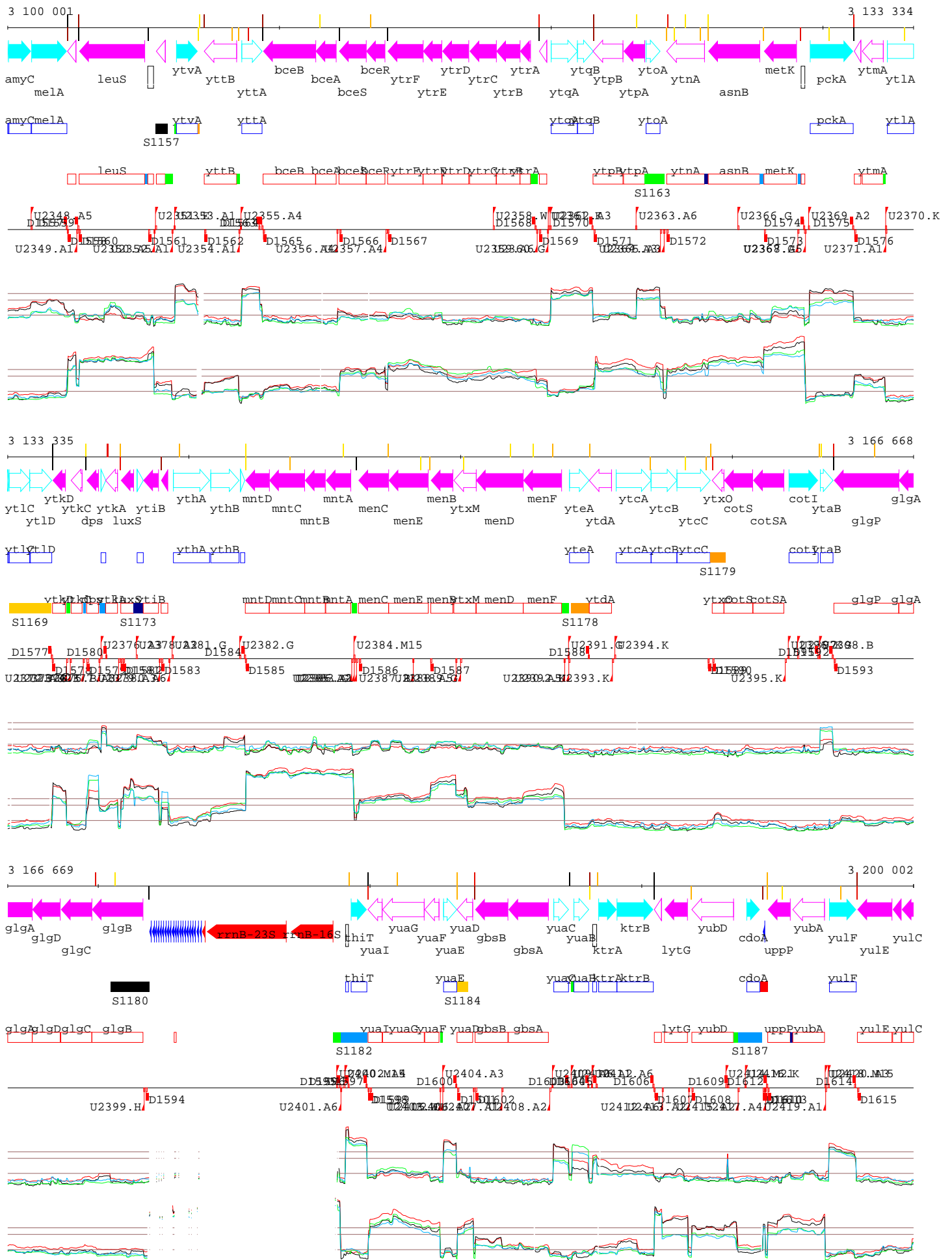



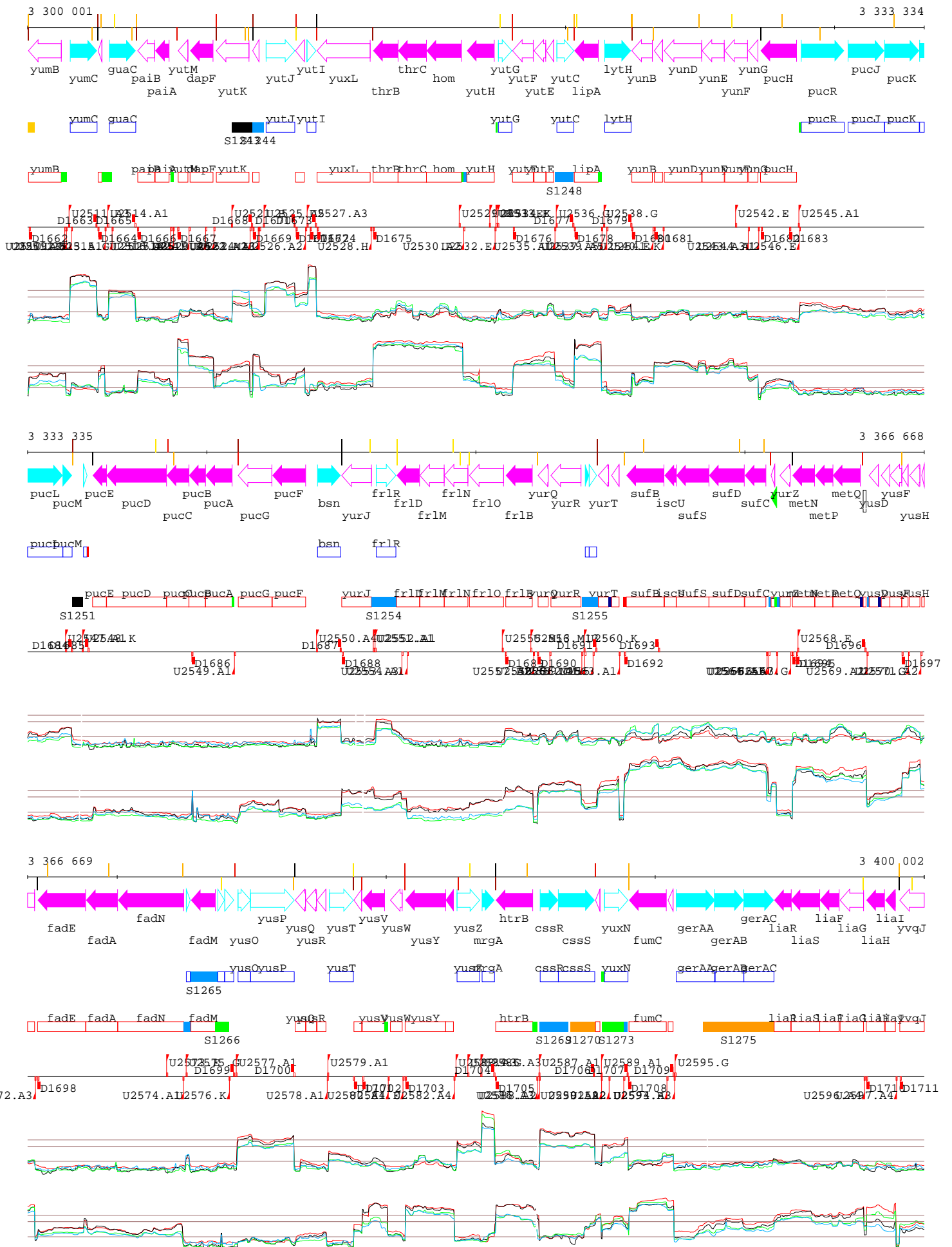

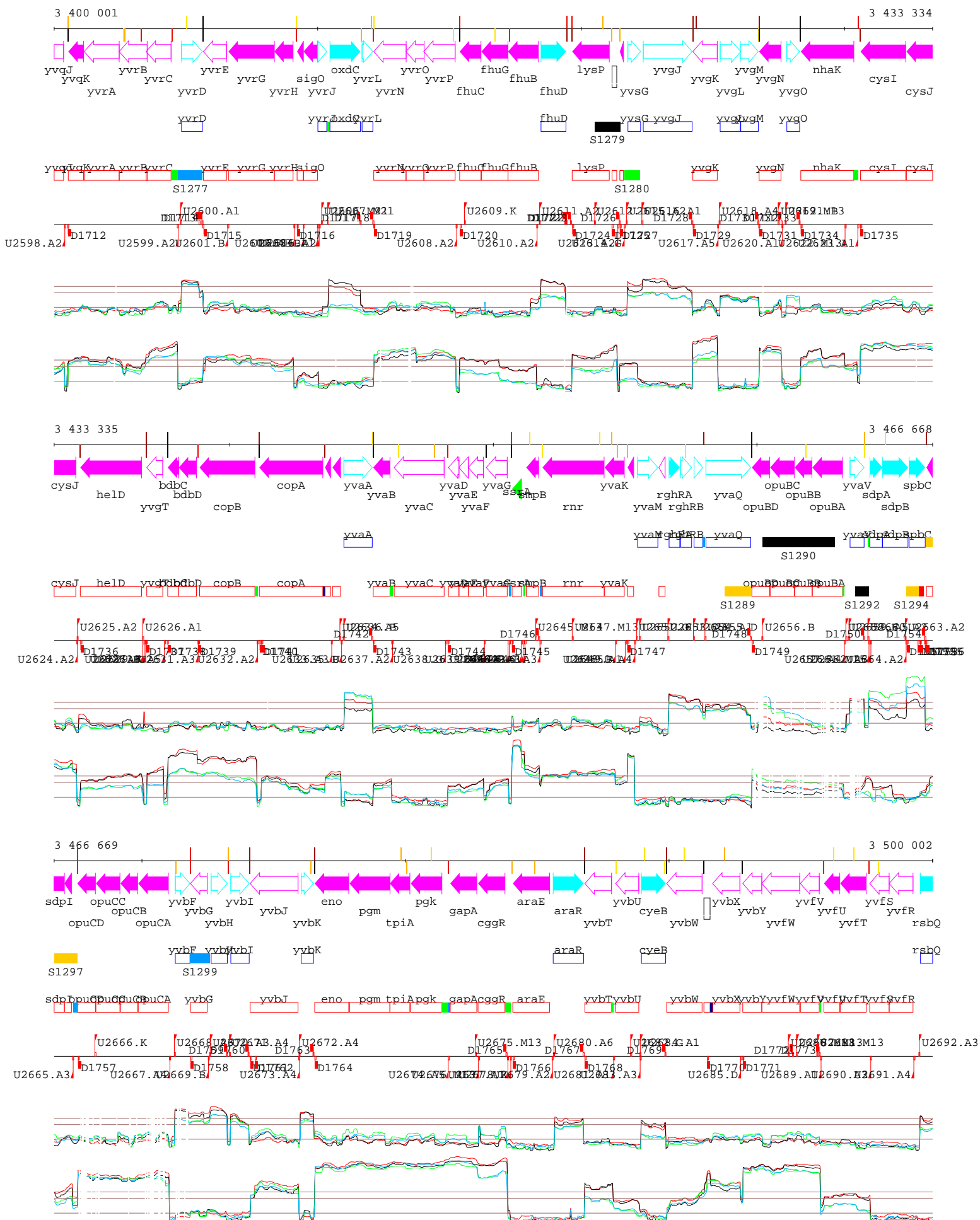

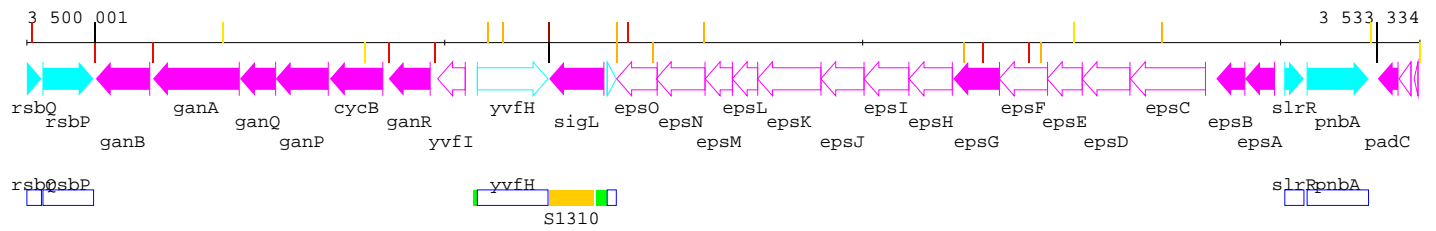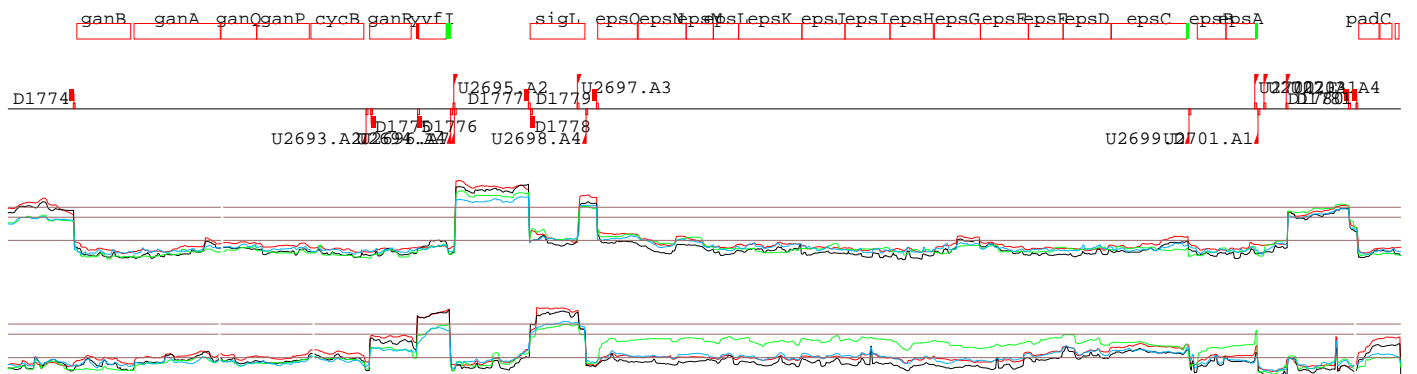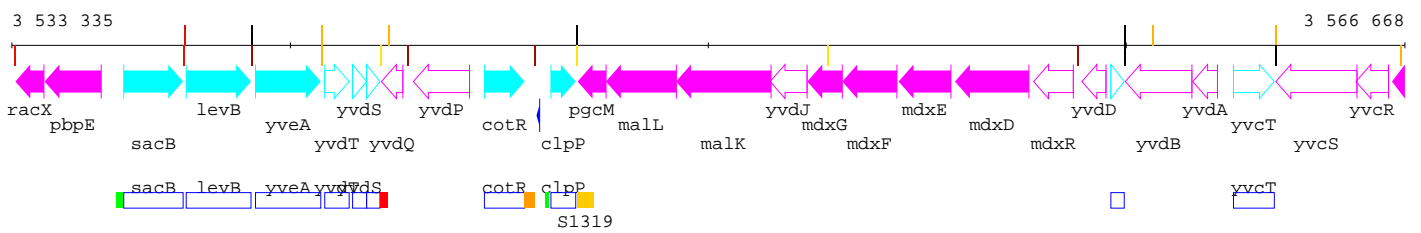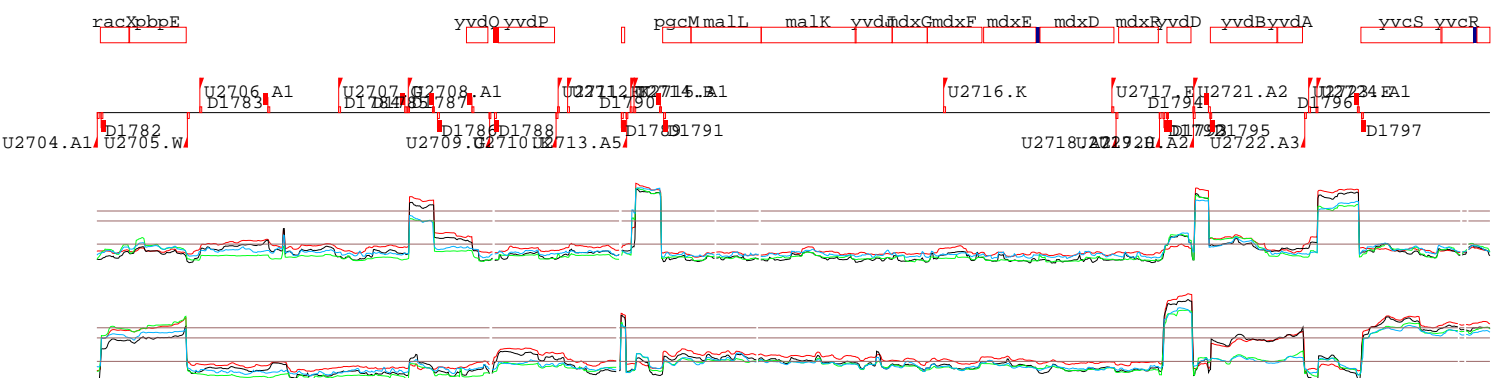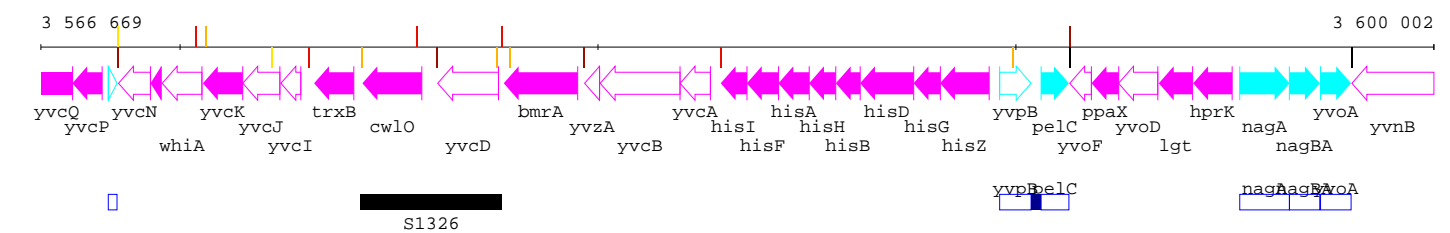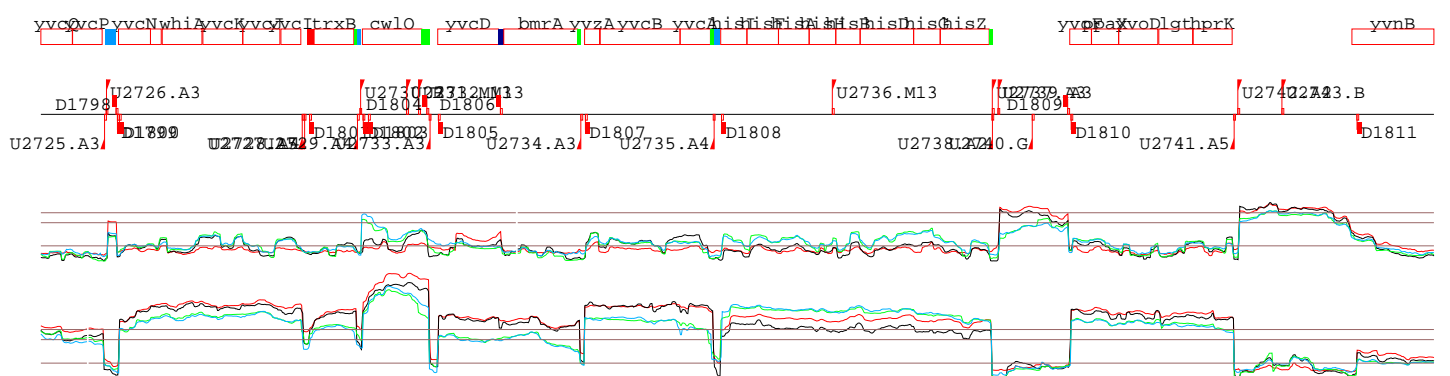

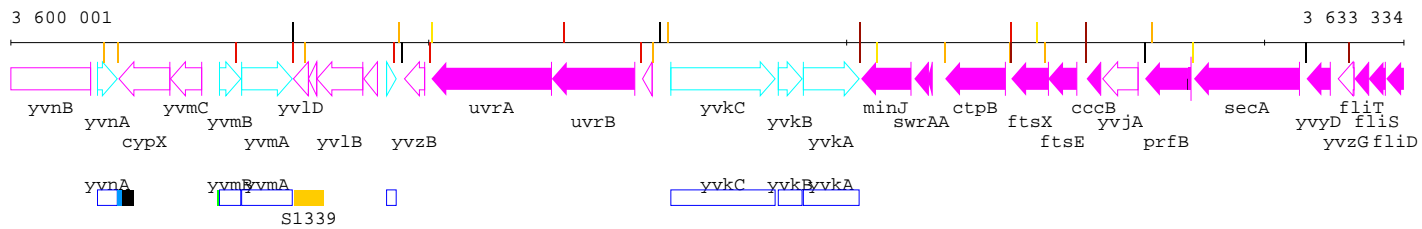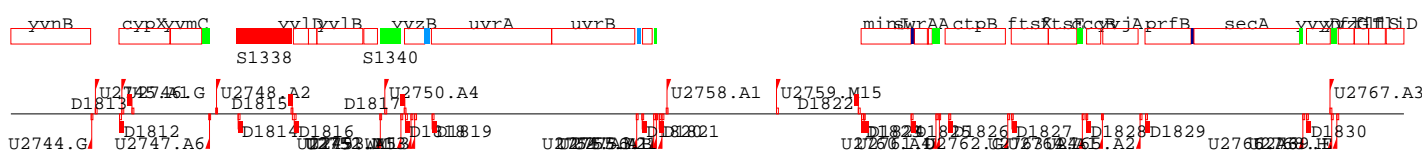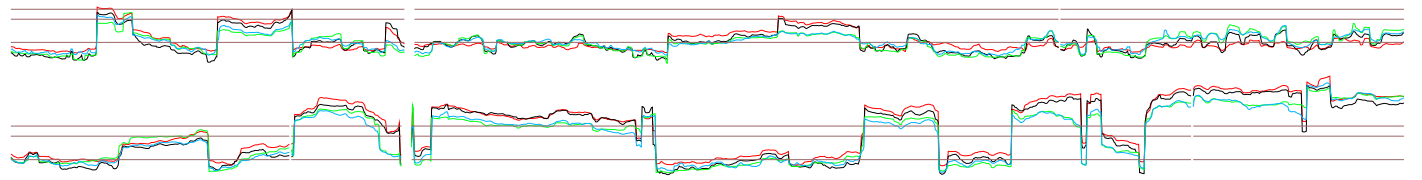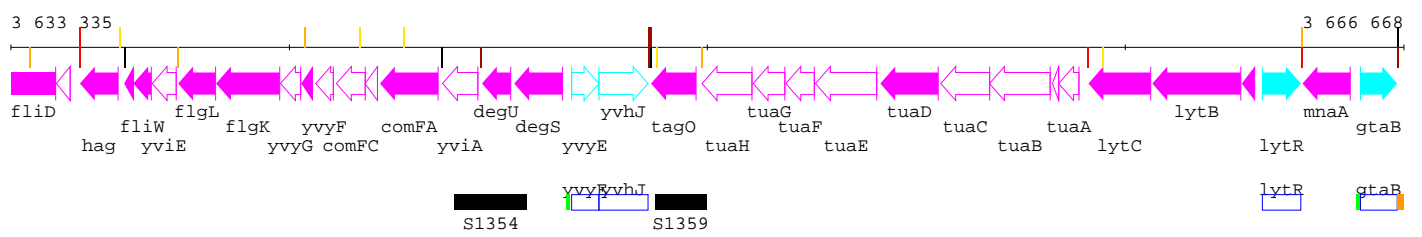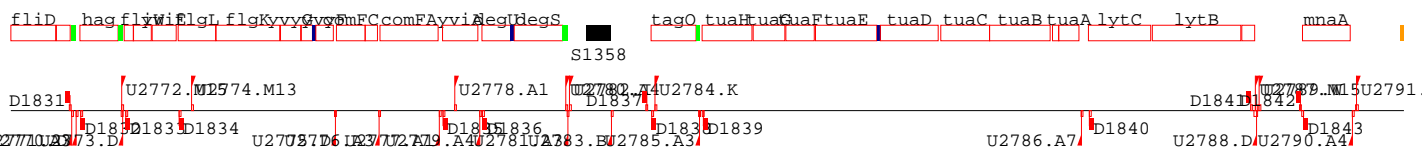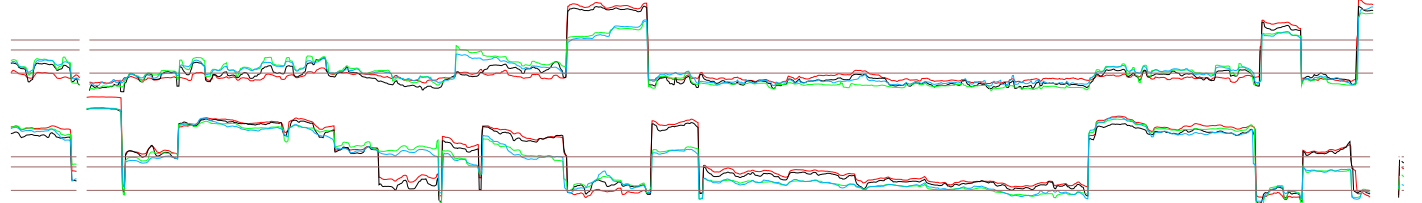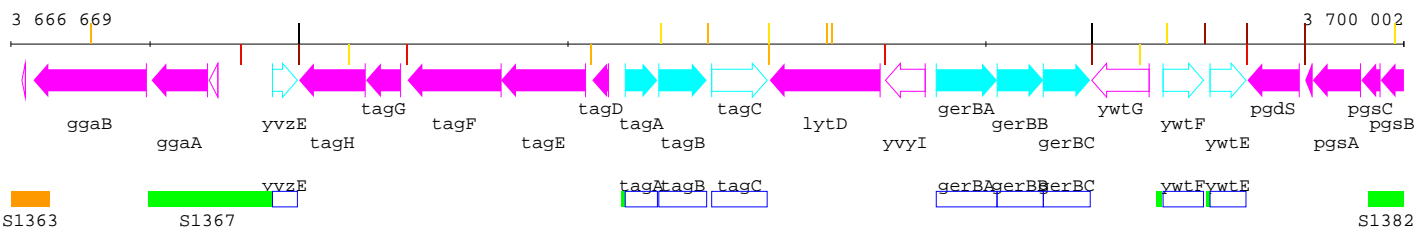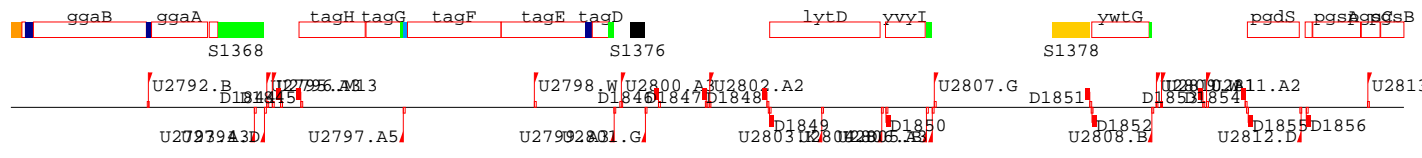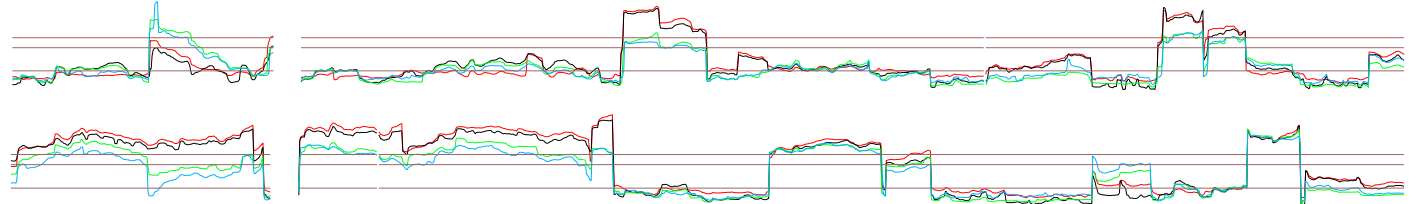

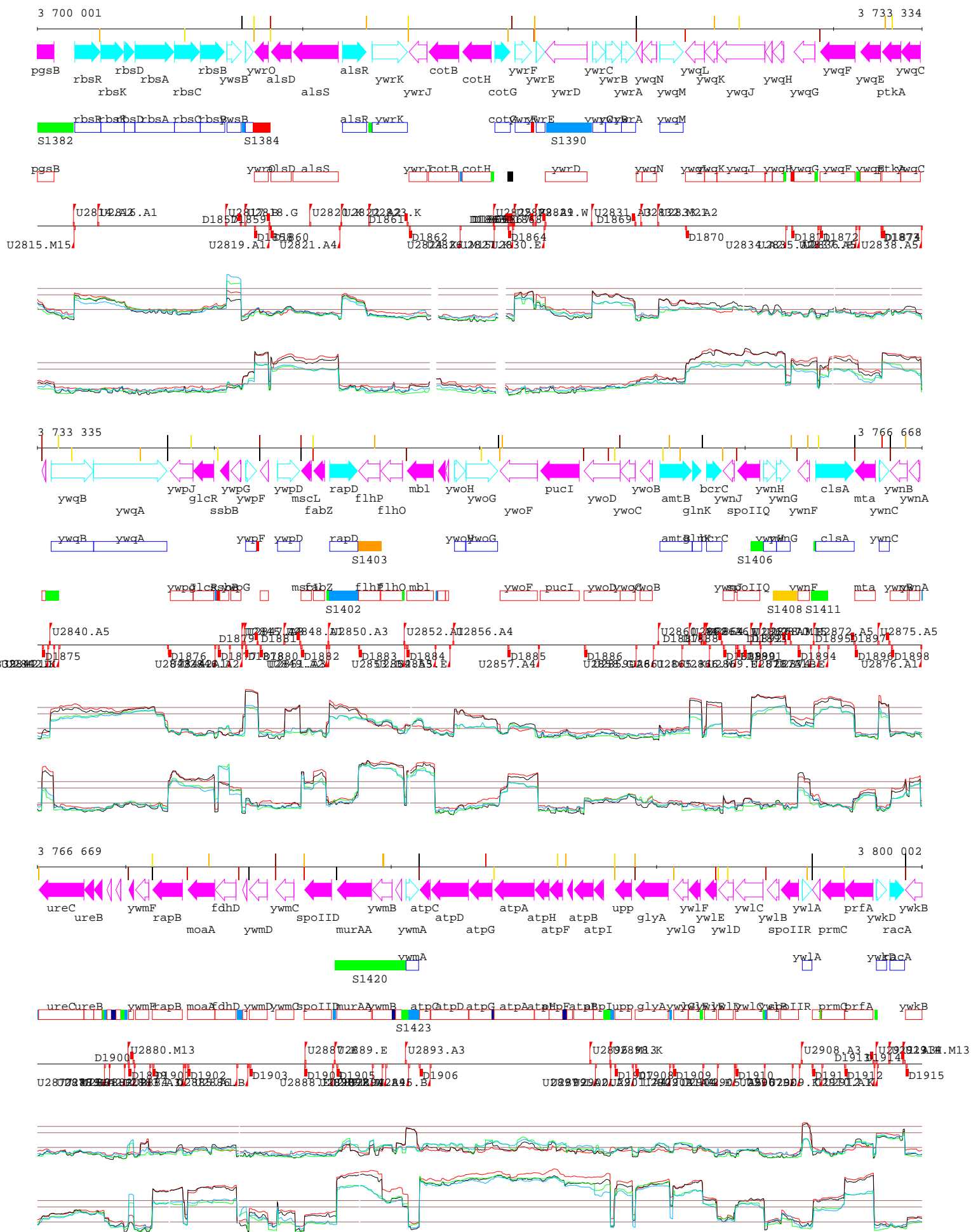

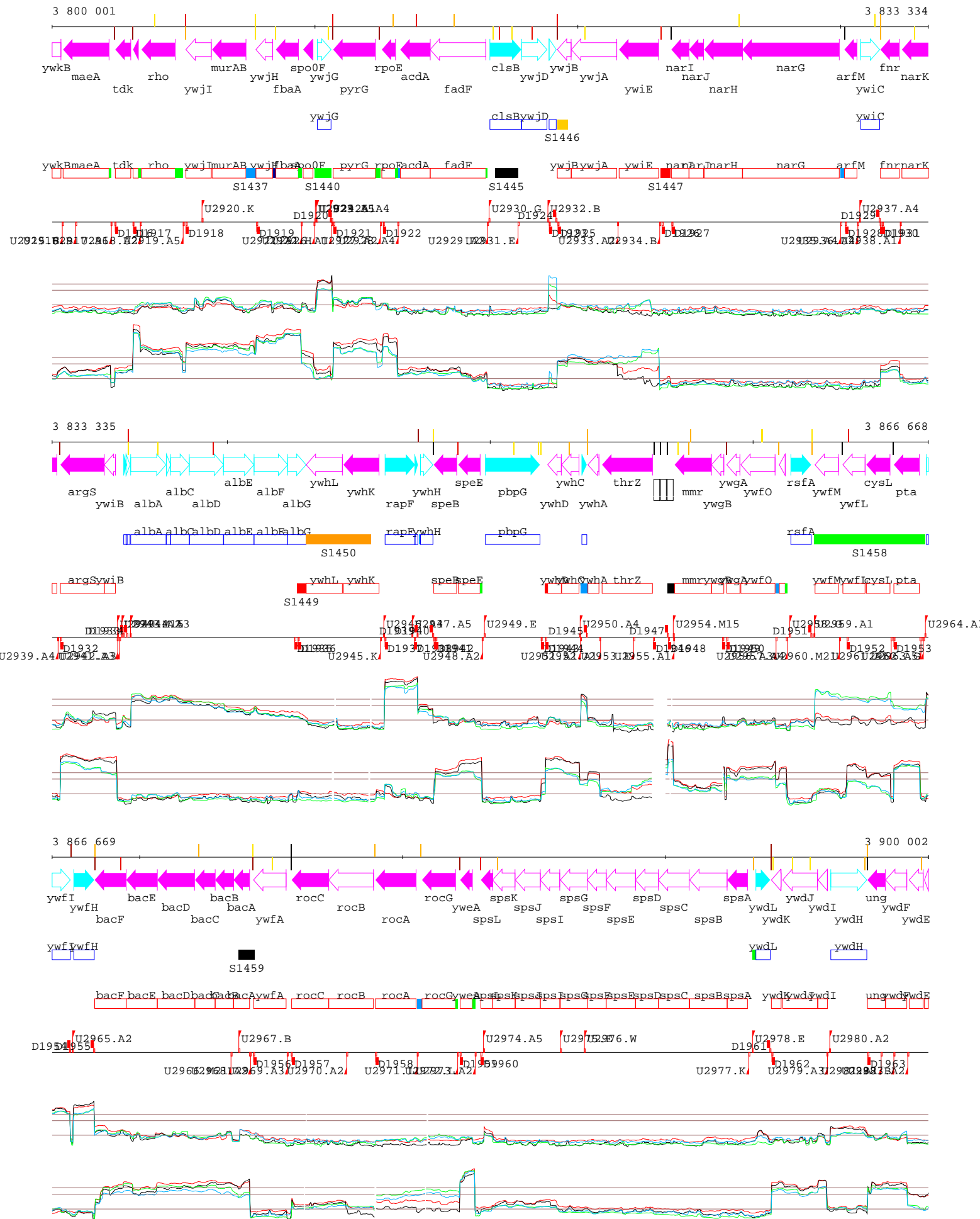

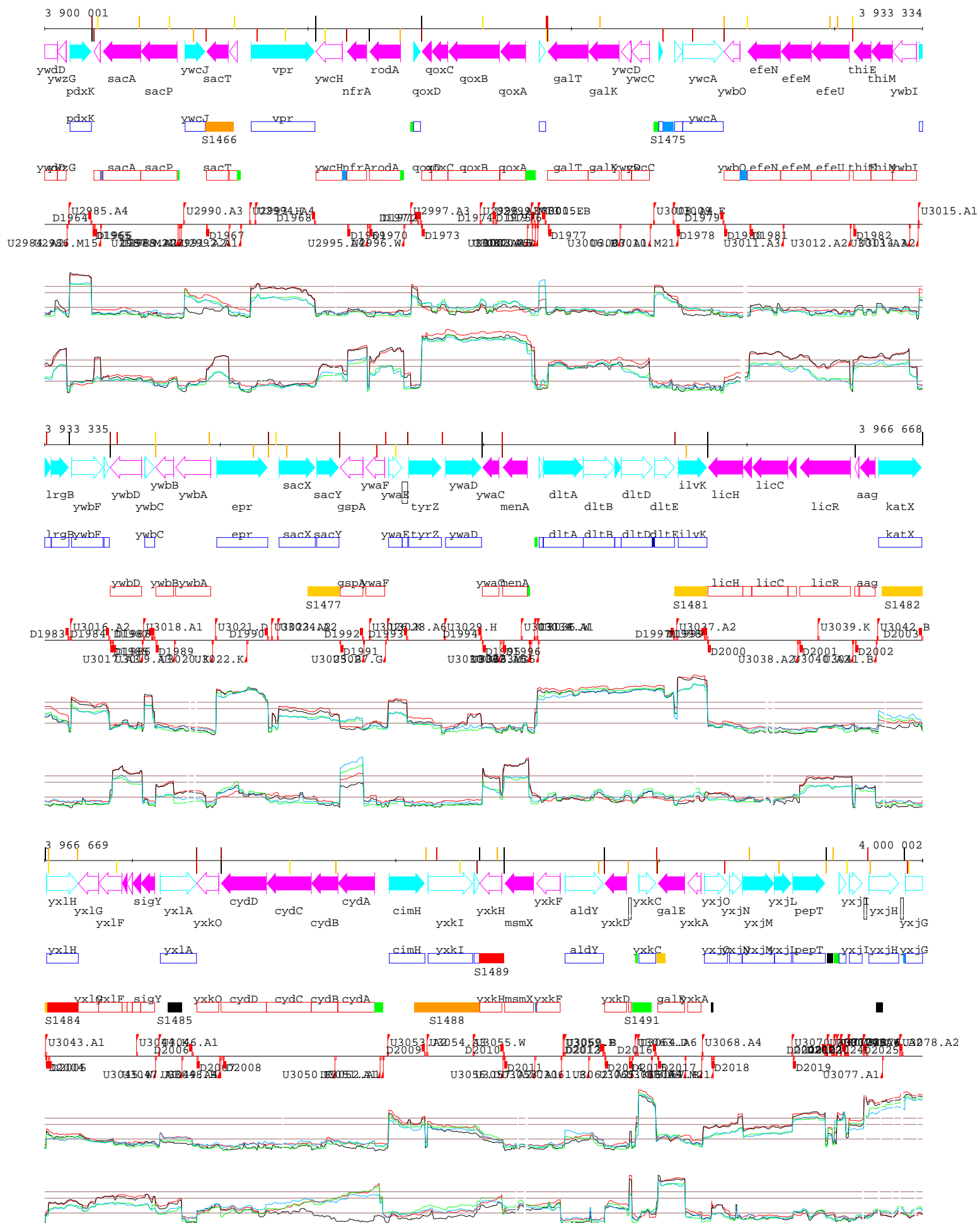

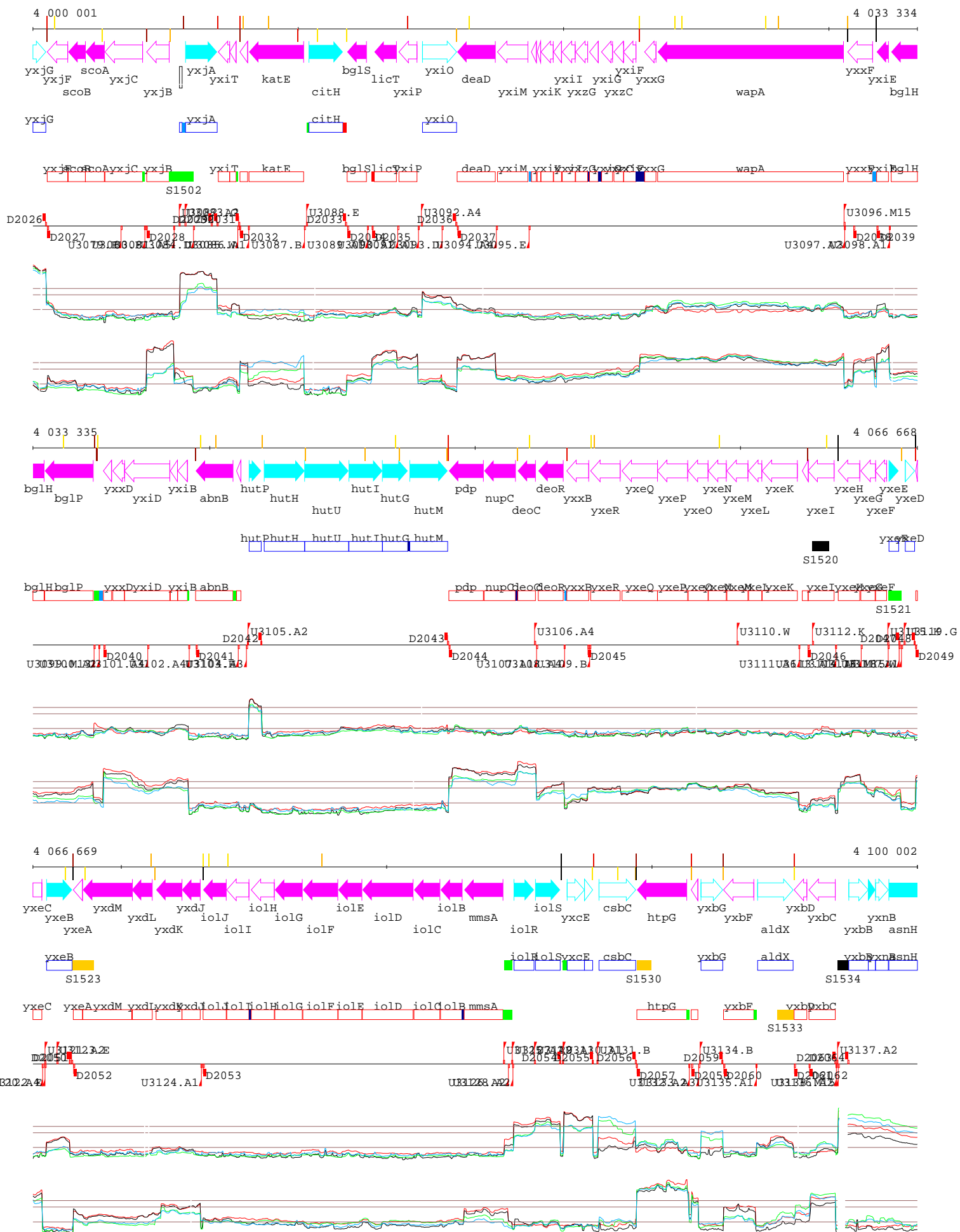



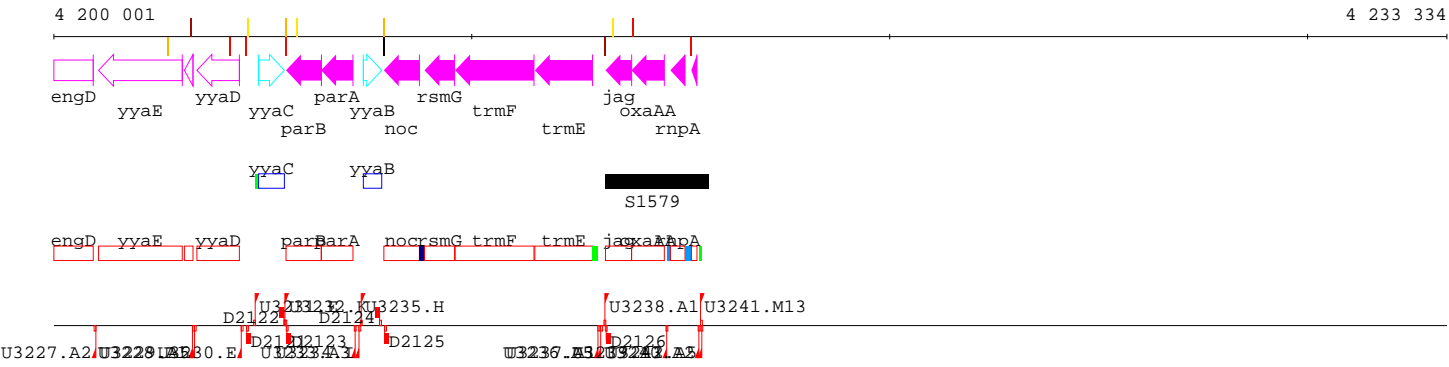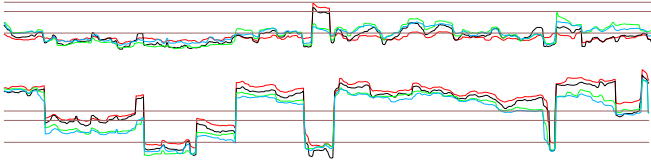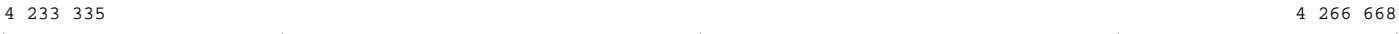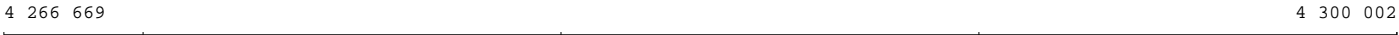

Supplement: Figure S1 — Genome-wide transcriptional landscapes of RNase Y depleted cells. The red/black and blue/green lines show independent duplicate transcription profiles from RNase Y depleted (-IPTG) and RNase Y induced cells (+IPTG). The lower horizontal line represents the global median over the whole chromosome and the two upper lines indicate 5x and 10x this value. Additional information reported here above the transcriptional landscape is from top to bottom: the intrinsic terminators predicted by PETRIN (vertical bars along the x-axis), the genbank annotation (AL009126.3), the new transcription segments and the promoters and terminators identified in Nicolas et al. (2012). (PDF) [file pone.0054062.s001.pdf]

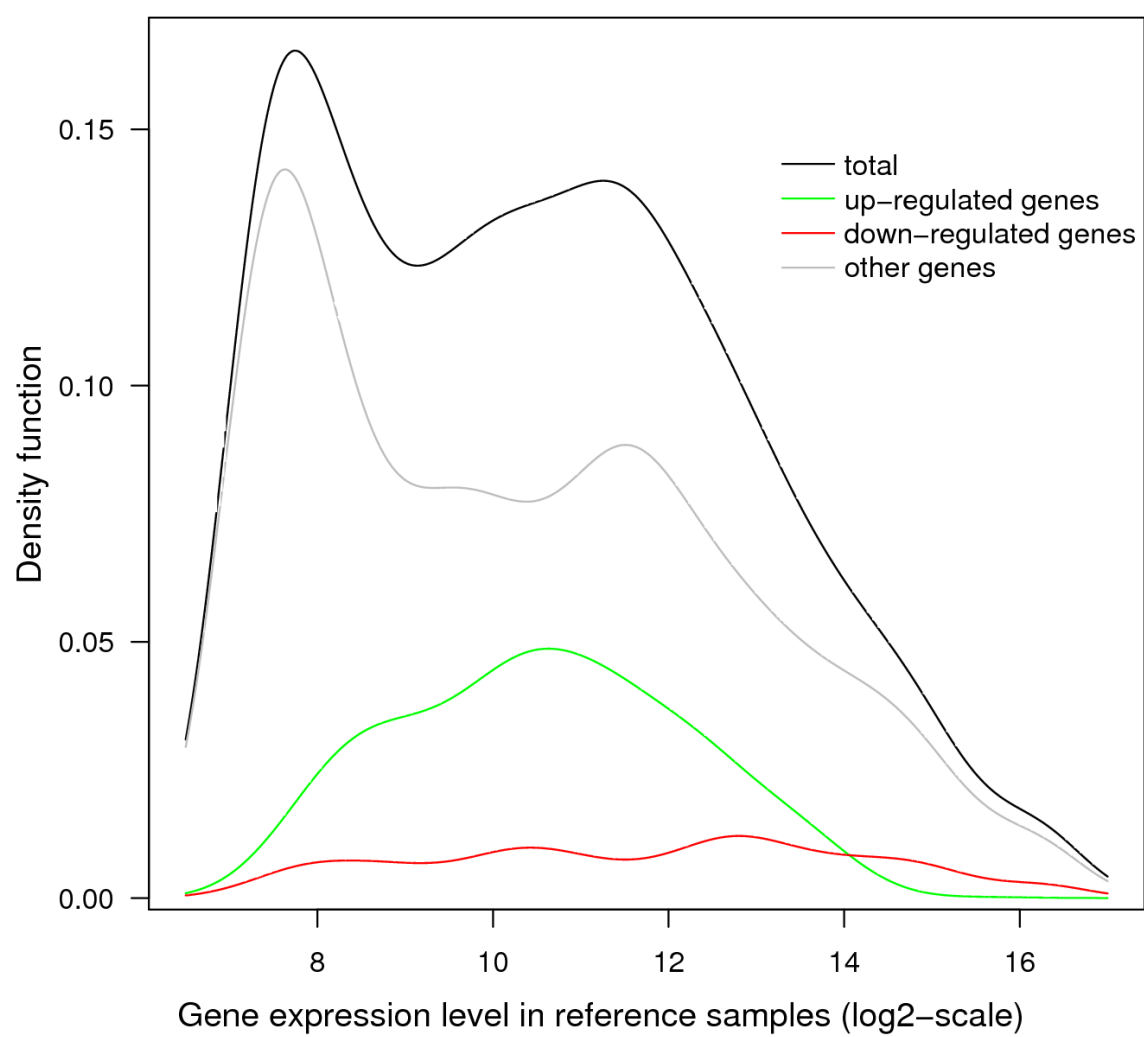

Supplement: Figure S2 — Detection of regulation by RNase Y is affected by the expression level in the reference samples. The black line shows the distribution of the expression level for the total repertoire of protein coding genes. The green, red and gray lines correspond respectively to the distribution of expression level for the three sub-categories: up-regulated, down-regulated and other genes. The fraction of up-regulated genes varies as a function of the gene expression level in the reference and reaches a maximum near 10.5, where up-regulated genes account for ∼35% of the total. At the same point down-regulated genes account for ∼7% of the total. Probability density functions were estimated by a kernel method implemented in function ‘density’ of R with bandwidth 0.5. Density functions for the three sub-categories (up-regulated genes, down-regulated genes, other genes) are scaled to be proportional to the fraction of genes in each category. (PDF) [file pone.0054062.s002.pdf]

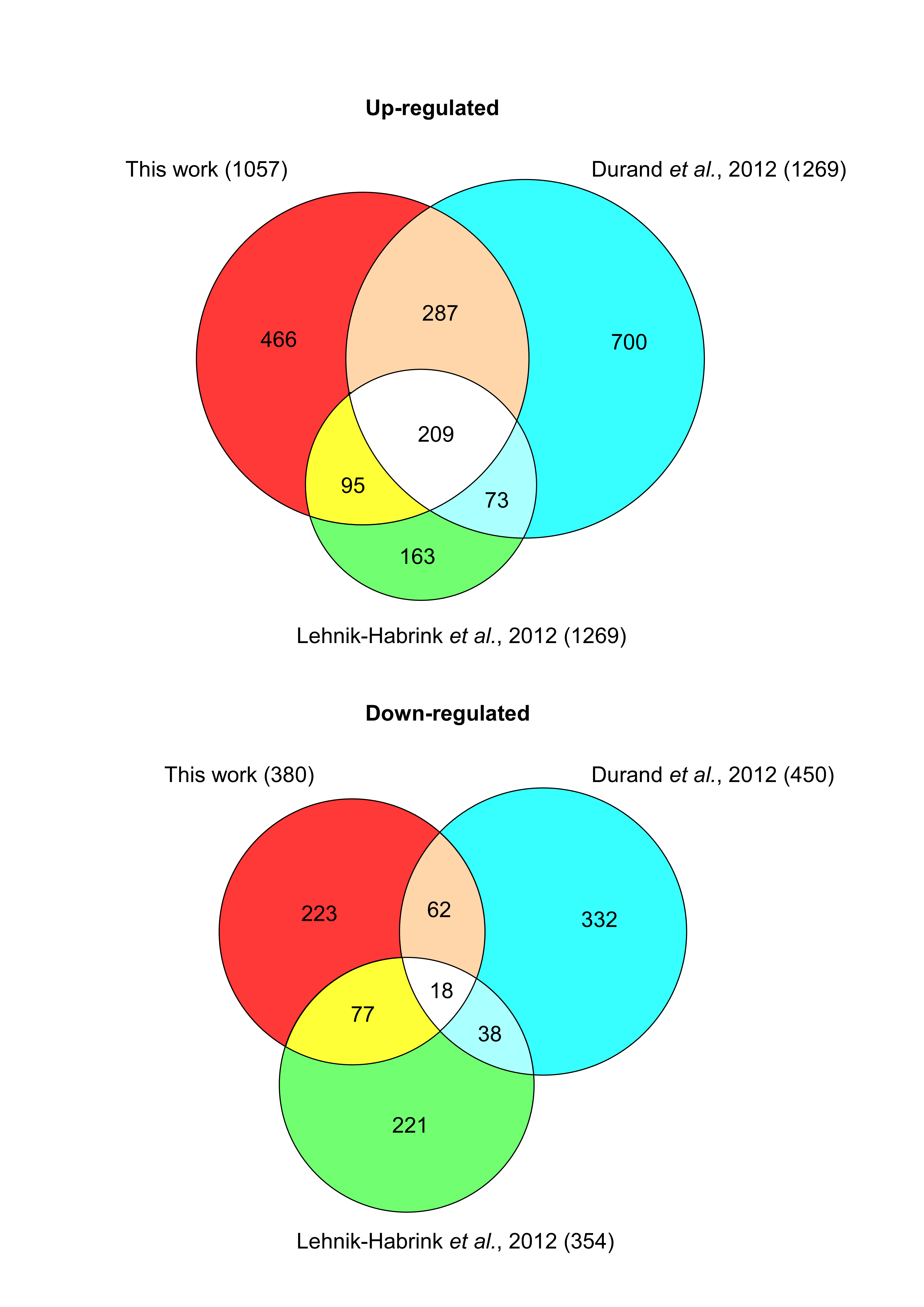

Supplement: Figure S3 — Effects of RNase Y depletion on the abundance of B. subtilis mRNAs. A comparison of three studies where (in contrast to Figure 3) a common arbitrary cut-off value of 1.5x has been applied. The Venn diagrams show the numbers of mRNAs altered following RNase Y depletion and highlights the number of mRNAs common to two or all three studies. The color code is as follows : Red, this work. Blue, a tiling array study by Durand et al. (2012). Green, a classical transcriptome study by Lehnik-Habrink et al. (2011). The numbers in parentheses indicate the total number of mRNAs considered to be altered in each study. (TIF) [file pone.0054062.s003.tif]
