# Supplementary material for: Bacillus subtilis RNase Y Activity In Vivo Analysed by Tiling Microarrays
Source: PLoS One. 2013 Jan 10;8(1):e54062. doi: 10.1371/journal.pone.0054062 (PMC3542257; doi:10.1371/journal.pone.0054062)
Supplement: Table S5 — Oligonucleotides used in this study. Hybridizing sequences in first PCR cycle are in upper case; non-hybridizing sequences are lower case. (DOCX) [file pone.0054062.s008.docx]

**Table S5. Oligonucleotides used in this study**

*Gene Name Sequence*

*adcA* HP1655 (5’) CAGCATACAGCTTTTGGCTATCTGG

HP1656 (3’) tcggaattcaattaatacgactcactataggATAGTGAATCTTTCAGGGCATCGAG

*dnaA* HP1602 (5’) gttaaggcctATGGAAAATATATTAGACCTGTGGAAC

HP1603 (3’) cgcaattgaattaatacgactcactataggCACTTTGGCAGAAGGATTATGATC

*oxdC* HP1609 (5’) GTTAGATATCTGACATTCCGCAGCCAATTAGAG

HP1610 (3’) atcggaattcaattaatacgactcactataggTGCCAGGCAAATTGGAAATCTC

*rnmV* HP1604 (5’) gttagatatcATGAAAATTAAAGAGATCATTGTGGTC

HP1605 (3’) atcggaattcaattaatacgactcactataggCATTCTGTTCCTCCCGCATAACGG

*tagD* HP1657 (5’) ATGAAAAAAGTTATCACATATG

HP1658 (3’) caattaatacgactcactataggTAAACCAGCAATTTCCTCTTTG

*ydzA* HP1629 (5’) gttagatatcCGGAAGACTTCGCACGATGG

HP1630 (3’) atcggaattcaattaatacgactcactataggAATTTCCGAATGGGACGAAAGC

*yitJ* (SAM) HP1231 (5’) GCTCGAACAGCTTGGAAGATAAG

HP1058 (3’) ttcaattaatacgactcactatagggAATGCCATAGGAGTAGAGGAGCGTC

*S72* HP1613 (5’) gttagatatcTACGTAACTTAATAATCGATGCAGATG

HP1614 (3’) atcggaattcaattaatacgactcactataggTTGAAGAGCTACCTTATTATTGGTC

*S977* HP1625 (5’) gttagatatcCCGCCGGGTCAGTATAAATGTTG

HP1626 (3’) atcggaattcaattaatacgactcactataggCTTTCAACATCTCAAGGGCGGTC

*S1458* HP1608 (5’) GTTAGATATCCTAACTGCCATAATAGCGGCGTCAGC

HP1609 (3’) atcggaattcaattaatacgactcactataggCAGATGAAGGGGAATATTTACAGC

*5S rRNA* HP246 ATCGGCGCTGAAGAGCTTAACTTCC

Hybridizing sequences in the first PCR cycle are in upper case; non-hybridizing sequences are lower case.
